# Copper Catalysts Anchored on Cysteine-Functionalized Polydopamine-Coated Magnetite Particles: A Versatile Platform for Enhanced Coupling Reactions

**DOI:** 10.3390/molecules29215121

**Published:** 2024-10-30

**Authors:** Yu-Jeong Jo, Seung-Woo Park, Ueon Sang Shin, Seung-Hoi Kim

**Affiliations:** 1 Department of Chemistry, Dankook University, Cheonan 31116, Republic of Korea; yujeong0424@naver.com (Y.-J.J.); dream1wh@naver.com (S.-W.P.); 2 Department of Nanobiomedical Science, BK21 FOUR NBM Global Research Center for Regenerative Medicine, Dankook University, Cheonan 31116, Republic of Korea; 3 Institute of Tissue Regeneration Engineering (ITREN), Dankook University, Cheonan 31116, Republic of Korea

**Keywords:** magnetite, cysteine-modified polydopamine, heterogeneous copper catalyst, Sonogashira coupling, click reaction

## Abstract

Cysteine plays a crucial role in the development of an efficient copper-catalyst system, where its thiol group serves as a strong anchoring site for metal coordination. By immobilizing copper onto cysteine-modified, polydopamine-coated magnetite particles, this advanced catalytic platform exhibits exceptional stability and catalytic activity. Chemical modification of the polydopamine (PDA) surface with cysteine enhances copper salt immobilization, leading to the formation of the Fe_3_O_4_@PDA-Cys@Cu platform. This system was evaluated in palladium-free, copper-catalyzed Sonogashira coupling reactions, effectively catalyzing the coupling of terminal acetylenes with aryl halides. Additionally, the Fe_3_O_4_@PDA-Cys@Cu platform was employed in click reactions, confirming the enhanced catalytic efficiency due to increased copper content. The reusability of the platform was further investigated, demonstrating improved performance, especially in recyclability tests in click reaction, making it a promising candidate for sustainable heterogeneous catalysis.

## 1. Introduction

Transition metal-catalyzed cross-coupling reactions have become increasingly prevalent in contemporary synthetic organic chemistry, facilitating the creation of vital carbon–carbon (C–C) bonds [1,2]. Within the spectrum of strategies for synthesizing biologically or industrially significant organic functional molecules, the Sonogashira coupling reaction emerges as a pivotal technique, notably facilitating the formation of carbon–carbon bonds, particularly in the synthesis of alkynes [3,4].

The coupling reaction between aromatic acetylenes and aryl halides was independently reported in 1975 by Cassar [5], Heck [6], and Sonogashira [7]. To perform those coupling reactions, a wide range of palladium catalysts, along with copper co-catalysts, have been frequently employed.

Although the reaction can be performed under mild conditions, applying the Sonogashira reaction to industrial or pharmaceutical synthesis became challenging due to the toxicity and air sensitivity of phosphine ligands, as well as the high price of palladium [8]. To overcome the shortcomings of the aforementioned reaction conditions, various approaches have focused on the development of more sustainable and cost-effective catalytic systems, such as palladium- and phosphine-free Sonogashira-type coupling reactions. In this context, copper has emerged as a promising candidate due to its earth abundance and low toxicity [9,10,11,12,13,14,15,16,17,18,19,20,21,22,23]. Along with copper metal, several transition metal complexes, including Fe [24,25,26], Zn [27], Co [28,29], Ag [30], Ni [31], Ru [32,33], and Au [34], have been efficiently utilized for the execution of Sonogashira coupling reactions.

Interestingly, recent scientific artworks have highlighted the growing interest in Pd-free copper-catalyzed Sonogashira coupling reactions, with the aim of overcoming the limitations associated with palladium-based catalysis [17,35,36]. More importantly, despite the impressive advancements in C_sp_–C_sp2_ bond construction utilizing various transition metal catalytic systems over the past few decades, the development of heterogeneous Cu-based catalyst systems remains an ongoing challenge. To date, several heterogeneous copper catalytic protocols, including appropriate rigid support, have been successfully developed and applied to Sonogashira coupling [36].

Of the aforementioned outcomes, utilizing the magnetic support attracted our attention [37,38,39]. In our previous study, we demonstrated the preparation and application of a heterogeneous copper catalytic platform, where copper was directly immobilized on the surface of polydopamine (PDA)-coated magnetite (Fe_3_O_4_@PDA@Cu) [40]. Although this platform demonstrated satisfactory performance as a heterogeneous copper catalyst in click reactions, we envisioned a higher copper content in this type of magnetite-based platform. To achieve this goal, we designed further modifications to the PDA surface of magnetite by introducing a biocompatible substance. Cysteine was chosen as the optimal compound for this decoration.

According to previous studies, cysteine, which contains amine, carboxy, and thiol groups, has been widely utilized for effective anchoring with PDA by covalently linking to PDA-modified core–shell nanoparticles for secondary modification [41,42,43]. As a result, the cysteine-modified PDA-magnetite particles played a significant role in removing Pb from wastewater [44]. Interestingly, a recent study demonstrated an example of cysteine-modified layered double hydroxides (LDHs)-coated magnetite nanoparticles for copper anchoring and its application in click reactions [45]. These outcomes strongly supported our anticipation of enhancing copper immobilization through the action of cysteine moiety on the surface of the Fe_3_O_4_@PDA-Cys platform.

Building on the concepts above, we developed a novel magnetically recoverable copper catalytic platform using cysteine as a biocompatible substrate (denoted as Fe_3_O_4_@PDA-Cys@Cu). Cysteine’s functional groups enhance copper-metal immobilization, making this platform highly effective in Pd-free Cu-catalyzed Sonogashira coupling reactions. Additionally, increased copper content was shown to enhance catalytic recyclability, as demonstrated in a comparative study of click reactions. This versatile platform offers promising potential for a range of organic coupling reactions.

## 2. Results

### 2.1. Characterization

Characterization of the obtained platform was performed using several techniques, including FT-IR, TGA, EDX, and SEM. According to the ICP-OES analysis, the cysteine-modified magnetite particles (Fe_3_O_4_@PDA-Cys@Cu) contained a higher copper content (18.96%) compared to the unmodified platform (Fe_3_O_4_@PDA@Cu), which had 5.02%.

**Figure 1:**
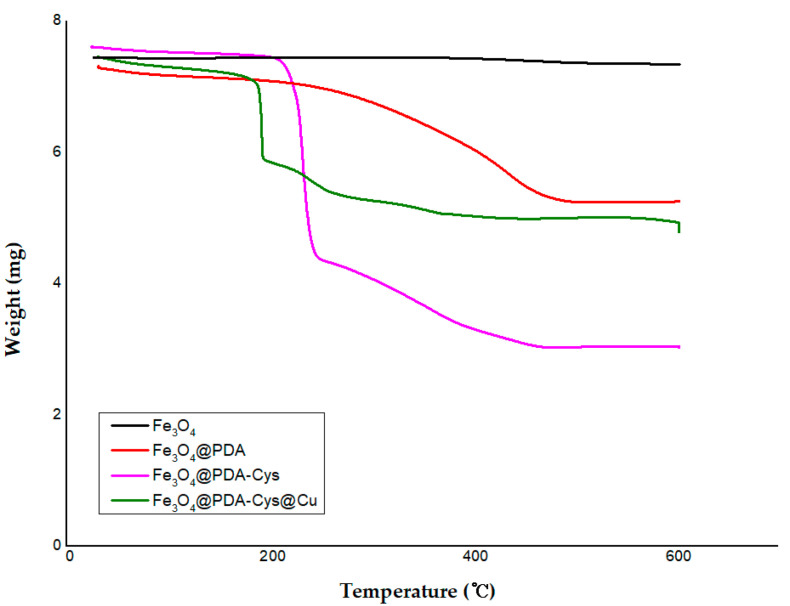
Comparing TGA analyses of Fe_3_O_4_@PDA-Cys@Cu platform and related substrates.

The thermal stability of the Fe_3_O_4_@PDA-Cys@Cu platform and related complexes was investigated using TGA (Figure 1). The negligible weight loss below 150 °C is attributed to the physically adsorbed volatile substances. Upon comparing the TGA analysis results, the difference between the Fe_3_O_4_@PDA@Cu and the cysteine-modified platform (Fe_3_O_4_@PDA-Cys@Cu) becomes even more pronounced. Interestingly, a very similar pattern was observed in the cysteine-modified platforms (Fe_3_O_4_@PDA-Cys and Fe_3_O_4_@PDA-Cys@Cu).

When examining the weight changes in the Fe_3_O_4_@PDA-Cys platform, the weight loss of cysteine occurred rapidly, whereas PDA showed a gradual weight loss attributed to the polymerized form of dopamine. A very similar two-stage weight loss was observed in the thermogram for the Fe_3_O_4_@PDA-Cys@Cu platform, which was prepared using the Fe_3_O_4_@PDA-Cys platform. These weight losses indicate the presence of PDA and cysteine grafted to the Fe_3_O_4_ surface. Based on the Fe_3_O_4_@PDA-Cys platform, a brief calculation of the composition ratio of each component showed that Fe_3_O_4_, PDA, and cysteine were present in a mole ratio of approximately 1:2:2.

**Figure 2:**
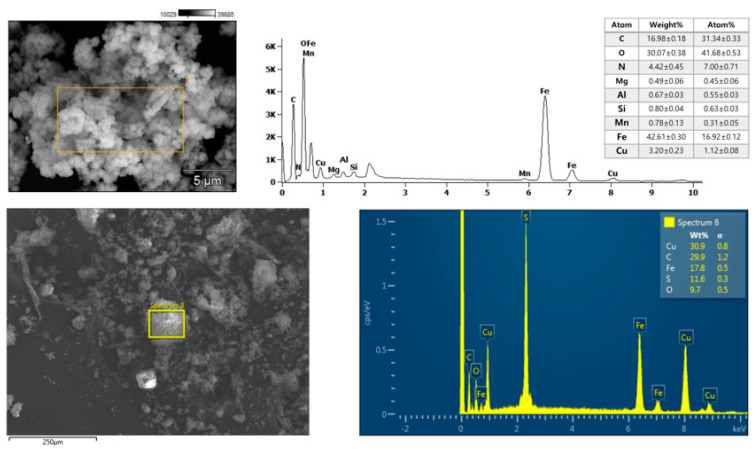
EDX analyses of Fe_3_O_4_@PDA@Cu (up) and Fe_3_O_4_@PDA-Cys@Cu (down).

Furthermore, a morphological feature of the Fe_3_O_4_@PDA-Cys@Cu was investigated using SEM–EDX and is provided in Figure 2. The EDX analyses of each platform clearly showed an increase in copper content in the Fe_3_O_4_@PDA-Cys platform compared to the Fe_3_O_4_@PDA platform.

### 2.2. Application to Organic Reactions

To verify the catalytic activity of the prepared Fe_3_O_4_@PDA-Cys@Cu platform, it was initially employed in the Sonogashira coupling reaction, a cross-coupling reaction involving aryl acetylenes and aryl halides.

Before the general application of the Fe_3_O_4_@PDA-Cys@Cu platform, we conducted a preliminary search for optimal parameters for the Sonogashira coupling reactions using phenyl acetylene (**1a**) and 4-iodoanisole (**2a**) as standard substrates (Table 1).

At first, the model reaction was carried out using excess equivalent (1.2 eq) of phenyl acetylene (**1a**) with 60 mg of Fe_3_O_4_@PDA-Cys@Cu platform in DMSO at 100 °C, affording the desired coupling product (**3a**) in 84% isolated yield (Table 1, Entry 1). Significantly, a remarkable improvement in isolated yield (up to 99%) was achieved by increasing the amount of the catalytic platform (up to 80 mg) employed under the same conditions (Table 1, Entry 2). Subsequent trials were conducted to identify a more versatile solvent system, which demonstrated the high efficiency of both ethanol and water as reaction media. For practical convenience, we chose to employ EtOH as the reaction solvent for further investigation. Decreasing the amount of the platform to 60 mg did not significantly affect the isolated yield (Table 1, Entries 5 and 6). Further attempts with 50 mg of Fe_3_O_4_@PDA-Cys@Cu platform demonstrated relatively lower catalytic efficiency under similar conditions (Table 1, Entry 7). The use of equimolar amounts of **1a** and **2a** resulted in a slightly disappointing outcome (Table 1, Entry 8). Interestingly, reaction temperature turned out to be a critical factor for the successful completion of the reaction (Table 1, Entry 9).

With the optimization tests, the scope and applicability of the novel catalytic platform were further explored using various substrates (Table 2). Firstly, the investigation was conducted using **1a** and aryl halides containing a C–I bond in the presence of Fe_3_O_4_@PDA-Cys@Cu platform under standard conditions. Initially, 2-iodotoluene (**2b**) was reacted with **1a**, resulting in the formation of 1-methyl-2-(phenylethynyl)benzene (**3b**) with an isolated yield of 91% (Table 2, Entry 1). Additionally, the reaction of **1a** with 3-fluoro-iodobenzene proceeded well, yielding the corresponding coupled product (**3c**) in excellent isolated yields (Table 2, Entry 2). In contrast to these positive outcomes, relatively lower performance was observed in the coupling reactions with 4-chloroiodobenzene (**2d**) and 3-cyano-iodobenzene (**2e**). Interestingly, a polar functional group (OH) on aryl iodide (**2f**) was well tolerated, yielding the desired product (**3f**) in excellent yield (Table 2, Entry 5).

The substrate scope of acetylene was also investigated using various aryl acetylenes (**1b**–**1f**) under standard conditions. Overall, the reaction outcomes were consistent with those observed for **1a**, confirming the catalytic efficiency of the developed reaction platform. Entries 6 and 7 in Table 2 demonstrate that 3-iodoaniline (**2g**) and 3-fluoro-iodobenzene (**2c**) were effective substrates for the synthesis of their respective asymmetric acetylene derivatives (**3g** and **3h**) from 1-ethynyl-4-methylbenzene (**1b**) using the optimized Sonogashira coupling (Table 2, Entries 6 and 7). Similar catalytic performance was observed when various aryl acetylenes, such as 4-ethynylbenzonitrile (**1c**) and 1-ethynyl-4-methoxybenzene (**1d**), were used. The corresponding products (**3i** and **3j**, respectively) were effectively obtained with excellent yields (Table 2, Entries 8 and 9). Furthermore, the presence of bromine (**1e**) and hydroxyl (**1f)** groups on the aryl acetylene rings did not affect the reaction outcome (Table 2, Entries 10 and 11). The reaction conditions used in this study were well tolerated to provide the final coupling products (**3k** and **3l**) in an excellent manner.

To further extend the scope of this green protocol, we studied the coupling of **1a** with various aryl halides bearing a C–Br bond, including 4-bromobenzonitrile, 2-bromothiophene, and 2-bromopyridine. Unfortunately, the reactions proceeded sluggishly or resulted in inseparable mixtures (Table 2, Entries 12–14).

Next, the recyclability of the novel catalytic platform was explored to ensure its advantage as a heterogeneous catalytic system (Table 3). Following standard procedures, a recycling test was carried out using **1a** and **2a**. After each cycle, the catalyst was effortlessly retrieved using an external magnet, washed consecutively with fresh water and acetone, and subsequently dried in air. The results from the recycling test indicate that the present platform demonstrates high potential as a recoverable and reusable heterogeneous catalytic system for Sonogashira coupling under mild conditions.

As mentioned before, we previously disclosed a novel heterogeneous copper catalyst immobilized on polydopamine-coated magnetite for click reaction [40]. In our previous study, the catalyst was prepared by immobilizing Cu(OAc)_2_ salt onto polydopamine-coated magnetite (denoted as Fe_3_O_4_@PDA@Cu). The catalytic activity of the resulting Fe_3_O_4_@PDA@Cu platform was investigated in a click reaction, utilizing three-component reactions of azide, alkyne, and benzyl surrogates in water, which provided the corresponding 1,2,3-triazoles in high yields. Despite the positive outcomes using the Fe_3_O_4_@PDA@Cu platform, unsatisfactory results were observed in recycling tests (see Table 5).

Despite numerous approaches utilizing magnetite-based copper catalytic systems for click reaction [40], there remains a need for versatile routes that focus on catalyst recyclability and reusability. Here, to expand the library of click reactions catalyzed by magnetic nanoparticle-supported copper catalysts and conduct a comparative study, we further carried out click reactions using various substrates in the presence of our newly designed Fe_3_O_4_@PDA-Cys@Cu platform. For a simple comparison, the results were disclosed in parallel with those obtained using our previous Fe_3_O_4_@PDA@Cu platform, as shown in Table 4.

In general, no significant differences were observed in the overall reactivity when using Fe_3_O_4_@PDA@Cu and Fe_3_O_4_@PDA-Cys@Cu as catalysts in the click reaction, respectively. In both cases, cyclization proceeded smoothly, providing 1,4-disubstituted-1,2,3-triazoles in excellent yields regardless of the functionality of acetylenes or benzyl halides. As evidenced by the yield of the target product, each catalyst system exhibited highly effective catalytic activity in the click reaction.

Next, the recyclability of the novel catalytic platform in click reaction was explored to ensure its advantage over our previous non-modified Fe_3_O_4_@PDA@Cu platform [41]. Following standard procedures, two different platforms—non-cysteine-modified (Fe_3_O_4_@PDA@Cu) and cysteine-modified (Fe_3_O_4_@PDA-Cys@Cu)—were employed in a recycling test (Table 5). The coupling of phenyl acetylene (**1a**), benzyl bromide, and sodium azide was selected for the recycling test, and the efficiency of each platform was verified by comparing the isolated yields of the coupling product (**4a**), which are comparatively displayed. As shown in Table 5, the catalytic activity of Fe_3_O_4_@PDA@Cu and Fe_3_O_4_@PDA-Cys@Cu in recycling tests for the click reaction demonstrated significant differences, clearly indicating the superior effectiveness of the Fe_3_O_4_@PDA-Cys@Cu catalyst. In contrast, the Fe_3_O_4_@PDA@Cu catalyst, as used in our previous study, showed a much more limited catalytic recycling ability, further highlighting the improved performance of the cysteine-modified platform in these reactions.

We hypothesized that these differences could be attributed to the variation in copper content retained within the recycled platforms after use. To verify our assumption, we performed an ICP analysis to quantify the decrease in copper content before and after use. In our previous study with Fe_3_O_4_@PDA@Cu, the copper content dropped from 5.01% to 0.04%. Interestingly, in the current study using Fe_3_O_4_@PDA-Cys@Cu, the reduction in copper content was notably less, decreasing from 18.96% to 17.41% after seven recycling cycles.

**Figure 3:**
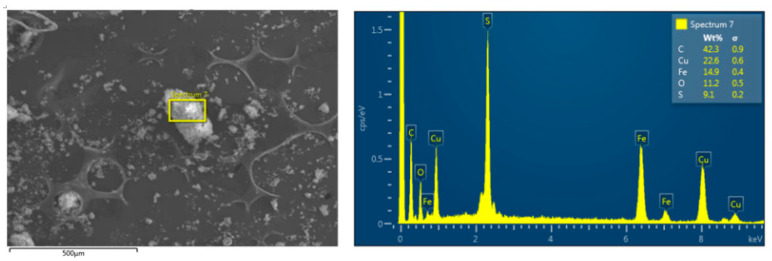
EDX analysis of recovered Fe_3_O_4_@PDA-Cys@Cu.

Furthermore, a similarly modest reduction in copper content was also observed during the Sonogashira coupling reaction using Fe_3_O_4_@PDA-Cys@Cu and confirmed by EDX analysis (Figure 3).

## 3. Conclusions

We developed and thoroughly characterized a novel, recoverable catalytic platform composed of magnetic particles (Fe_3_O_4_), copper salts, polydopamine, and cysteine (Fe_3_O_4_@PDA-Cys@Cu). Using a range of spectroscopic methods, including FT-IR, EDX, TGA, SEM, and ICP-OES, we confirmed the enriched copper content in this platform. The enhanced immobilization of copper salt is attributed to the cysteine modification of the PDA surface on the magnetite. 

In application, the increased copper content proved highly effective in organic reactions. For example, the Fe_3_O_4_@PDA-Cys@Cu platform successfully facilitated the Sonogashira coupling reaction, yielding the desired C_sp_–C_sp2_ coupling products under mild conditions. Various types of aryl acetylenes and aryl iodides were suitable for the coupling reaction, although aryl halides with carbon–bromine bonds showed limitations. Furthermore, the increased copper content in the platform enhanced its catalytic efficiency in click reactions, particularly in recycling tests. A three-component reaction involving terminal alkynes, benzyl surrogates, and sodium azide in an aqueous environment yielded 1,4-disubstituted-1,2,3-triazoles in good to excellent isolated yields. Notably, the recycling test highlighted the clear advantage of cysteine modification in the PDA-coated magnetite-based nanoparticle platform. Compared to our previous study [40], the reusability of the platform was significantly improved up to the seventh recycling run.

In conclusion, the biocompatible material cysteine played a key role in enhancing copper immobilization during the preparation of a magnetically recoverable catalytic platform. This modified copper-based platform exhibited significantly enhanced catalytic activity in both Sonogashira coupling and click reactions.

## 4. Materials and Methods

### 4.1. Procedure for Fe_3_O_4_@PDA

Fe_3_O_4_ nanoparticles were prepared using the co-precipitation method, following the literature [46,47]. An iron salt solution was obtained by mixing FeCl_3_·6H_2_O (34.6 g) and FeCl_2_·4H_2_O (12.7 g), in the molar ratio of 2:1, in 1.5 L of deionized water under nitrogen at room temperature. By dropwise addition of NH_3_ solution, the pH was adjusted to 12. A black precipitate was formed after continuously stirring for 1 h. The precipitate was magnetically separated and washed four times with deionized water until the solution reached a pH of 8, followed by washing with ethanol (30 mL × 2). Then, the resulting precipitate was dried under vacuum at 50 °C overnight. 

Next, for the PDA-coating of magnetite, a mixture of Fe_3_O_4_ MNPs (5.0 g) and tris buffer (1.0 M, pH 8.5, 50 mL) was sonicated for 30 min. Subsequently, dopamine hydrochloride (10.0 g) was added to the mixed solution, and the mixture was stirred at room temperature for 24 h. Then, the resulting black particles (Fe_3_O_4_@PDA) were collected using a magnet and washed with H_2_O (30 mL × 3) and ethanol (30 mL × 2). The obtained platform was dried in vacuum at 50 °C for 12 h.

### 4.2. Procedure for Fe_3_O_4_@PDA-Cys

A solution of Fe_3_O_4_@PDA particles (3.0 g) in H_2_O (200 mL) was sonicated for 3 h at room temperature. In a separate flask, L-cysteine (4.5 g) in H_2_O (100 mL) was sonicated for 1 h at room temperature. Next, the cysteine solution was then added to the Fe_3_O_4_@PDA solution, and the resulting mixture was stirred at 50 °C for 24 h. The black precipitates were filtered, washed sequentially with H_2_O (30 mL × 3) and EtOH (30 mL × 3), and then dried under vacuum at 50 °C for 24 h, providing 5.0 g of the Fe_3_O_4_@PDA-Cys platform.

### 4.3. Procedure for Fe_3_O_4_@PDA-Cys@Cu

A solution of Fe_3_O_4_@PDA-Cys particles (1.4 g) in EtOH/H_2_O (1:1, *v*/*v*, 80 mL) was sonicated for 1 h at room temperature. In a separated flask, copper acetate (1.4 g) was added and then sonicated for 30 min. Next, the two solutions were then combined, and the resulting mixture was stirred at room temperature for 24 h. The obtained particles were collected using a magnet and washed with water (20 mL × 3) and ethanol (20 mL × 3). After drying under vacuum at 50 °C for 12 h, 1.1 g of Fe_3_O_4_@PDA-Cys@Cu was obtained.

### 4.4. General Procedure for Sonogashira Coupling

To a round-bottom flask equipped with a magnetic bar, phenyl acetylene (**1a**, 0.36 g, 3.6 mmol), 4-iodoanisole (**2a**, 0.7 g, 3.0 mmol), Fe_3_O_4_@PDA-Cys@Cu (60 mg), and EtOH (4.0 mL) were added. Then, the mixture was stirred at a refluxing temperature. After completion, the reaction mixture was cooled down to room temperature. The mixture was filtered, and the filtrate was acidified with 3 M HCl aqueous solution. The aqueous layer was extracted with diethyl ether (3 × 10 mL). The combined organic layers were washed with brine, dried with anhydrous Na_2_SO_4_, and evaporated under reduced pressure. The crude mixture was purified by column chromatography on silica gel using hexanes only, yielding 0.61 g of 1-methoxy-4-(phenylethynyl)benzene (**3a**) as a white solid; m.p. 92–93 °C. ^1^H NMR (400 MHz, DMSO-d6) δ (ppm): 7.49–7.94 (m, 4H), 7.43–7.39 (m, 3H), 7.00–6.98 (dd, *J* = 6.8, 2.0 Hz, 2H), and 3.80 (s, 3H). ^13^C NMR (100 MHz, DMSO-d6) δ (ppm): 160.0, 133.4, 131.6, 129.2, 128.9, 123.1, 114.9, 114.7, 89.9, 88.4, and 55.7.

### 4.5. General Procedure for Click Reaction

The mixture of benzyl bromide (0.51 mg, 3.0 mmol), phenyl acetylene (0.37 mg, 3.6 mmol), and sodium azide (0.23 mg, 3.6 mmol) was stirred at 90 °C in the presence of Fe_3_O_4_@PDA-Cys@Cu platform (70 mg) in H_2_O. After being refluxed for 5.0 h, the mixture was decanted with the aid of an external magnet. The aqueous layer was then extracted with ethyl acetate (3 × 10 mL). The combined organic layers were washed with brine, dried with anhydrous Na_2_SO_4_, and evaporated under reduced pressure. The crude mixture was purified by column chromatography on silica gel [hexanes (90%)/ethyl acetate (10%)], yielding 0.69 g of 1-benzyl-4-phenyl-1*H*-1,2,3-triazole (**4a**) as a white solid; m.p. 127–128 °C. ^1^H NMR (400 MHz, CDCl_3_) δ (ppm): 7.69–7.66 (m, 2H), 7.56 (s, 1H), 7.29–7.22 (m, 5H), 7.21–7.16 (m, 3H), and 5.41 (s, 2H). ^13^C NMR (100 MHz, CDCl_3_) δ (ppm): 148.2, 134.8, 130.6, 129.1, 128.8, 128.7, 128.2, 128.0, 125.7, 119.7, and 54.1.

## Figures and Tables

**Table 1:** Screening optimal parameters for the Sonogashira coupling.

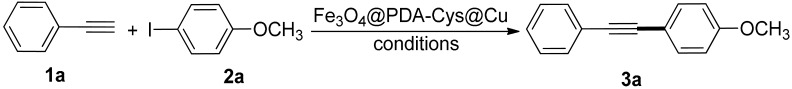							
Entry	1a	2a	Cu-Platform	Solvent	Temp.	Time	Yield ^a^
1	3.6 mmol	3.0 mmol	60 mg	DMSO	100 °C	4 h	84%
2	3.6 mmol	3.0 mmol	80 mg	DMSO	100 °C	4 h	99%
3	3.6 mmol	3.0 mmol	80 mg	EtOH	reflux	5 h	99%
4	3.6 mmol	3.0 mmol	80 mg	H_2_O	reflux	5 h	99%
5	3.6 mmol	3.0 mmol	70 mg	EtOH	reflux	5 h	99%
6	3.6 mmol	3.0 mmol	60 mg	EtOH	reflux	5 h	97%
7	3.6 mmol	3.0 mmol	50 mg	EtOH	reflux	5 h	93%
8	3.0 mmol	3.0 mmol	80 mg	EtOH	reflux	4 h	87%
9	3.6 mmol	3.0 mmol	70 mg	EtOH	rt	24 h	trace

^a^ Isolated yield of **3a** based on **2a.**

**Table 2:** Sonogashira coupling.

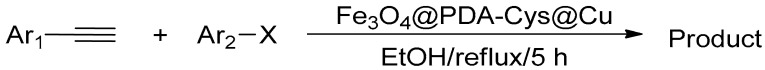				
Entry	Ar_1_	Ar_2_	Product	Yield ^a^
1	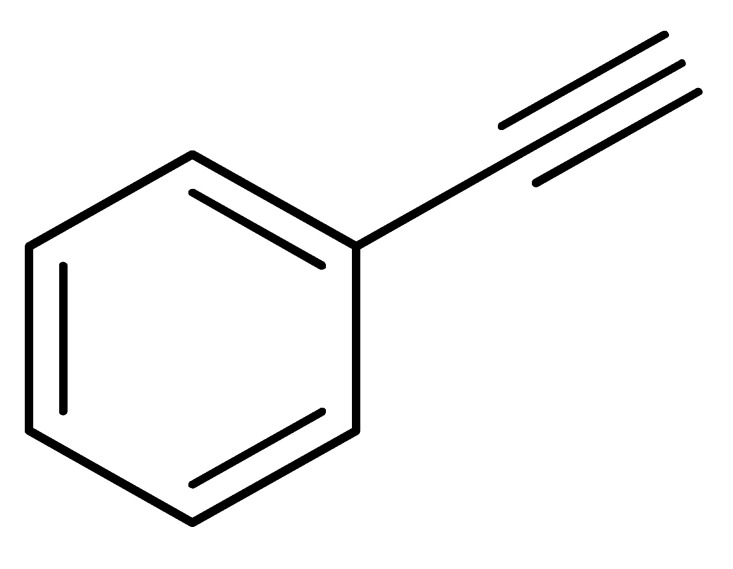 (**1a**)	** 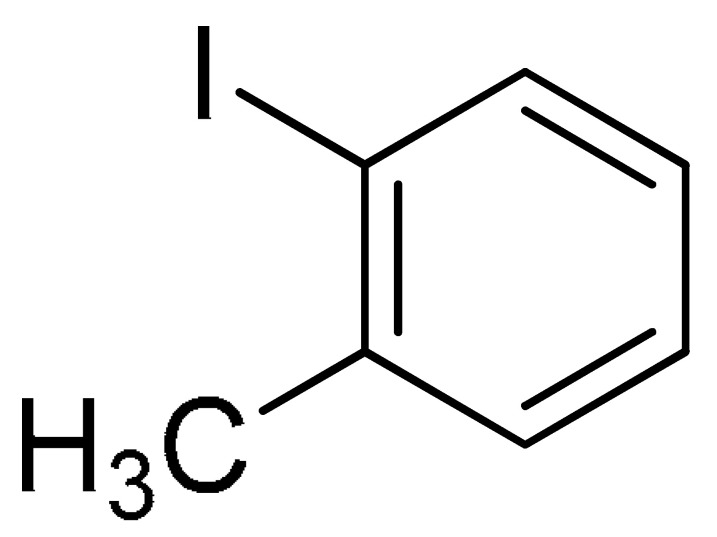 ** (**2b**)	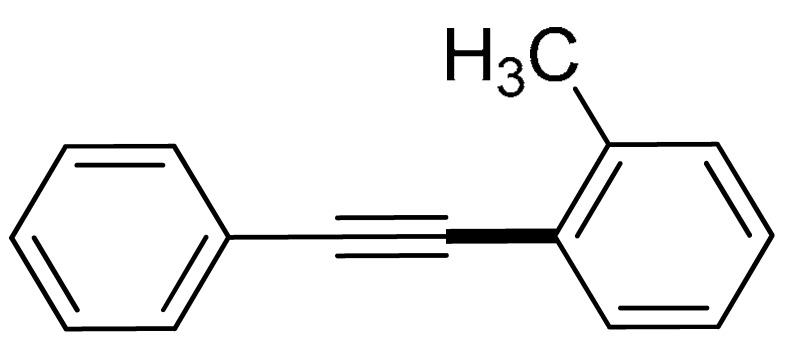 (**3b**)	91%
2	(**1a**)	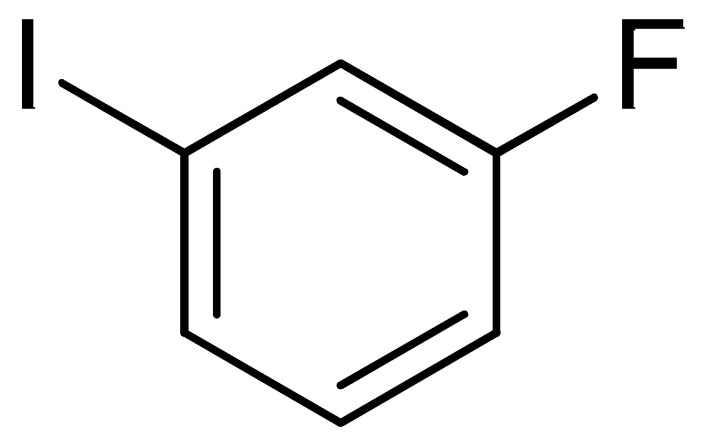 (**2c**)	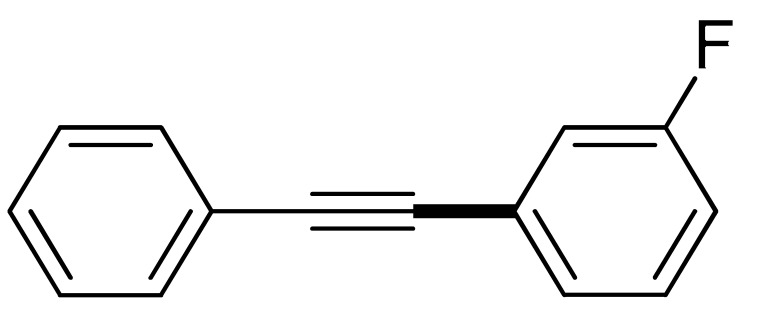 (**3c)**	93%
3	(**1a**)	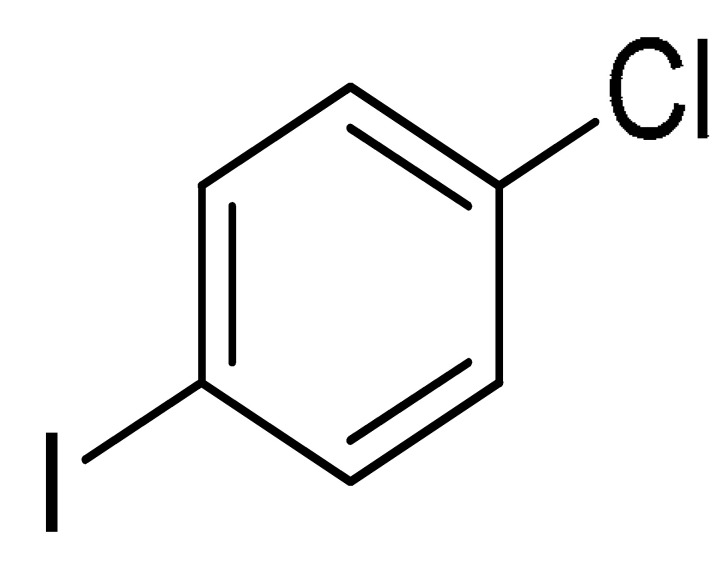 (**2d**)	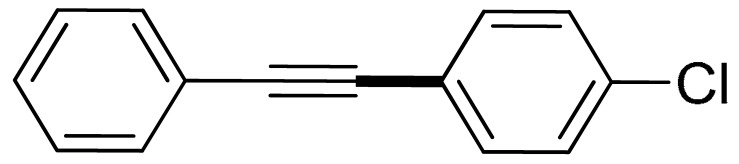 (**3d)**	86%
4	(**1a**)	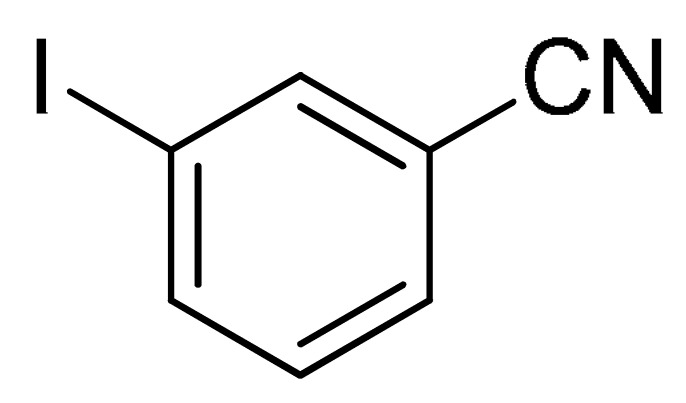 (**2e**)	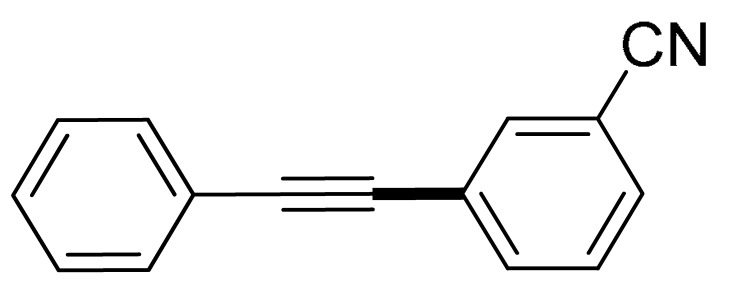 (**3e)**	88%
5	(**1a**)	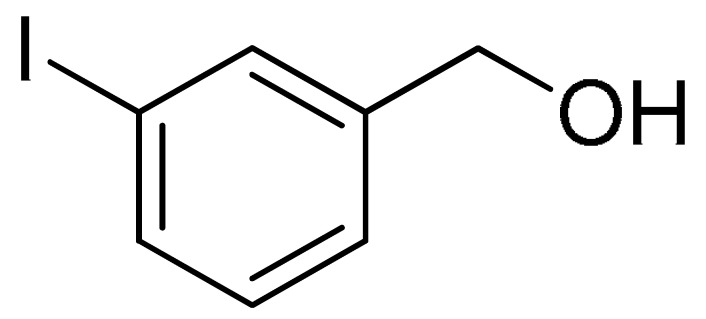 (**2f**)	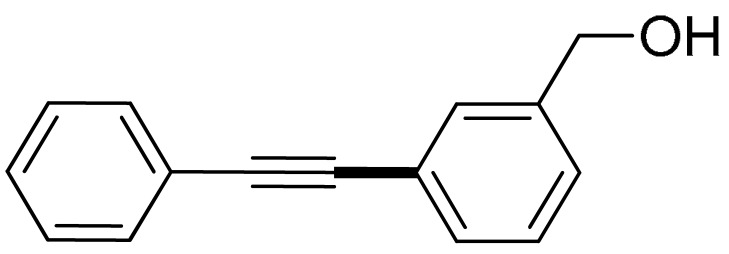 (**3f**)	98%
6	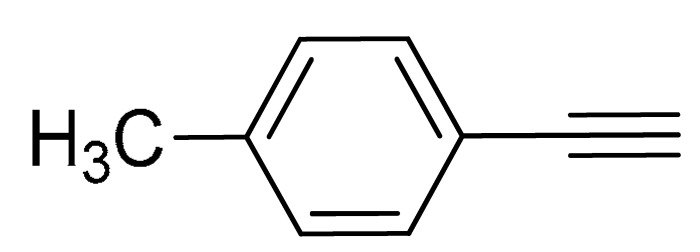 (**1b**)	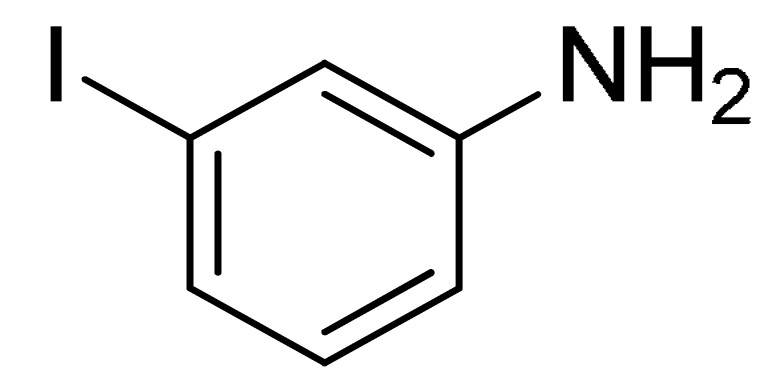 (**2g**)	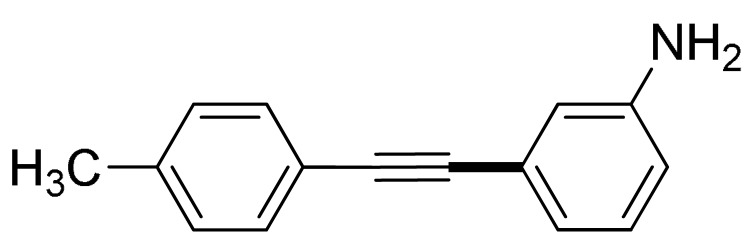 (**3g**)	98%
7	(**1b**)	(**2c**)	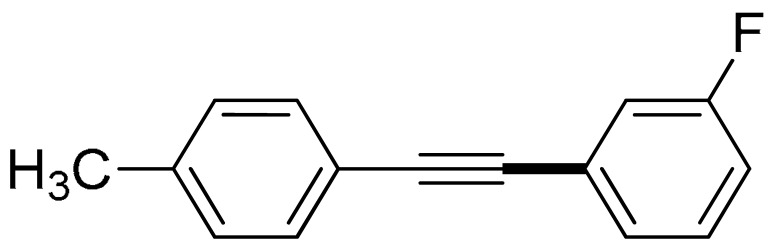 (**3h**)	92%
8	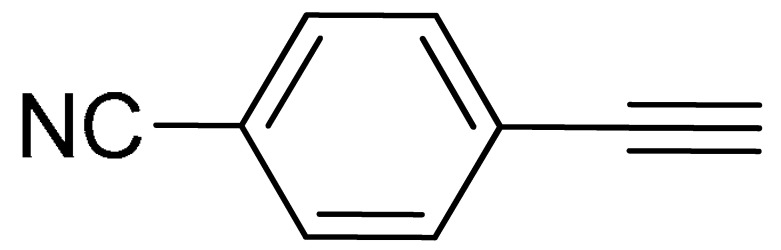 (**1c**)	(**2g**)	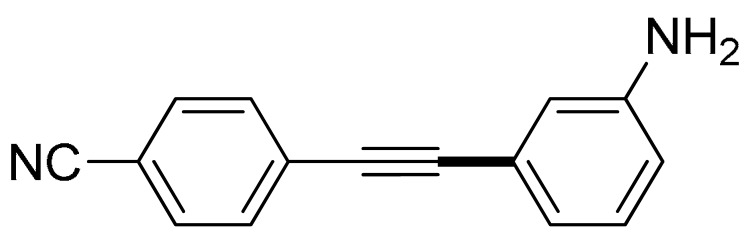 (**3i**)	94%
9	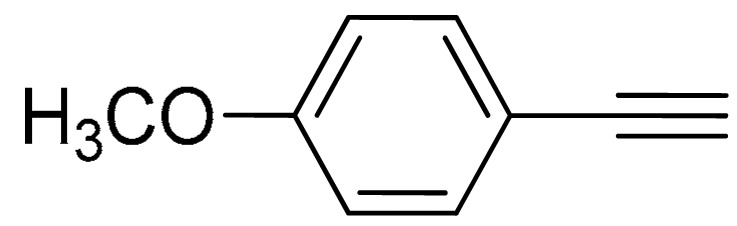 (**1d**)	(**2f**)	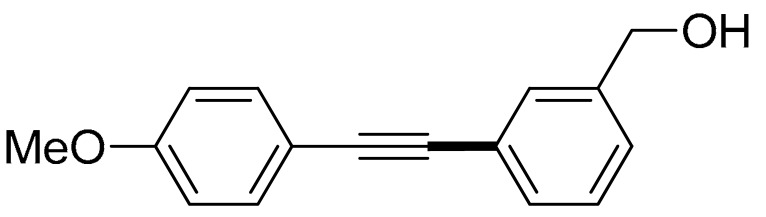 (**3j**)	91%
10	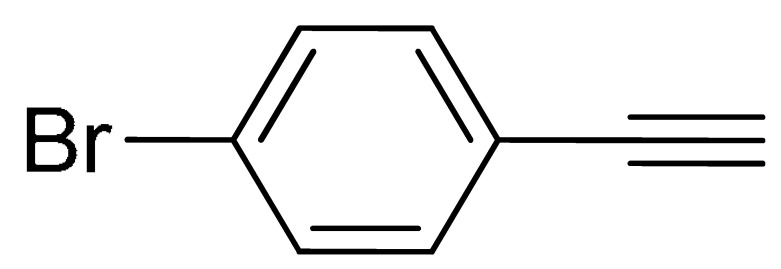 (**1e**)	(**2b**)	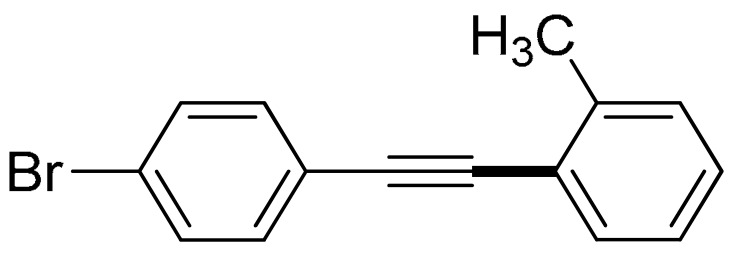 (**3k**)	95%
11	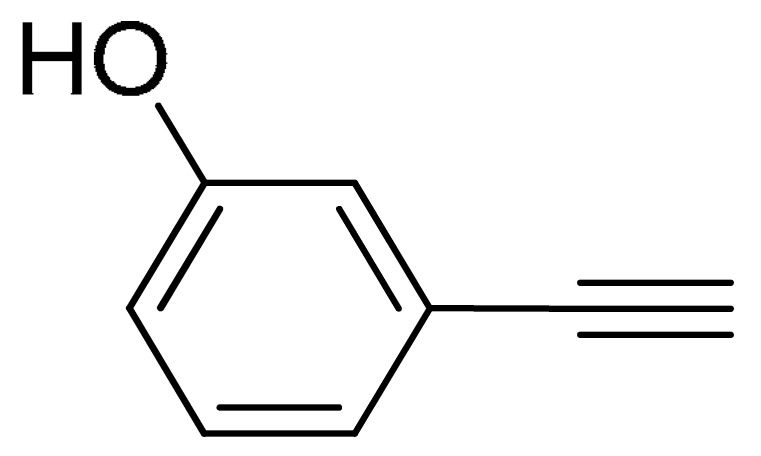 (**1f**)	(**2g**)	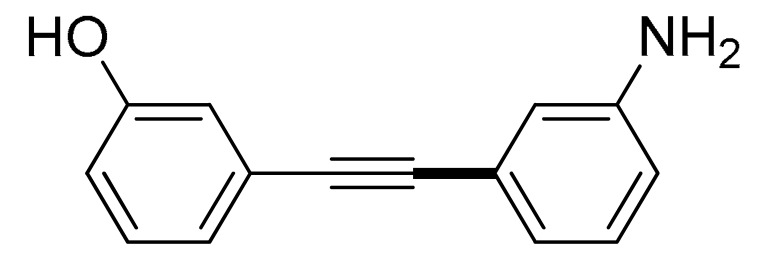 (**3l**)	96%
12	(**1a**)	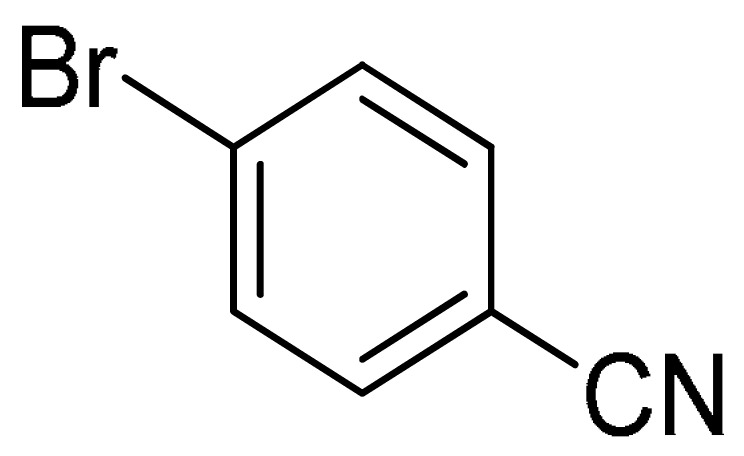	No reaction	-
13	(**1a**)	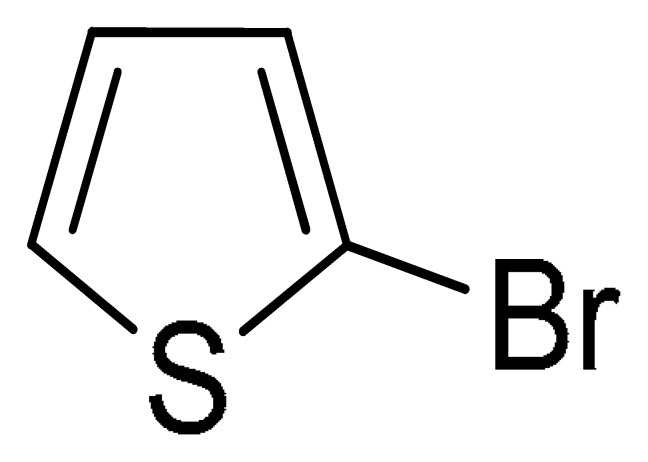	No reaction	-
14	(**1a**)	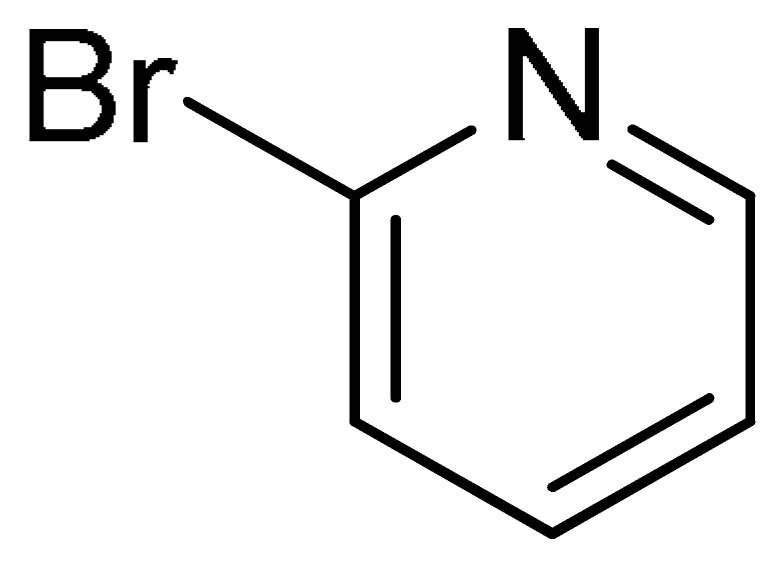	No reaction	-

^a^ Isolated yield based on aryl halide.

**Table 3:** Recyclability test in Sonogashira coupling.

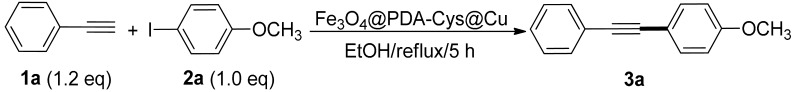							
Recycle	1st	2nd	3rd	4th	5th	6th	7th
Yield ^a^	98%	98%	96%	97%	95%	94%	90%

^a^ Isolated yield based on **2a**.

**Table 4:** Click reaction using Fe_3_O_4_@PDA-Cys@Cu.

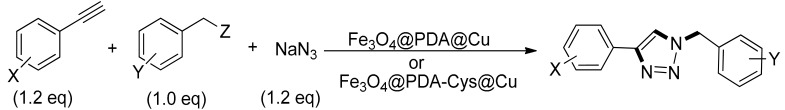						
Entry	X	Y	Z	Product	Result ^a^	Result ^b^
1	H	4-Br	Br	**4b**	80%	95%
2	H	4-*^t-^*Bu	Br	**4c**	88%	98%
3	H	4-F	Cl	**4d**	90%	90%
4	H	4-CN	Cl	**4e**	85%	90%
5	H	2,4-Cl_2_	Cl	**4f**	95%	96%
6	4-F	H	Br	**4g**	90%	96%
7	4-C_4_H_9_	H	Br	**4h**	86%	97%
8	4-OCH_3_	H	Br	**4i**	96%	98%
9	4-NO_2_	H	Br	**4j**	90%	91%
10	3-OH	H	Br	**4k**	88%	95%
11	4-NO_2_	4-F	Cl	**4l**	88%	92%
12	4-F	2,4-Cl_2_	Cl	**4m**	80%	92%
13	3-CH_3_	4-*^t-^*Bu	Br	**4n**	96%	97%

^a^ Isolated yield based on benzyl halide using Fe_3_O_4_@PDA@Cu platform [results cited from reference [40]]. ^b^ Isolated yield based on benzyl halide using Fe_3_O_4_@PDA-Cys@Cu platform [this work].

**Table 5:** Reusability test using the Fe_3_O_4_@PDA@Cu and Fe_3_O_4_@PDA-Cys@Cu platforms.

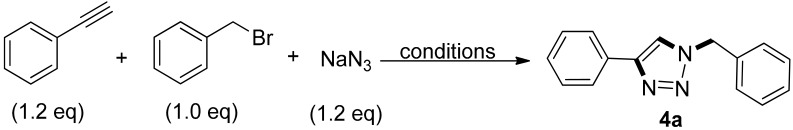								
Recycling	0	1st	2nd	3rd	4th	5th	6th	7th
Yield ^a^	98% (5 h)	97% (7 h)	72% (7 h)	51% (7 h)	-	-	-	-
Yield ^b^	99%	99%	96%	96%	95%	95%	95%	90%

^a^ Isolated yield of **4a** (based on **2a**) using Fe_3_O_4_@PDA@Cu platform. The number in parentheses indicates the reaction time. ^b^ Isolated yield of **4a** (based on **2a**) using Fe_3_O_4_@PDA-Cys@Cu platform. Each run was conducted in 5 h.

## Data Availability

Data are contained within the article and Supplementary Materials.

## Supplementary Materials

The following supporting information can be downloaded at https://www.mdpi.com/article/10.3390/molecules29215121/s1, ^1^H and ^13^C NMR data and copies of spectra. Reference [48] is cited in the supplementary materials.
