# Supplementary material for: Copper Catalysts Anchored on Cysteine-Functionalized Polydopamine-Coated Magnetite Particles: A Versatile Platform for Enhanced Coupling Reactions
Source: Molecules. 2024 Oct 30;29(21):5121. doi: 10.3390/molecules29215121 (PMC11547350; doi:10.3390/molecules29215121)
Supplement: Supplementary file 1 [file molecules-29-05121-s001.zip › molecules-3265351-supplementary.pdf]

Supporting Information for

Copper Catalysts Anchored on Cysteine-Functionalized Polydopamine-Coated Magnetite Particles: A Versatile Platform for Enhanced Coupling Reactions

Yu-Jeong Jo 1, Seung-Woo Park 1, Ueon Sang Shin 2,3,* and Seung-Hoi Kim 1,*

[1] Department of Chemistry, Dankook University, Cheonan 31116, Republic of Korea; yujeong0424@naver.com (Y.-J.J.); dream1wh@naver.com (S.-W.P.)

[2] Department of Nanobiomedical Science, BK21 FOUR NBM Global Research Center for Regenerative Medicine, Dankook University, Cheonan 31116, Republic of Korea

[3] Institute of Tissue Regeneration Engineering (ITREN), Dankook University, Cheonan 31116, Republic of Korea

* Correspondence: usshin12@dankook.ac.kr (U.S.S.); kimsemail@dankook.ac.kr (S.-H.K.)

Table of Contents

1.	General considerations	S1
2.	FT-IR spectra	S2
3.	^{1}H and ^{13}C NMR data of Sonogashira coupling products	S2
4.	Copies of ^{1}H and ^{13}C NMR spectra of Sonogashira coupling products	S4
5.	^{1}H and ^{13}C NMR data of triazoles	S16
6.	Copies of ^{1}H and ^{13}C NMR spectra of triazoles	S18
7.	Reference	S33

1. General considerations

All commercially available compounds and solvents were purchased and used as received, unless otherwise noted. Analytical thin-layer chromatography (TLC) was performed on precoated silica gel 60 F254 plates. Visualization on TLC was achieved by the use of UV light (254 nm). Flash chromatography was performed using silica gel (particle size 40-63 μm, 230-400 mesh). ^{1}H and ^{13}C NMR spectra were recorded on 400 MHz NMR (400 MHz for ^{1}H, 100 MHz for ^{13}C) and 500 MHz NMR (500 MHz for ^{1}H, 125 MHz for ^{13}C). Chemical shift values are given in parts per million relative to internal TMS (0.00 ppm for ^{1}H) or $CDCl_3$ (77.06 ppm for ^{13}C). The following abbreviations were used to describe peak splitting patterns when appropriate: br = broad, s = singlet, d = doublet, t = triplet, q = quartet, p = pentet, sext = sextet, sept = septet, m = multiplet. Coupling constants, *J*, were reported in hertz unit (Hz). Thermogravimetric analysis was examined by a PerkinElmer instrument under nitrogen atmosphere at a heating rate of 10 °C/min. Fourier transform infrared (FT-IR) spectra were recorded with a PerkinElmer

PXI spectrometer in KBr disk.

2. FT-IR spectra

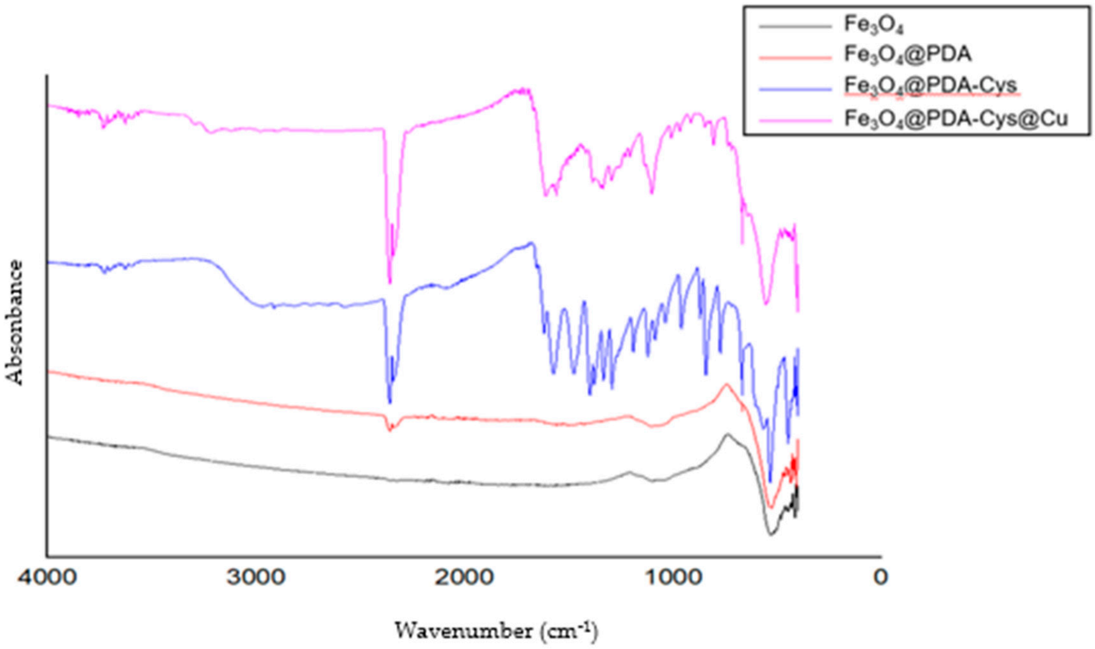

The infrared spectra corresponding to each platform in the area of 400–4000 cm^{-1} are recorded. Overall, the corresponding absorption spectra appear due to the decoration with organic substrates, such as PDA and cysteine, in Fe_3O_4@PDA and Fe_3O_4@PDA-Cys, respectively. The bands at 500 cm^{-1} in the Fe_3O_4 might be associated to the stretching vibration for the Fe–O bond. In the FTIR spectra of Fe_3O_4@PDA-Cys and Fe_3O_4@PDA-Cys@Cu platforms, the absorption bands between 1500–1600 cm^{-1} may be attributed to the C=C and C=O groups [1].

3. ^{1}H and ^{13}C NMR data of Sonogashira coupling products

1-methoxy-4-(phenylethynyl)benzene (3a); white solid. ^{1}H NMR (400 MHz, DMSO-*d6*) δ (ppm): 7.49-7.94(m, 4H), 7.43 – 7.39 (m, 3H), 7.00 – 6.98 (dd, *J* = 6.8, 2 Hz, 2H), 3.80 (s, 3H). ^{13}C NMR (100 MHz, DMSO) δ (ppm): 160.0, 133.4, 131.6, 129.2, 128.9, 123.1, 114.9, 114.7, 89.9, 88.4, 55.7.

1-methyl-2-(phenylethynyl)benzene (3b); ^{1}H NMR (400 MHz, $CDCl_3$) δ (ppm): 7.93 – 7.90 (m, 3H), 7.66 – 7.46 (m, *6*H), 2.89 (s, 3H). ^{13}C NMR (100 MHz, $CDCl_3$) δ (ppm): 140.5, 132.3, 131.9, 129.9, 128.8, 128.7, 128.6, 126.1, 124.0. 123.5, 94.0, 89.0.

1-fluoro-3-(phenylethynyl)benzene (3c); ^{1}H NMR (400 MHz, $CDCl_3$) δ (ppm): 7.50 – 7.48 (m, 2H), 7.26 – 7.23 (m, 4H), 7.20 – 7.12 (m, 2H), 6.96 – 6.90 (tdd, *J* = 8.4, 1.2, 0.8, 1H). ^{13}C NMR (100 MHz, $CDCl_3$) δ (ppm): 163.8, 161.4, 131.9, 130.1 (d, *J* = 8.6 Hz), 128.8, 128.6, 127.7 (d, *J*=3 Hz), 125.5 (d, *J* = 9.4 Hz), 123.0, 118.6 (d, *J* = 19.6 Hz), 115.8 (d, *J* = 21 Hz), 90.6, 88.4 (d, *J* = 3.4 Hz).

1-chloro-4-(phenylethynyl)benzene (3d); ^{1}H NMR (400 MHz, $CDCl_3$) δ (ppm): 7.57 – 7.56 (m, 2H), 7.51 – 7.49 (m, 2H), 7.39 – 7.35 (m, 5H). ^{13}C NMR (100 MHz, $CDCl_3$) δ (ppm): 134.3, 132.8, 131.6, 129.2, 128.7, 128.4, 122.9. 121.8.

3-(phenylethynyl)benzonitrile (3e); ^{1}H NMR (400 Hz, $CDCl_3$) δ (ppm): 7.69 – 7.68 (m, 1H), 7.64 (dt, *J* = 8.0, 1.2 Hz, 1H), 7.52 – 7.48 (m, 3H), 7.36 – 7.29 (m, 3H). ^{13}C NMR (100 MHz, $CDCl_3$) δ (ppm): 135.6, 134.8, 131.8, 131.4, 129.3, 129.1, 128.6, 124.9, 122.4, 118.1, 112.9, 91.9, 87.1.

(3-(phenylethynyl)phenyl)methanol (3f); ^{1}H NMR (400 MHz, $CDCl_3$) δ (ppm): 8.11 (d, *J* = 9.2 Hz, 2H), 7.47 – 7.44 (m, 3H), 7.40 – 7.37 (m, 1H), 7.29 – 7.25 (m, 5H), 4.61 (s, 2H), 1.18 (s, 1H). ^{13}C NMR (100 MHz, $CDCl_3$) δ (ppm): 141.1, 131.6, 130.8, 130.0, 128.6, 128.4, 128.3, 126.8, 123.5, 123.2, 89.5, 89.2, 64.9.

3-(p-tolylethynyl)aniline (3g); ^{1}H NMR (400 MHz, $CDCl_3$) δ (ppm): 7.40 (d, *J* = 8.0 Hz, 2H), 7.22 (d, *J* = 7.6 Hz, 2H), 7.06 – 7.02 (m, 1H), 6.72 – 6.71 (m, 1H), 6.66 (dt, *J* = 7.6, 1.2 Hz, 1H), 6.59 (ddd, *J* = 8.0, 2.0, 1.2 Hz, 1H). ^{13}C NMR (100 MHz, $CDCl_3$) δ (ppm): 149.2, 138.7, 131.6, 129.8, 129.7, 123.1, 120.0, 119.2, 116.6, 114.9, 90.2, 90.1, 21.5.

1-fluoro-3-(p-tolylethynyl)benzene (3h); white solid. ^{1}H NMR (400 MHz, $CDCl_3$) δ (ppm): 7.43 – 7.40 (m, 2H), 7.30 – 7.27 (m, 2H), 7.22 – 7.19 (m, 1H), 7.16 – 7.14 (m, 2H), 7.03 – 7.00 (m, 1H) 2.36 (s, 3H). ^{13}C NMR (100 MHz, $CDCl_3$) δ (ppm): 163.7, 161.2, 138.8, 129.9 (d, *J* = 8.7 Hz), 129.2, 127.4 (d, *J* = 3.1 Hz), 125.4 (d, *J* = 9.4 Hz), 119.7, 118.4 (d, *J* = 22.5 Hz), 115.5 (d, *J* = 21.1 Hz), 90.5, 87.5 (d, *J* = 3.4 Hz), 21.5.

4-((3-aminophenyl)ethynyl)benzonitrile (3i); yellow solid. ^{1}H NMR (400 MHz, $CDCl_3$) δ (ppm): 7.66 – 7.57 (m, 4H), 7.46 (dd, *J* = 6.8, 2.0 Hz, 1H), 7.26 (s, 1H), 7.21 – 7.13 (m, 1H), 6.96 – 6.93 (m, 1H), 6.89 – 6.85 (m, 1H), 6.70 (ddd, *J* = 8.0, 2.4, 0.8 Hz, 1H). ^{13}C NMR (100 MHz, $CDCl_3$) δ (ppm): 146.4, 133.2, 132.1, 132.0, 129.9, 129.7, 129.5, 122.9, 122.2, 118.6, 117.8, 116.1, 114.1, 111.4.

(3-((4-methoxyphenyl)ethynyl)phenyl)methanol (3j); yellow solid. ^{1}H NMR (400 MHz, $CDCl_3$) δ (ppm): 7.53 – 7.52 (m, 1H), 7.48 – 7.43 (m, 3H), 7.35 – 7.26 (m, 3H), 4.69 (s, 2H), 3.83 (s, 3H). ^{13}C NMR (100 MHz, $CDCl_3$) δ (ppm): 159.7, 141.0, 133.1, 130.6, 129.9, 128.6, 126.5, 123.9, 115.3, 114.0, 89.6, 87.9, 65.0, 55.3.

1-((4-bromophenyl)ethynyl)-2-methylbenzene (3k); white solid. ^{1}H NMR (400 MHz, DMSO) δ (ppm): 7.65 – 7.62 (m, 2H), 7.53 – 7.50 (m, 3H), 7.33 (dd, *J* = 4.8, 1.2 Hz, 2H), 7.26 – 7.22 (m, 1H), 2.46 (s, 3H). ^{13}C NMR (100 MHz, DMSO) δ (ppm): 140.2, 133.7, 132.3, 132.1, 130.2, 129.5, 126.5, 122.6, 122.2, 122.1, 92.6, 89.8, 20.7.

3-((3-aminophenyl)ethynyl)phenol (3l); yellow solid. ^{1}H NMR (400 MHz, DMSO) δ (ppm): 7.20 (t, *J* = 8.0 Hz, 1H), 7.12 (t, *J* = 8.0 Hz, 1H), 6.95 – 6.93 (m, 1H), 6.88 – 6.87 (m, 1H), 6.84 – 6.79 (m, 4H), 6.74 – 6.71 (m, 1H), 2.51 – 2.49 (m, 2H). ^{13}C NMR (100 MHz, DMSO) δ (ppm): 157.8, 130.3, 129.8, 123.7, 123.1, 122.6, 121.2, 119.6, 119.5, 118.2, 116.6, 116.4, 89.7, 88.9.

4. ¹H and ¹³C NMR spectra of substrates

1-methoxy-4-(phenylethynyl)benzene (3a)

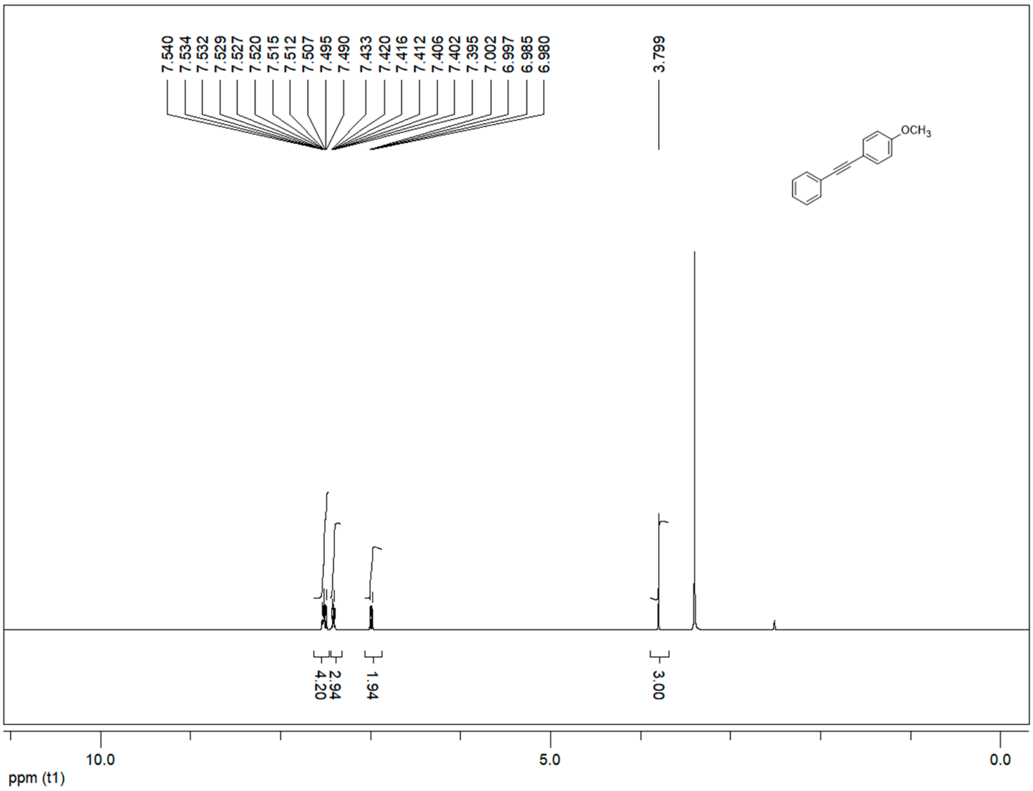

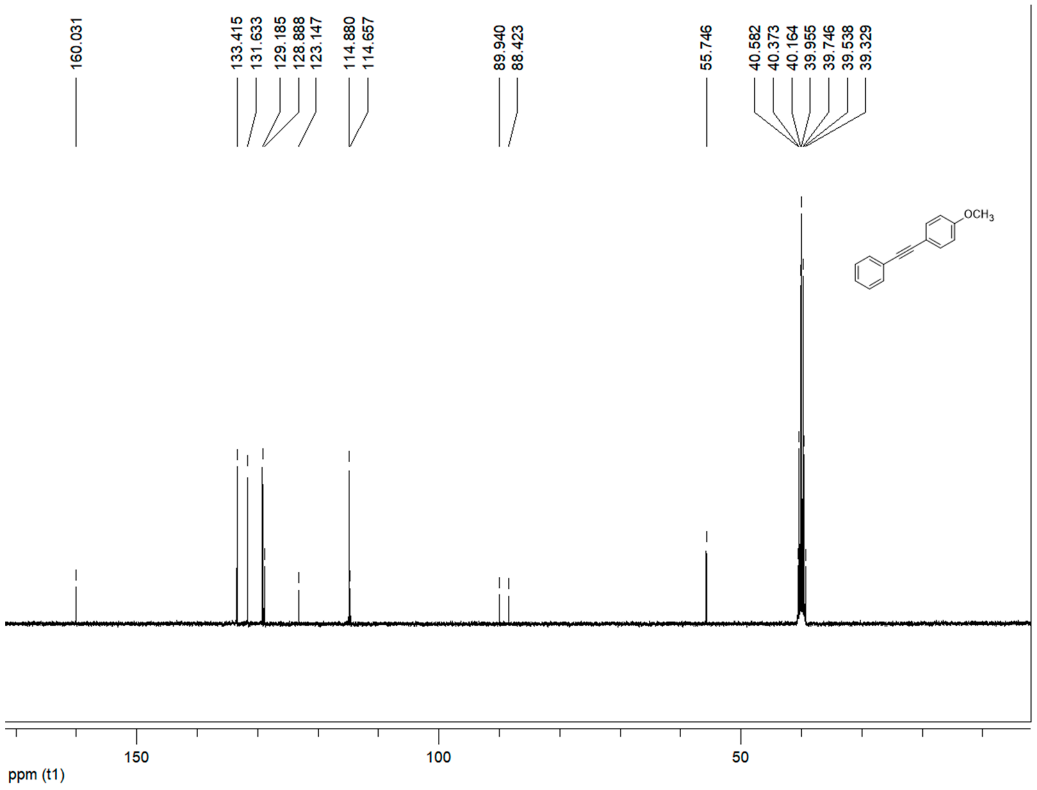

1-methyl-2-(phenylethynyl)benzene (3b)

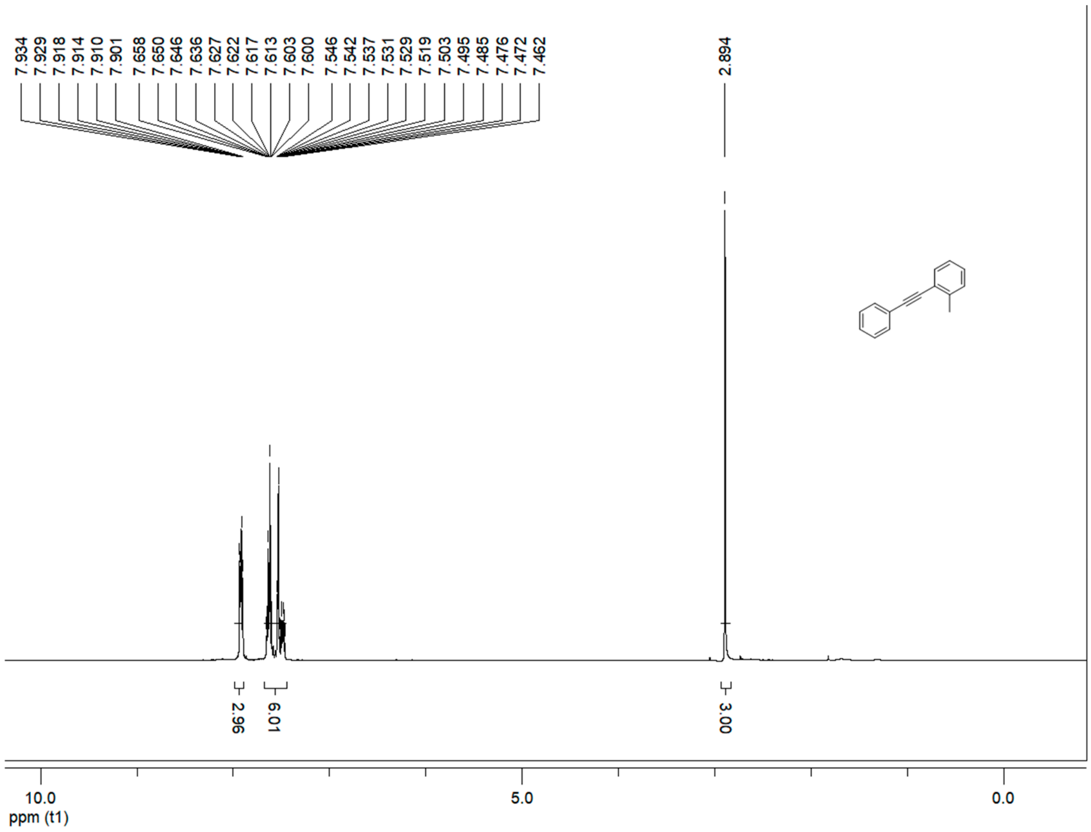

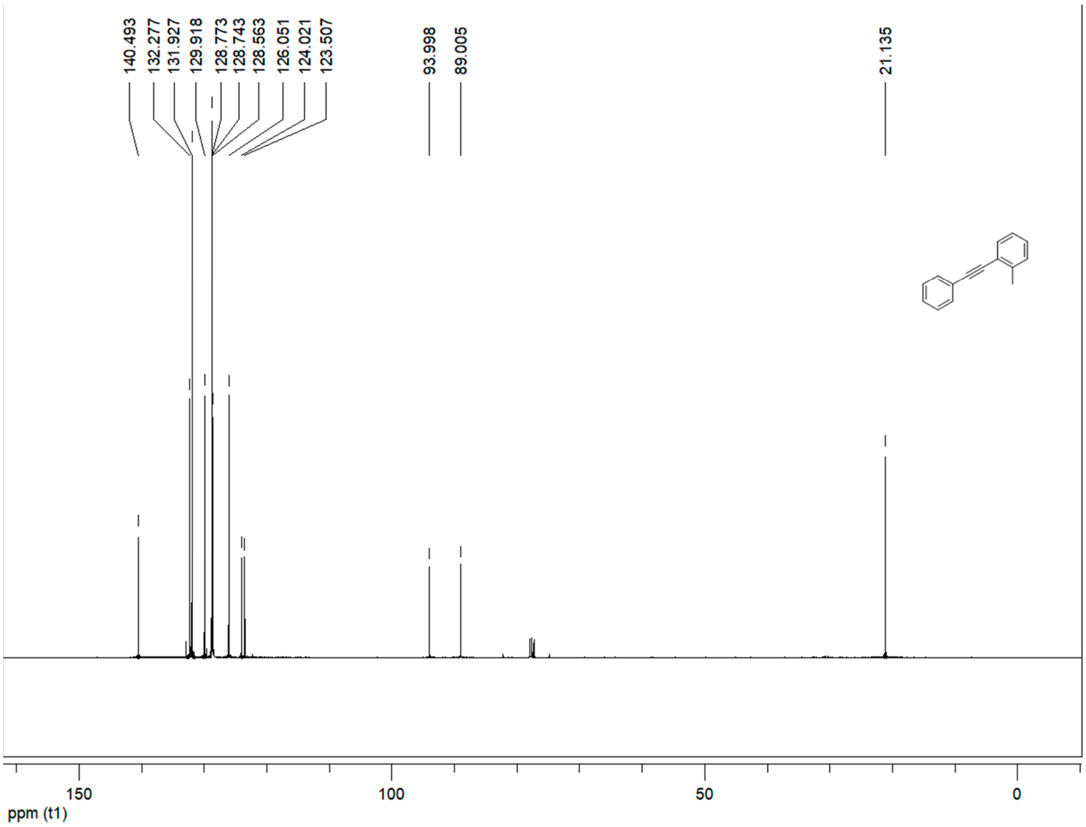

140.493
132.277
131.927
129.918
128.773
128.743
128.563
126.051
124.021
123.507
93.998
89.005
21.135
150
100
50
0
ppm (t1)

1-fluoro-3-(phenylethynyl)benzene (3c)

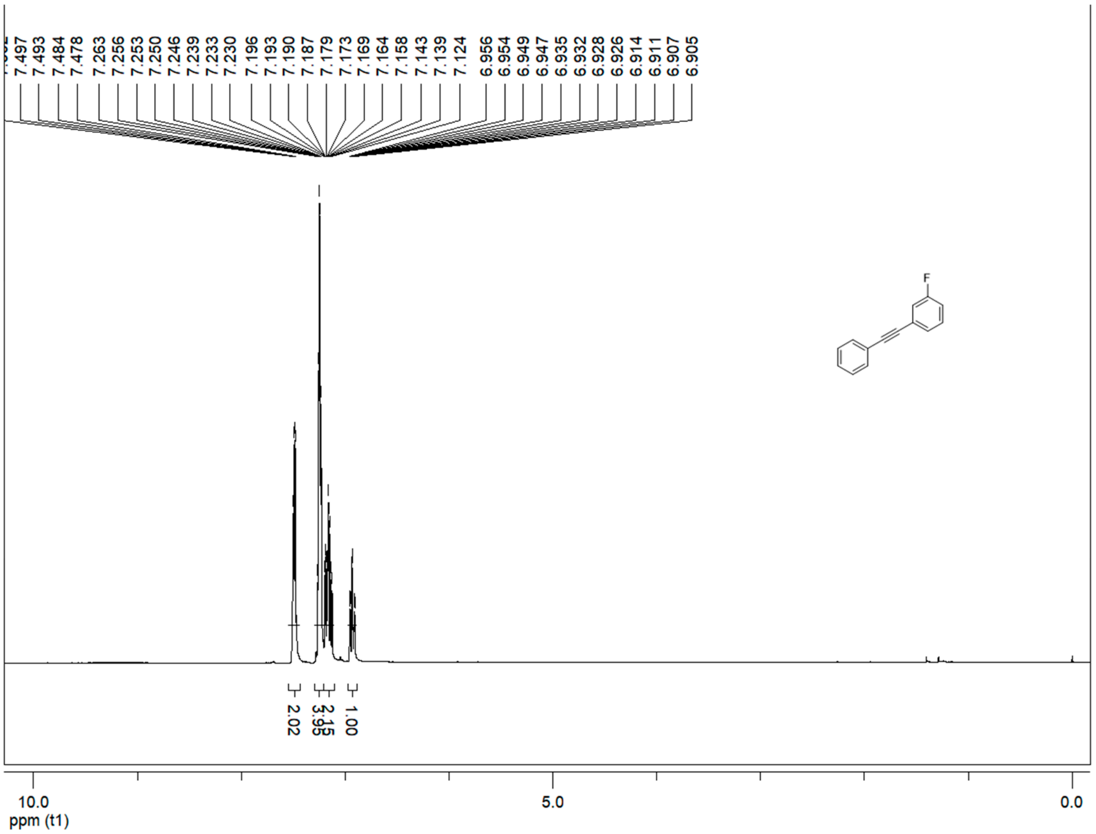

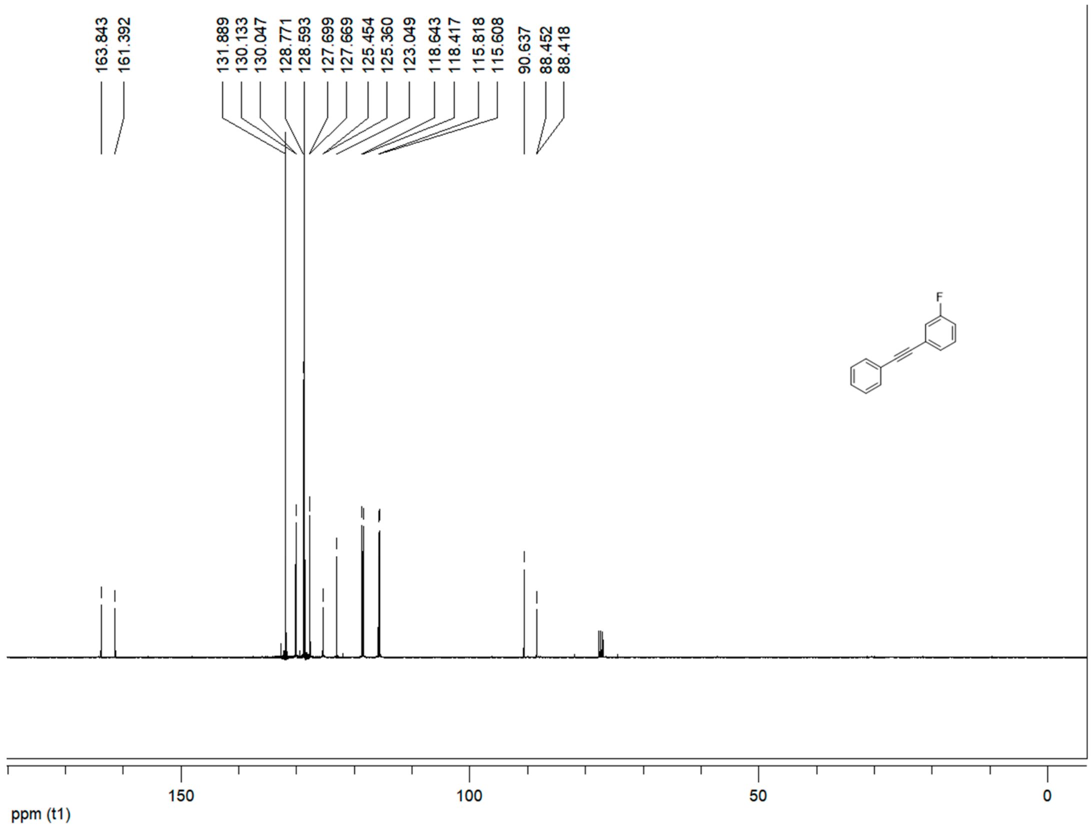

1-chloro-4-(phenylethynyl)benzene (3d)

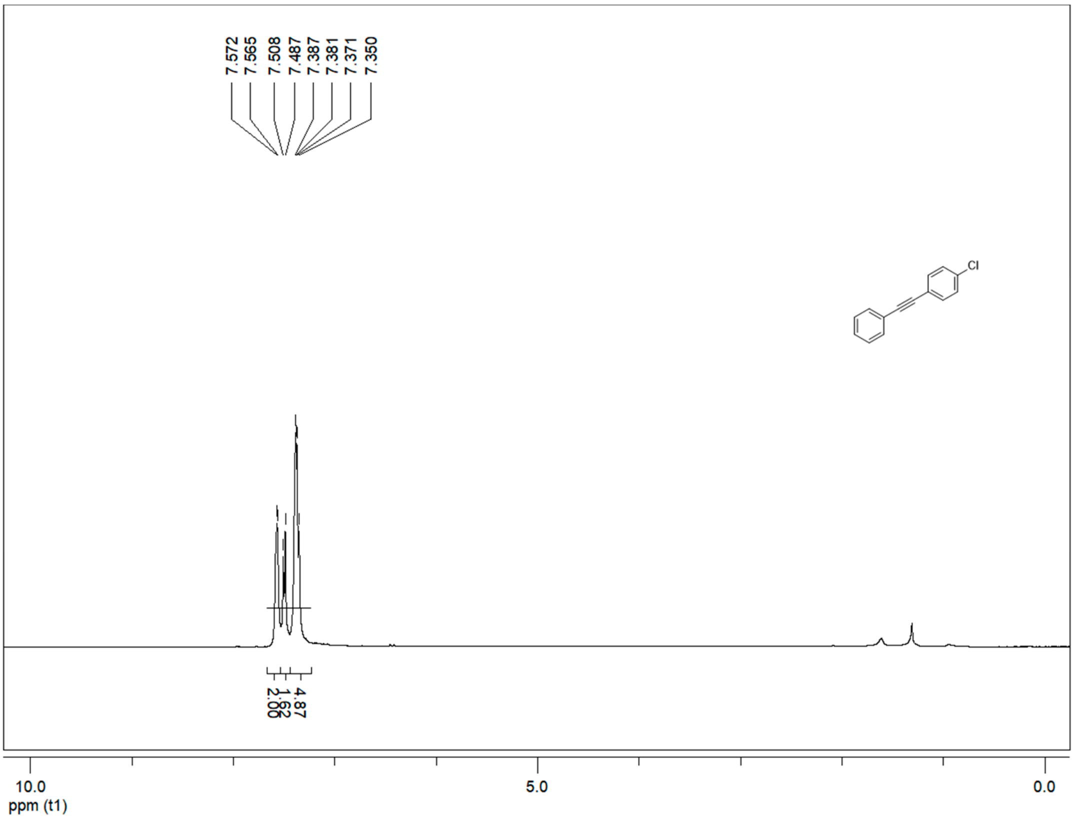

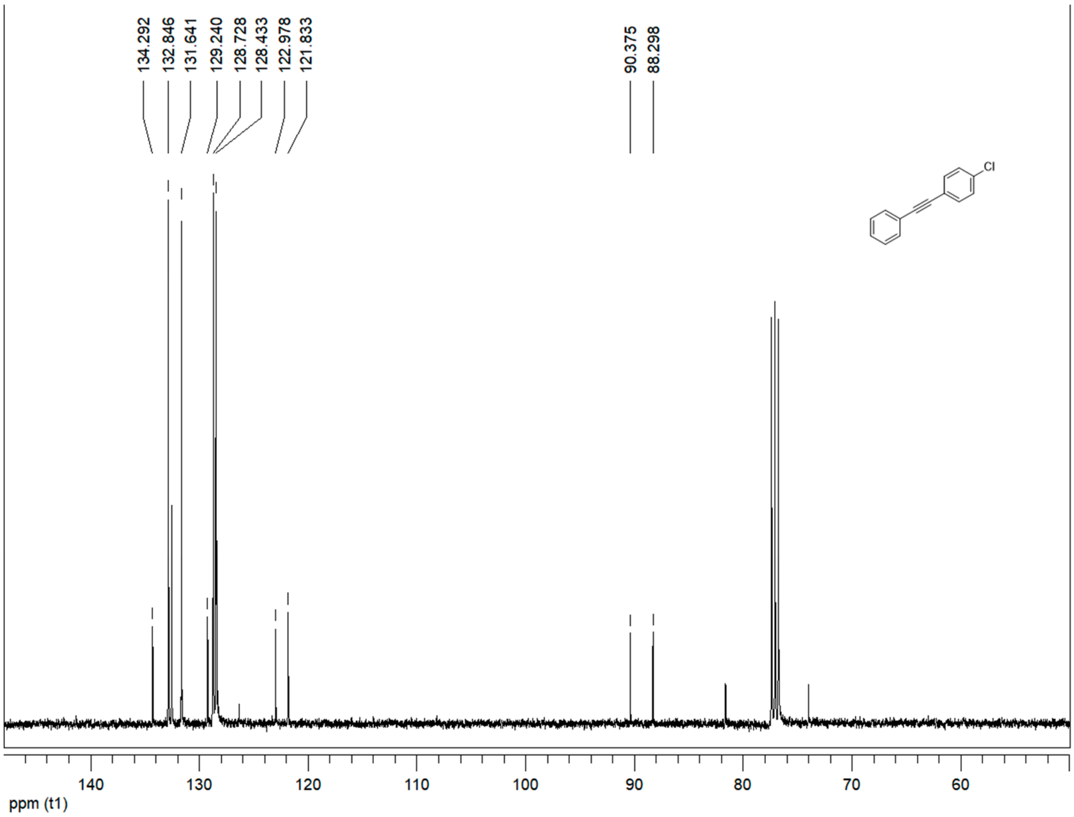

3-(phenylethynyl)benzonitrile (3e)

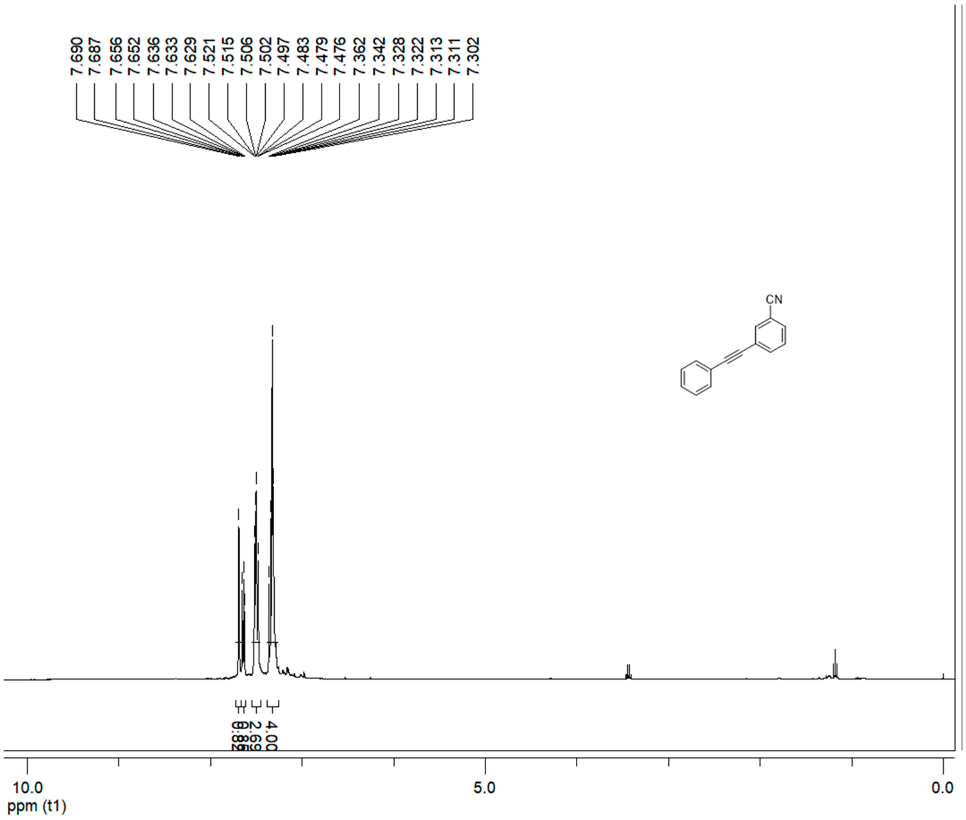

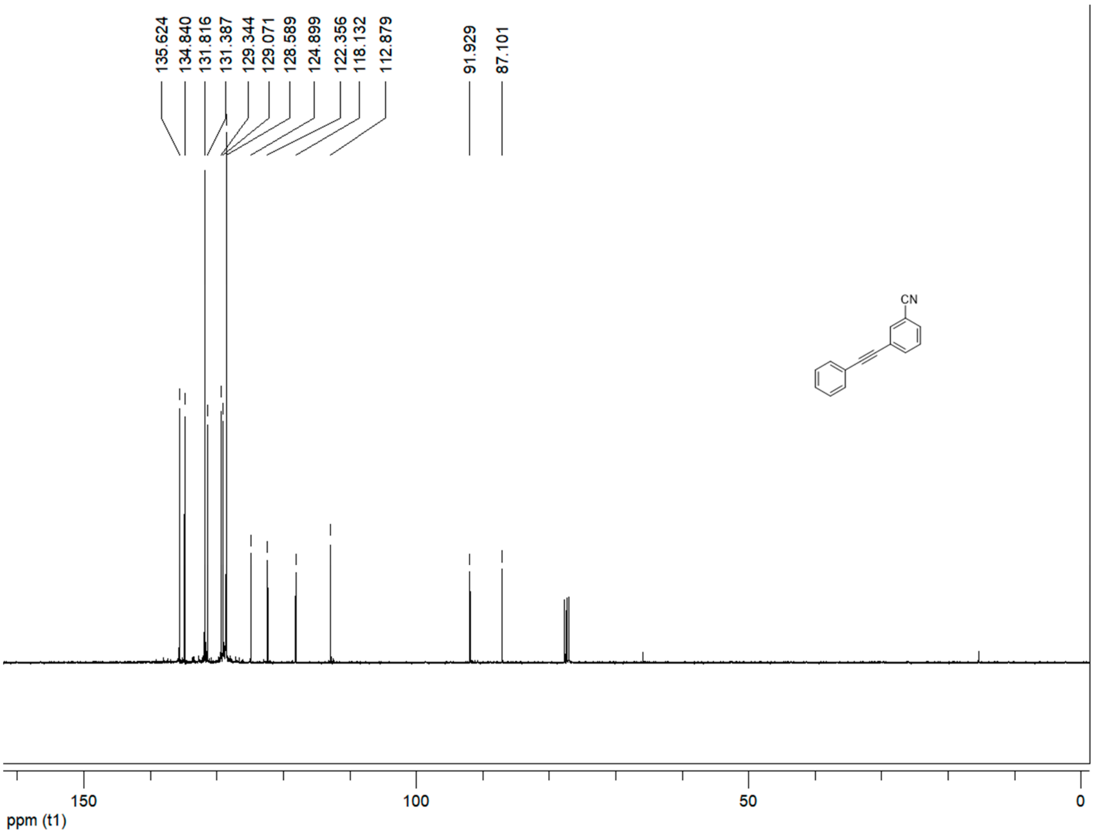

(3-(phenylethynyl)phenyl)methanol (3f)

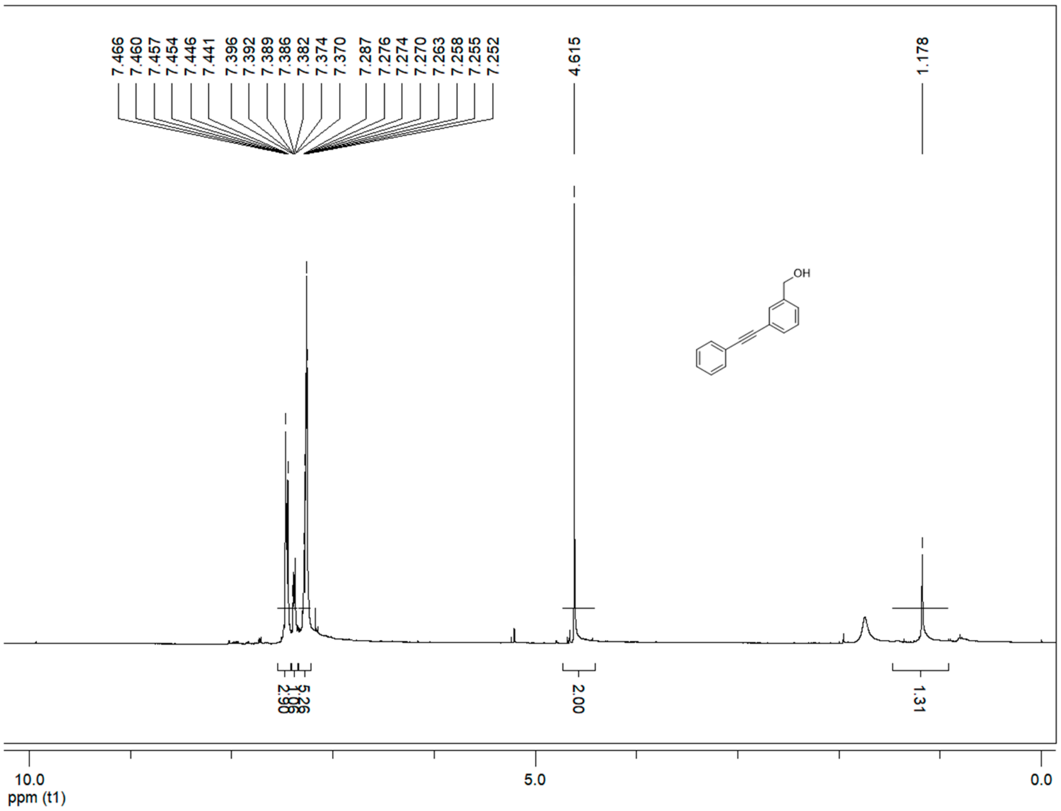

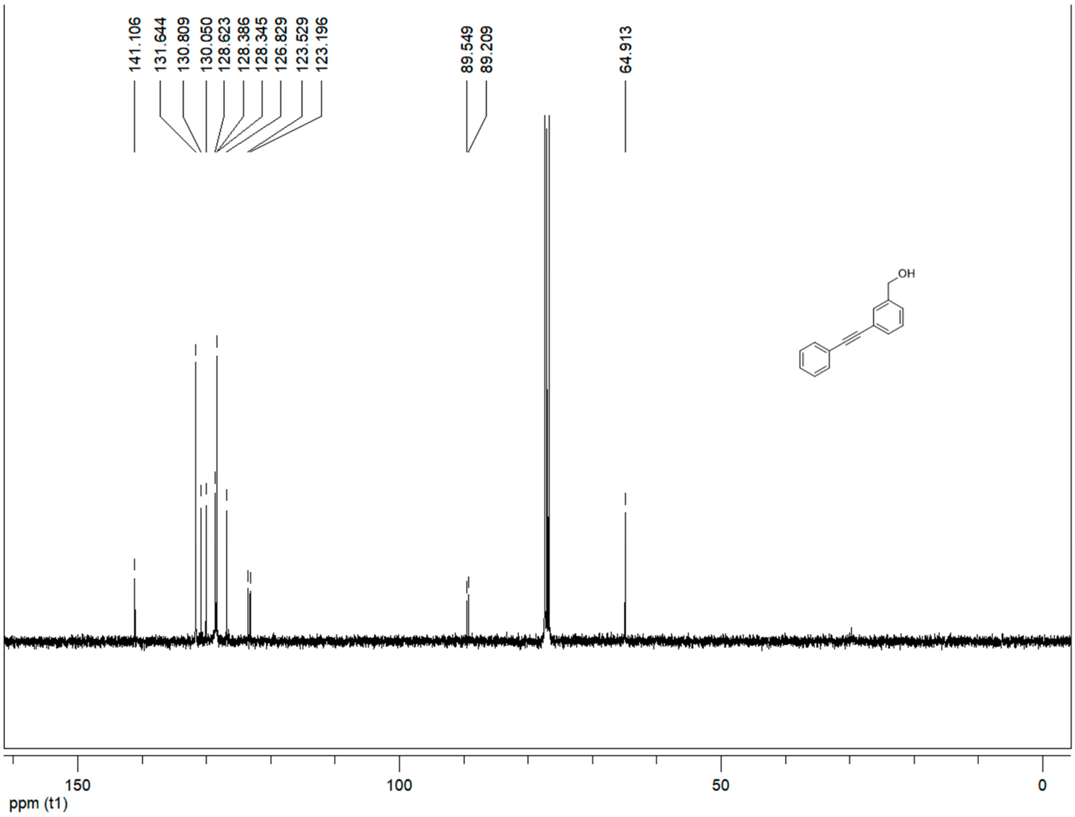

3-(p-tolylethynyl)aniline (3g)

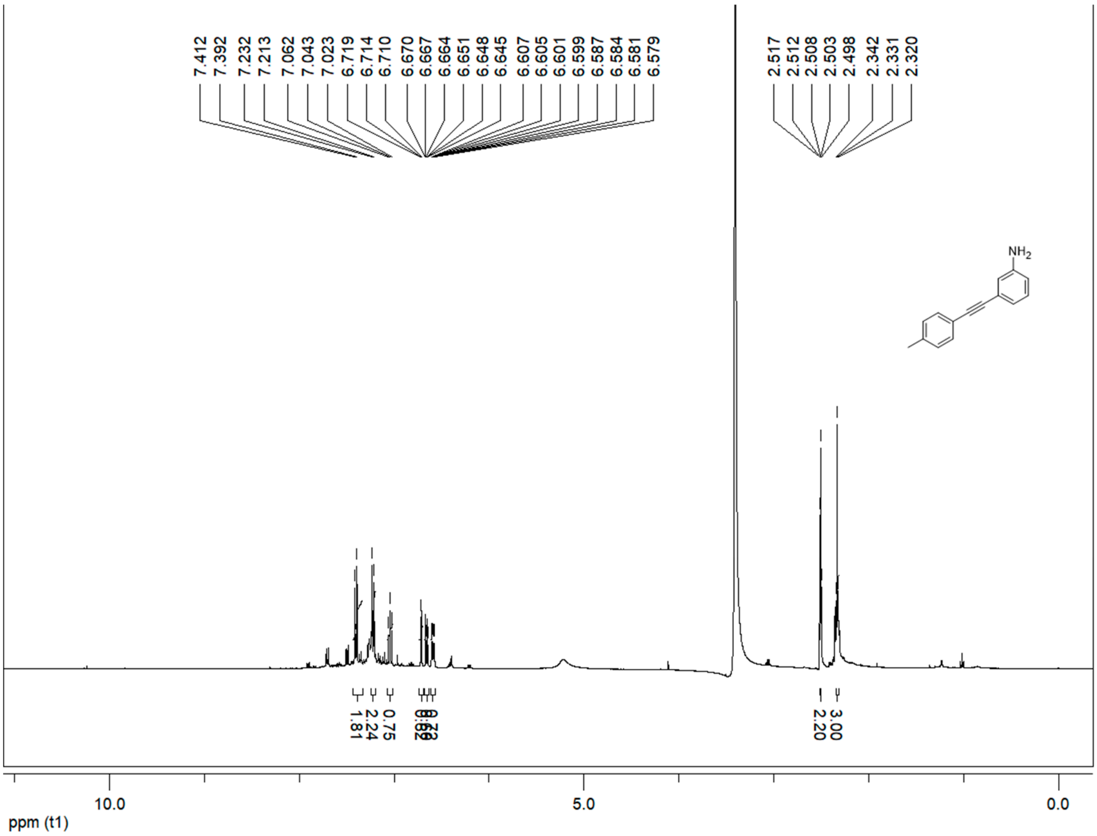

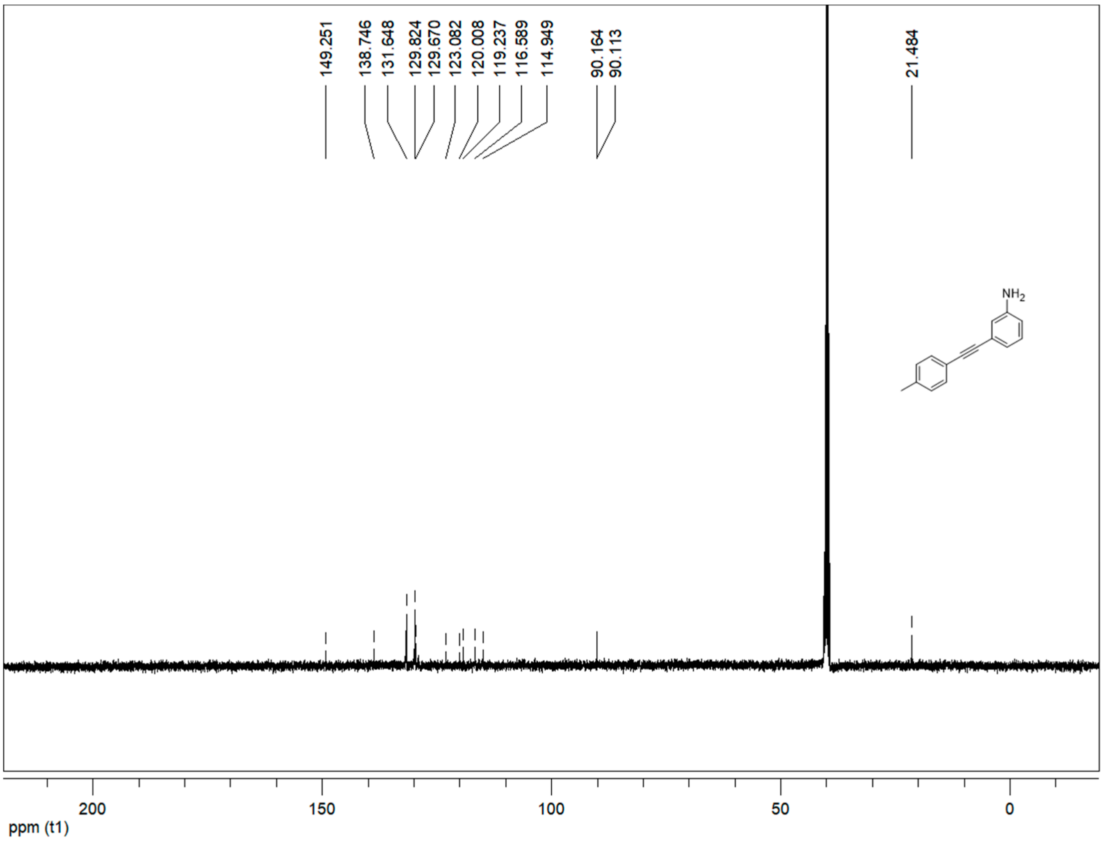

1-fluoro-3-(p-tolylethynyl)benzene (3h)

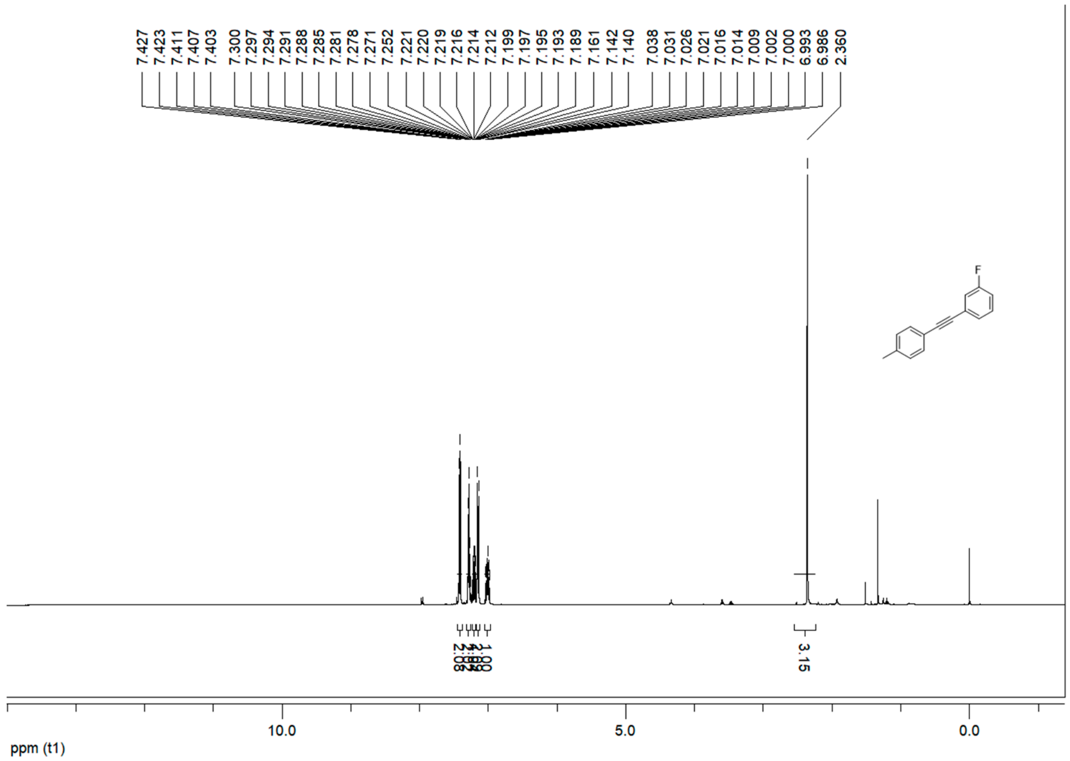

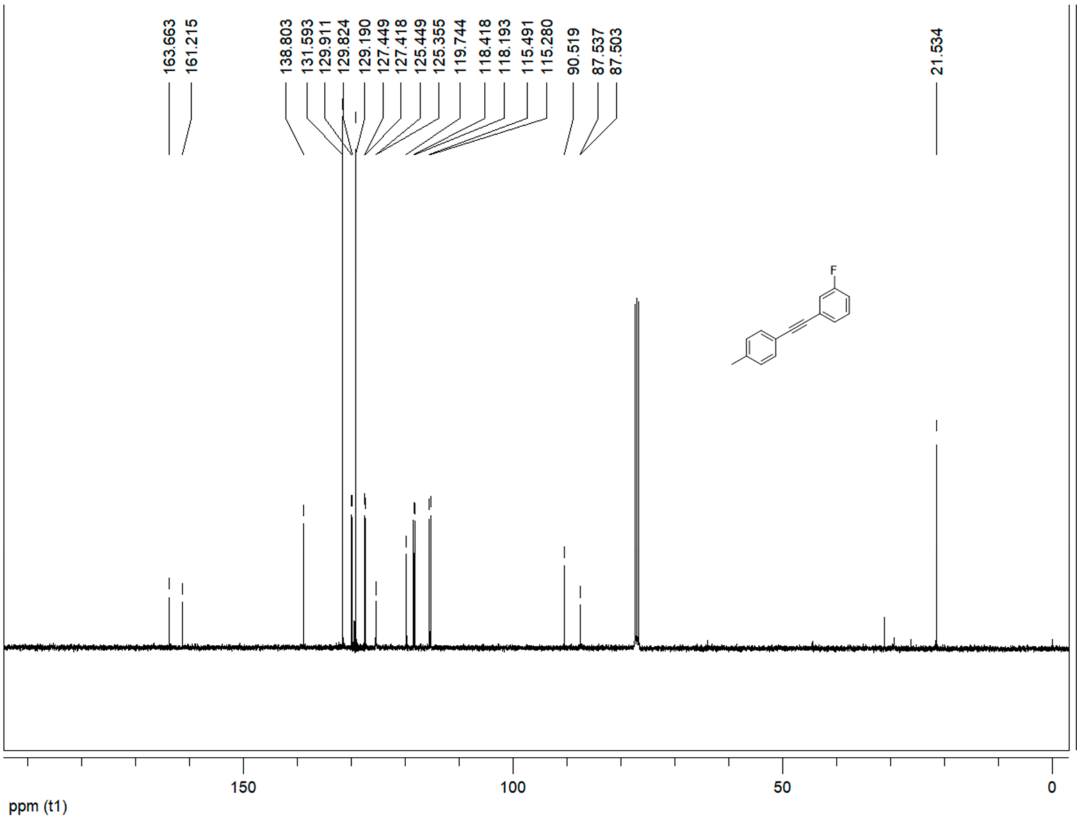

4-((3-aminophenyl)ethynyl)benzonitrile (3i)

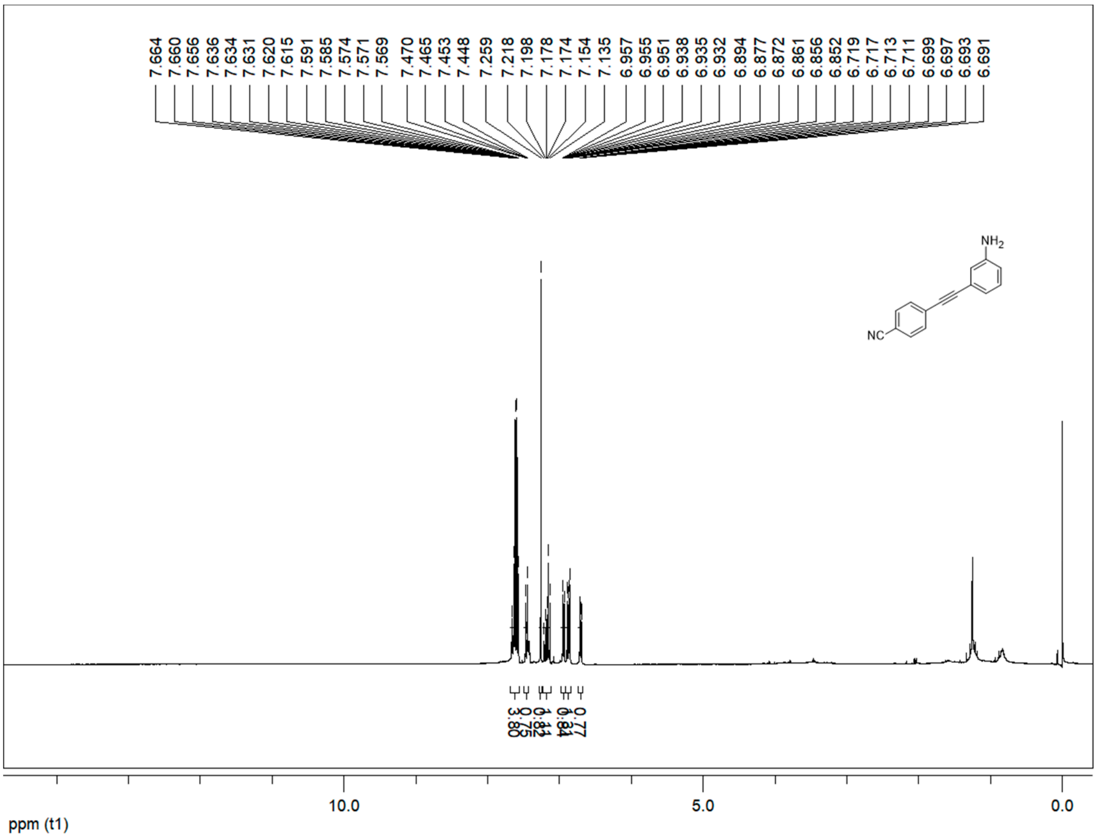

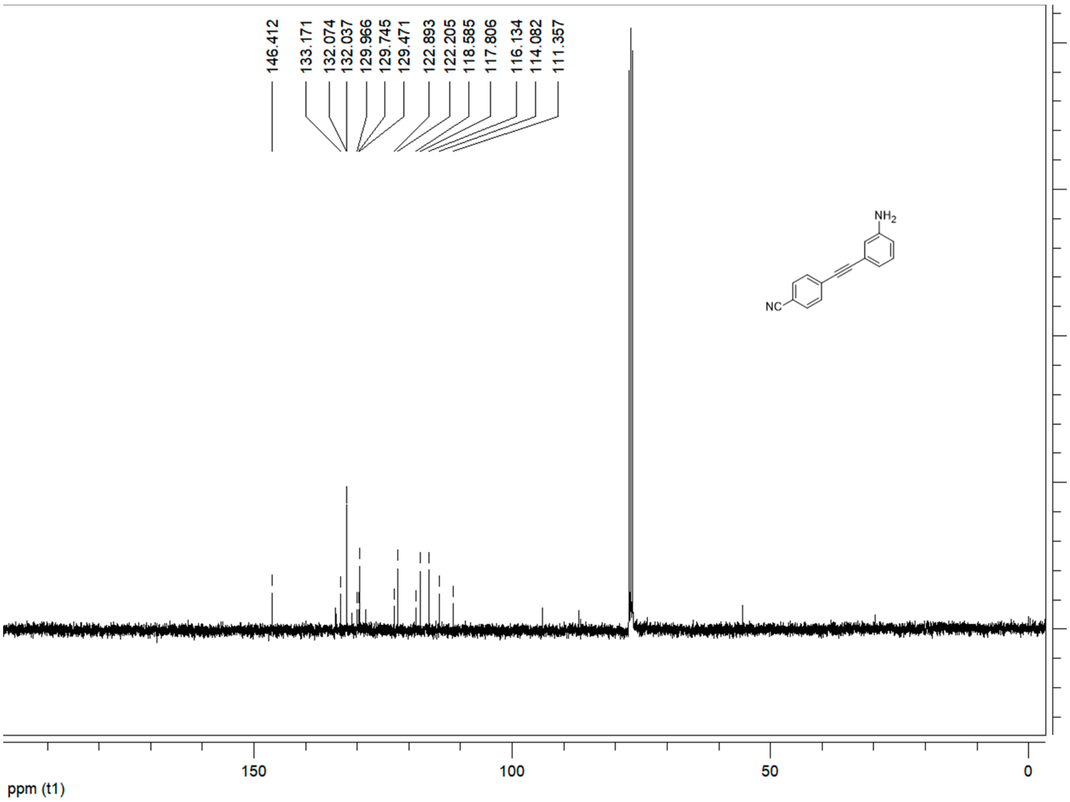

(3-((4-methoxyphenyl)ethynyl)phenyl)methanol (3j)

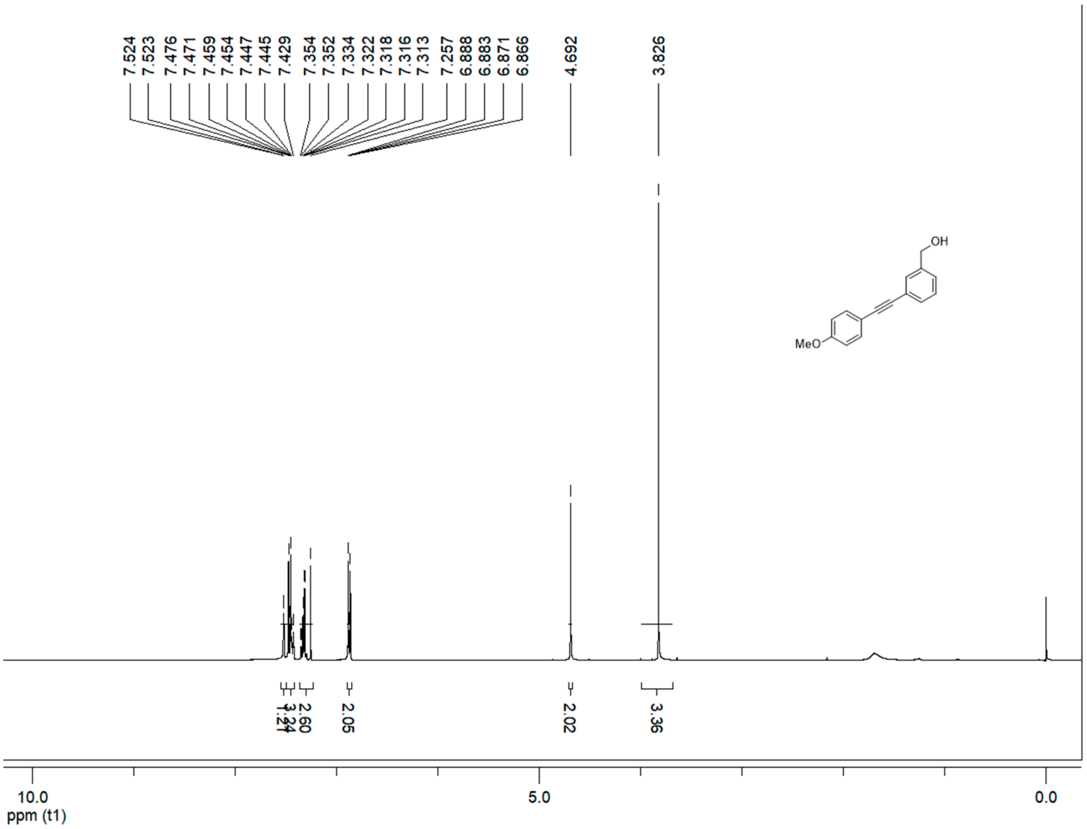

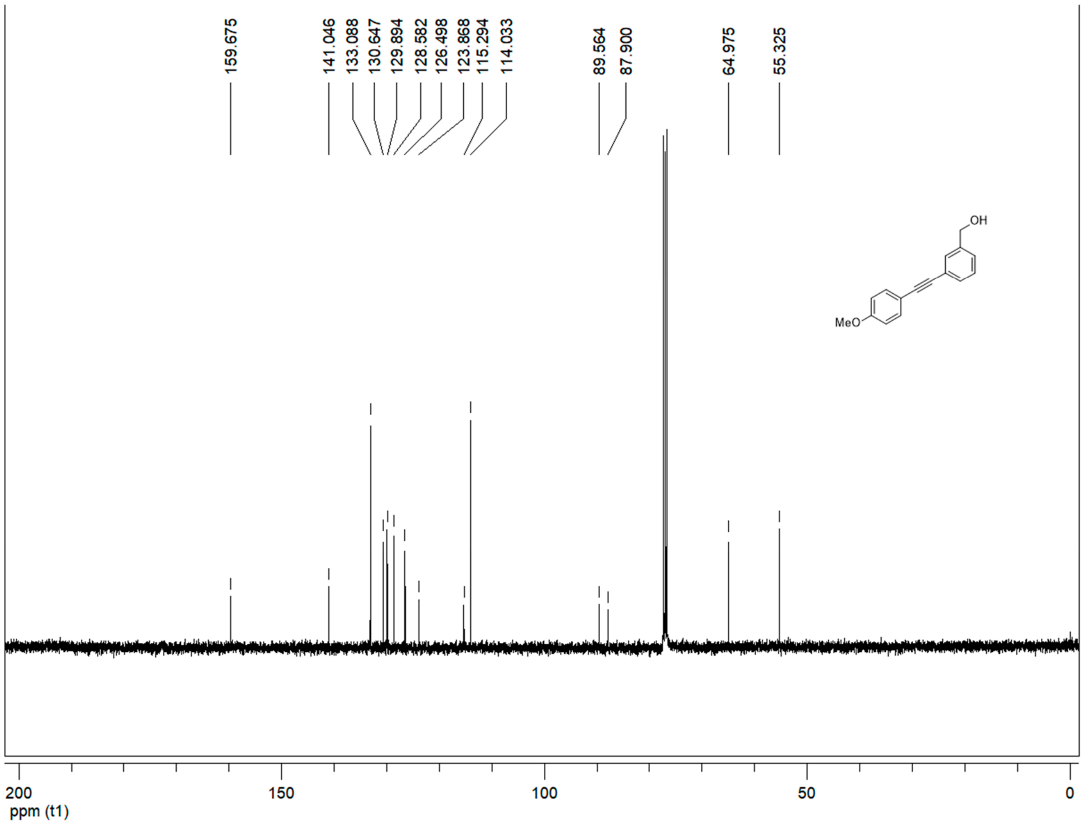

1-((4-bromophenyl)ethynyl)-2-methylbenzene (3k)

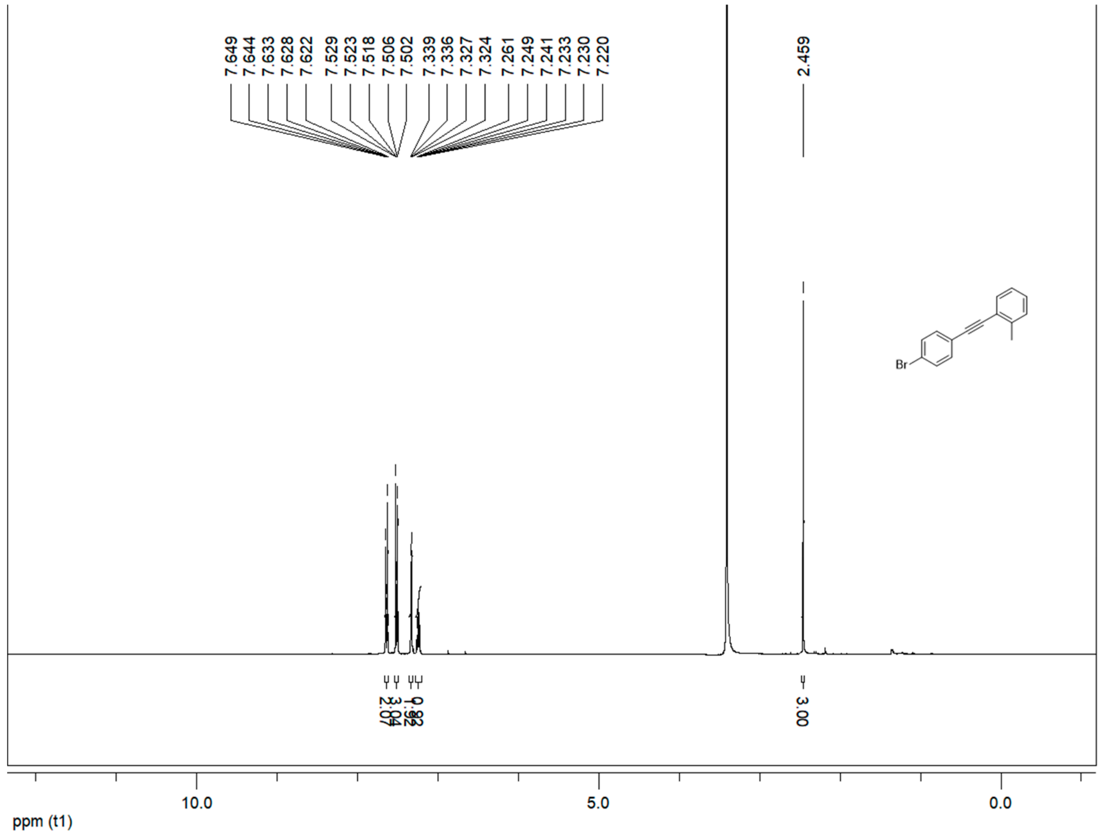

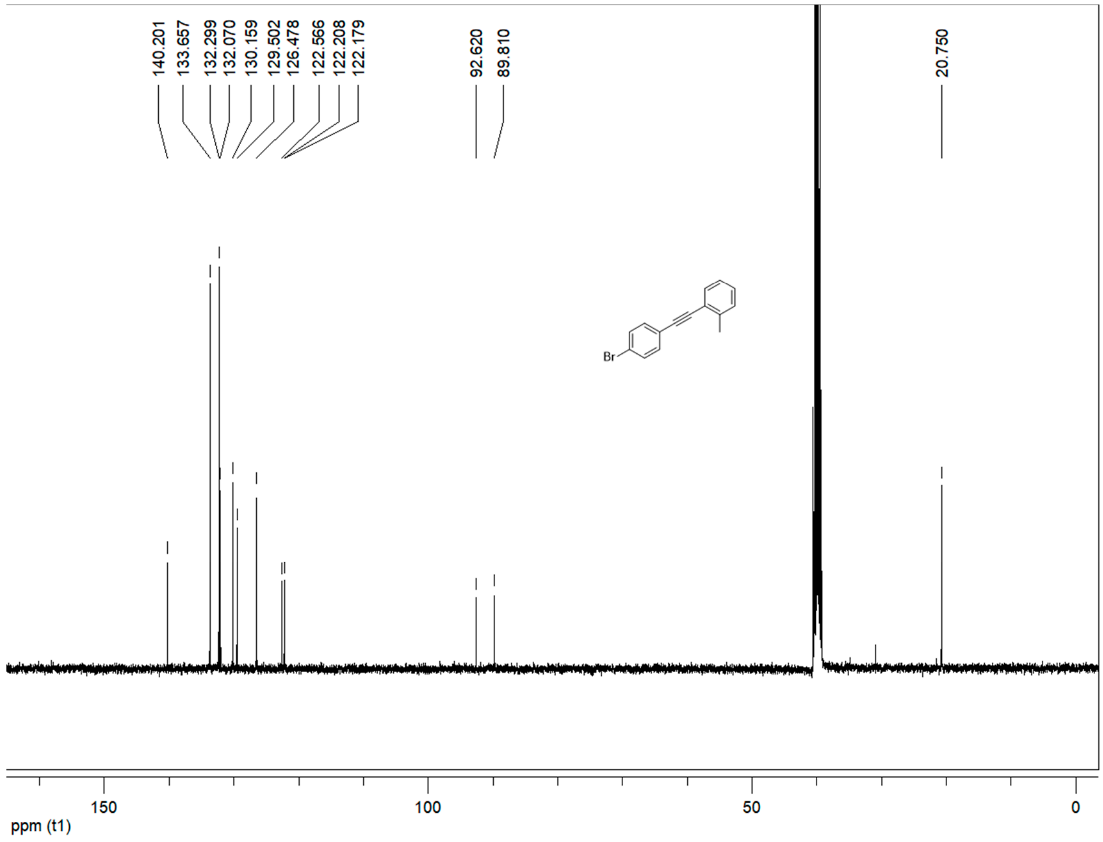

3-((3-aminophenyl)ethynyl)phenol (3l)

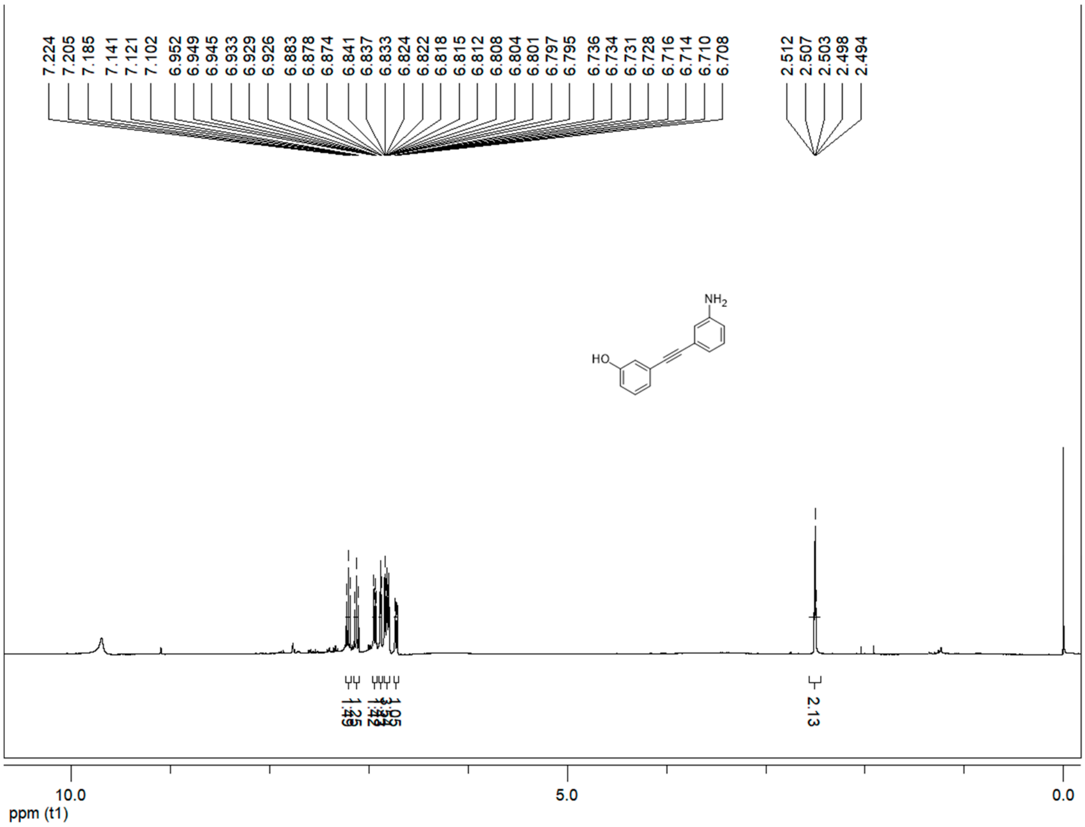

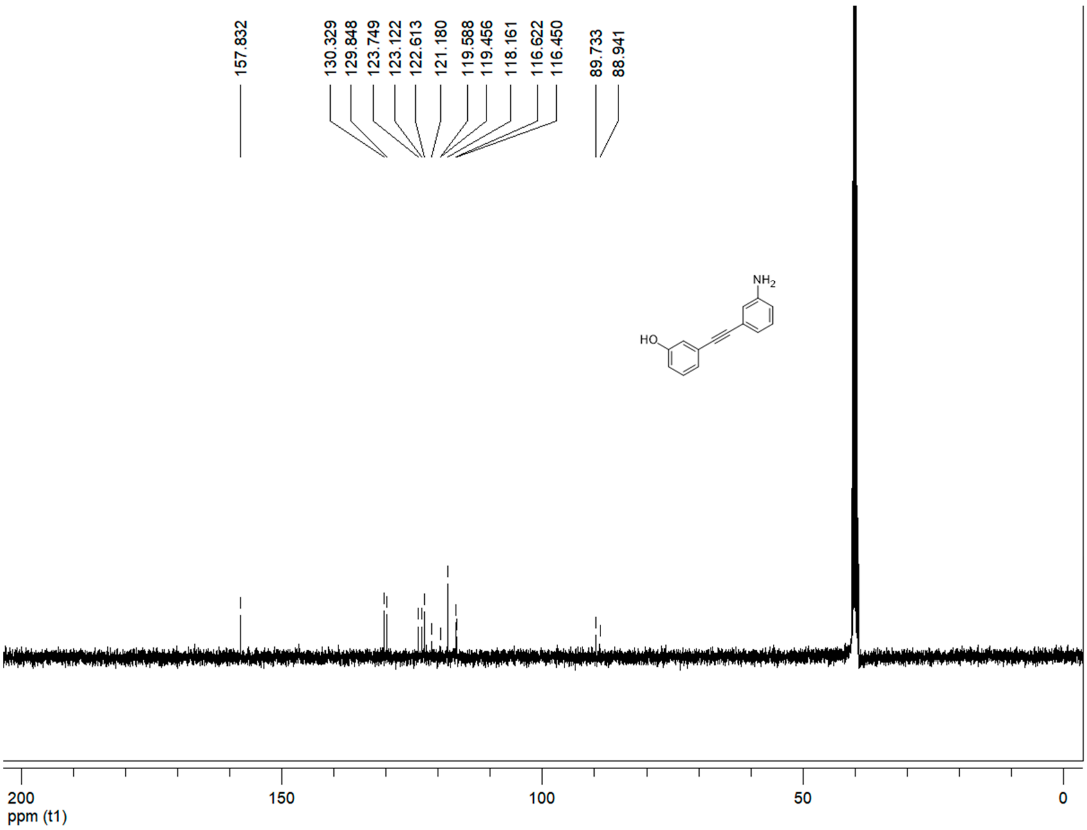

5. ^{1}H and ^{13}C NMR data of the triazoles

1-Benzyl-4-phenyl-1*H*-1,2,3-triazole (**4a**): White solid; mp 127-128 °C. ^{1}H NMR (400 MHz, $CDCl_3$) δ (ppm): 7.69-7.66(m, 2H), 7.56 (s, 1H), 7.29-7.22 (m, 5H), 7.21-7.16 (m, 3H), 5.41 (s, 2H). ^{13}C NMR (100 MHz, $CDCl_3$) δ (ppm): 148.2, 134.8, 130.6, 129.1, 128.8, 128.7, 128.2, 128.0, 125.7, 119.7, 54.1.

1-(4-Bromobenzyl)-4-phenyl-1*H*-1,2,3-triazole (**4b**): White solid; mp 152 °C. ^{1}H NMR (400 MHz, $CDCl_3$) δ (ppm): 7.71-7.69(m, 2H), 7.59 (s, 1H), 7.41 (dt, *J* = 8.4, 4.0 Hz, 2H), 7.32-7.28 (m, 2H), 7.25-7.21 (m, 1H), 7.07 (d, *J* = 8.0 Hz, 2H), 5.41 (s, 2H). ^{13}C NMR (100 MHz, $CDCl_3$) δ (ppm): 133.7, 132.3, 130.4, 129.6, 128.8, 125.7, 122.9, 119.5, 53.5.

1-(4-(tert-Butyl)benzyl)-4-phenyl-1*H*-1,2,3-triazole (4c): White solid; mp 164 °C. ^{1}H NMR (400 MHz, $CDCl_3$) δ (ppm): 7.72-7.69(m, 2H), 7.58 (s, 1H), 7.32-7.27 (m, 4H), 7.21 (m, 1H), 7.16 (dt, *J* = 8.0, 2.0 Hz, 2H), 5.44 (s, 2H), 1.23 (s, 9H). ^{13}C NMR (100 MHz, $CDCl_3$) δ (ppm): 151.9, 148.1, 131.7, 130.6, 128.8, 128.1, 127.9, 126.0, 125.7, 119.5, 118.0, 53.9, 34.6, 31.3.

1-(4-Fluorobenzyl)-4-phenyl-1*H*-1,2,3-triazole (4d): White solid; mp 138 °C. ^{1}H NMR (400 MHz, $CDCl_3$) δ (ppm): 7.72-7.69(m, 2H), 7.58 (s, 1H), 7.32-7.24 (m, 2H), 7.22-7.17 (m, 3H), 7.00-6.95 (m, 2H), 5.44 (s, 2H). ^{13}C NMR (100 MHz, $CDCl_3$) δ (ppm): 162.9 (d, $J_{C\text{-}F}$ = 246.6 Hz, $\underline{C}$F), 148.3, 130.6, 130.5, 130.4, 129.9 (d, $J_{C\text{-}F}$ = 8.3 Hz, 2C), 128.8, 128.2, 125.7, 119.4. 116.1 (d, $J_{C\text{-}F}$ = 21.7 Hz, 2C), 53.5.

1-(4-(Cyanobenzyl)-4-phenyl-1*H*-1,2,3-triazole (4e): white solid; mp 139°C. ^{1}H NMR (400 MHz, $CDCl_3$) δ (ppm): 7.82-7.79(m, 3H), 7.65-7.63 (m, 2H), 7.43-7.31 (m, 5H), 5.63 (s, 2H). ^{13}C NMR (100 MHz, $CDCl_3$) δ (ppm): 148.50, 140.05, 132.88, 130.19, 128.96, 128.48, 128.42, 125.73, 120.09, 120.05, 118.29, 112.57, 112.55, 53.39

1-(2,4-Dichlorobenzyl)-4-phenyl-1*H*-1,2,3-triazole (4f): White solid; mp 120-121 °C. ^{1}H NMR (400 MHz, $CDCl_3$) δ (ppm): 7.84-7.82(m, 2H), 7.79 (s, 1H), 7.47 (d, *J* = 2.4 Hz, 1H), 7.44-7.41 (m, 2H), 7.35 (m, 1H), 7.28-7.25 (m, 1H), 7.16 (d, *J* = 8.4 Hz, 1H), 5.67 (s, 2H). ^{13}C NMR (100 MHz, $CDCl_3$) δ (ppm): 148.3, 135.6, 134.1, 131.2, 131.1, 130.3, 129.8, 128.9, 128.3, 127.9, 125.7, 119.8, 50.8.

1-Benzyl-4-(4-fluorophenyl)-1*H*-1,2,3-triazole (4g): White solid; mp 111-112 °C. ^{1}H NMR (400 MHz, $CDCl_3$) δ (ppm): 7.69-7.64(m, 2H), 7.55 (s, 1H), 7.32-7.00 (m, 5H), 6.99-6.96 (m, 2H), 5.45 (s, 2H). ^{13}C NMR (100 MHz, $CDCl_3$) δ (ppm): 162.7 (d, $J_{C\text{-}F}$ = 245.8 Hz, $\underline{C}$F), 161.4, 134.6, 129.2, 128.8, 127.5 (d, $J_{C\text{-}F}$ = 8 Hz, 2C), 126.8, 126.7, 115.8 (d, $J_{C\text{-}F}$ = 21.7 Hz, 2C), 54.3.

1-Benzyl-4-(4-butylphenyl)-1*H*-1,2,3-triazole (4h): White solid; mp 115-116 °C. ^{1}H NMR (400 MHz, $CDCl_3$) δ (ppm): 7.73 (d, *J* = 8.4 Hz, 2H), 7.65 (s, 1H), 7.41-7.40 (m, 3H), 7.39-7.38 (m, 2H), 7.33-7.23 (m, 2H), 5.58 (s, 2H), 2.64 (t, *J* = 8.0 Hz, 2H), 1.66-1.59 (m, 2H), 1.43-1.33 (m, 2H), 0.97-0.93 (m, 3H). ^{13}C NMR (100 MHz, $CDCl_3$) δ (ppm): 140.1, 134.8, 129.1, 128.9, 128.7, 128.0, 127.9, 125.6, 119.2, 54.2.

1-Benzyl-4-(4-methoxyphenyl)-1*H*-1,2,3-triazole (4i): White solid; mp 142 °C. ^{1}H NMR (400 MHz, $CDCl_3$) δ (ppm): 7.56 (s, 1H), 7.31-7.30 (m, 1H), 7.22-7.20 (m, 4H), 7.17-7.13 (m, 3H), 6.73 (m, 1H), 5.39 (s, 2H), 3.68 (s, 3H). ^{13}C NMR (100 MHz, $CDCl_3$) δ (ppm): 160.0, 148.0, 134.7, 131.9, 129.9, 129.1, 128.7, 128.0, 119.9, 118.1, 114.2, 110.7, 55.3, 54.1.

1-Benzyl-4-(4-nitrophenyl)-1*H*-1,2,3-triazole (4j): Yellow solid; mp 167 °C. ^{1}H NMR (400 MHz, $CDCl_3$) δ (ppm): 8.26 (dt, *J* = 8.8, 1.8 Hz, 2H), 7.98 (dt, *J* = 8.8, 1.8 Hz, 2H), 7.84 (s, 1H), 7.43-7.34 (m, 5H), 5.63 (s, 2H). ^{13}C NMR (100 MHz, $CDCl_3$) δ (ppm): 147.3, 146.0, 136.8, 134.2, 129.3, 129.0, 128.2, 126.1, 124.3, 121.0, 54.5.

3-(1-Benzyl-1*H*-1,2,3-triazol-4-yl)phenol (4k): White solid; mp 142 °C. ^{1}H NMR (400 MHz, $CDCl_3$) δ (ppm): 7.64 (s, 1H), 7.31-7.17 (m, 5H), 6.77-6.76 (m, 2H), 6.75-6.74 (m, 1H), 5.49 (s, 2H), 4.69 (s, O$\underline{H}$). ^{13}C NMR (100 MHz, $CDCl_3$) δ (ppm): 156.6, 147.7, 134.2, 130.9, 130.2, 129.2, 128.9, 128.2, 120.1,

117.9, 115.9, 112.9, 54.5

1-(4-Fluorobenzyl)-4-(4-nitrophenyl)-1*H*-1,2,3-triazole (4l): Yellow solid; mp 167 °C. ^{1}H NMR (400 MHz, $CDCl_3$) δ (ppm): 8.16 (d, J = 8.8 Hz, 2H), 7.88 (d, J = 8.8 Hz, 2H), 7.27-7.24 (m, 2H), 7.04-6.99 (m, 2H), 5.51 (s, 2H). ^{13}C NMR (100 MHz, $CDCl_3$) δ (ppm): 163.0 (d, J_{C-F} = 247.3 Hz, $\underline{C}$F), 147.3, 146.1, 136.7, 130.1 (d, J_{C-F} = 8.3 Hz, 2C), 126.1, 124.3, 120.9, 116.3 (d, J_{C-F} = 21.7 Hz, 2C), 53.7. HRMS (EI): m/z $C_{15}H_{11}FN_4O_2$ calcd for $[M]^+$ 298.0866, found 298.0874.

1-(2,4-Dichlorobenzyl)-4-(4-fluorophenyl)-1*H*-1,2,3-triazole (4m): White solid; mp 116 °C. ^{1}H NMR (400 MHz, $CDCl_3$) δ (ppm): 7.73-7.67 (m, 2H), 7.65 (s, 1H), 7.38 (d, J = 2.0 Hz, 1H), 7.19-7.16 (m, 1H), 7.10-7.08 (m, 1H), 7.04-6.99 (m, 2H), 5.58 (s, 2H). ^{13}C NMR (100 MHz, $CDCl_3$) δ (ppm): 162.7 (d, J_{C-F} = 246.1 Hz, $\underline{C}$F), 147.4, 135.6, 134.1, 131.1 (d, J_{C-F} = 5.8 Hz, 2C), 129.8, 128.0, 127.5, 127.4, 126.5, 119.5, 115.8 (d, J_{C-F} = 21.7 Hz, 2C), 50.8. HRMS (EI): m/z $C_{15}H_{10}Cl_2FN_3$ calcd for $[M]^+$ 321.0235, found 321.0232.

1-(4-(tert-Butyl)benzyl)-4-(m-tolyl)-1*H*-1,2,3-triazole (4n): White solid; mp 115 °C. ^{1}H NMR (400 MHz, $CDCl_3$) δ (ppm): 7.71-7.69 (m, 2H), 7.60 (d, J = 7.6 Hz, 1H), 7.42-7.40 (m, 2H), 7.31-7.25 (m, 3H), 7.15-7.13 (m, 1H), 5.52 (s, 2H), 2.39 (s, 3H), 1.34 (s, 9H). ^{13}C NMR (100 MHz, $CDCl_3$) δ (ppm): 151.8, 148.2, 138.4, 131.8, 130.5, 128.9, 128.7, 127.9, 126.4, 126.0, 122.8, 119.6, 53.8, 34.6, 31.3, 21.4. HRMS (EI): m/z $C_{20}H_{23}N_3$ calcd for $[M]^+$ 305.1892, found 305.1888.

6. Copies of ^{1}H and ^{13}C NMR spectra of triazoles

1-Benzyl-4-phenyl-1*H*-1,2,3-triazole (**4a**)

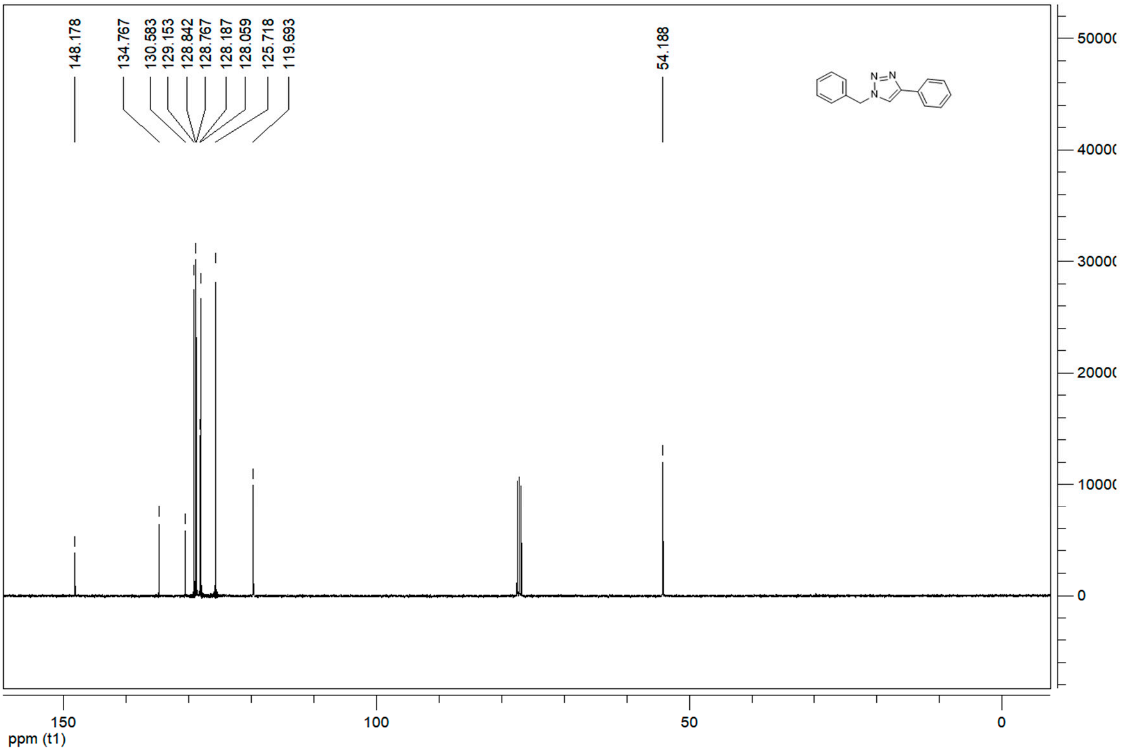

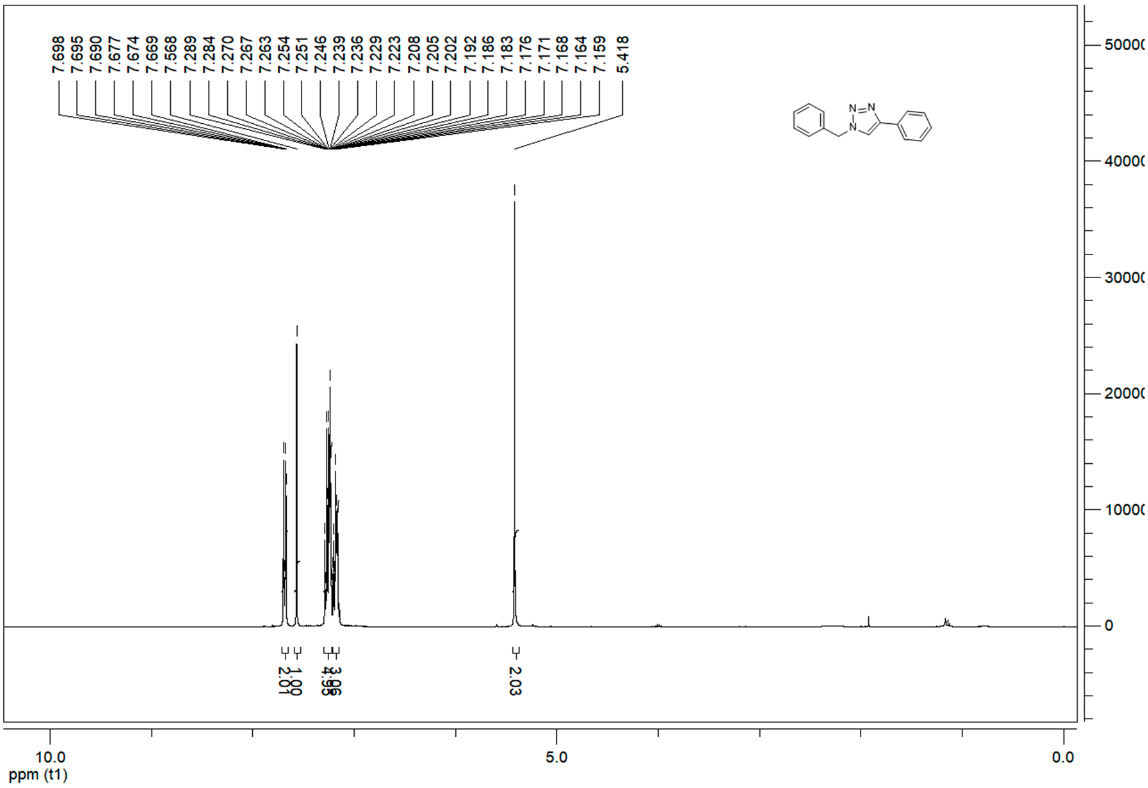

1-(4-Bromobenzyl)-4-phenyl-1*H*-1,2,3-triazole (**4b**)

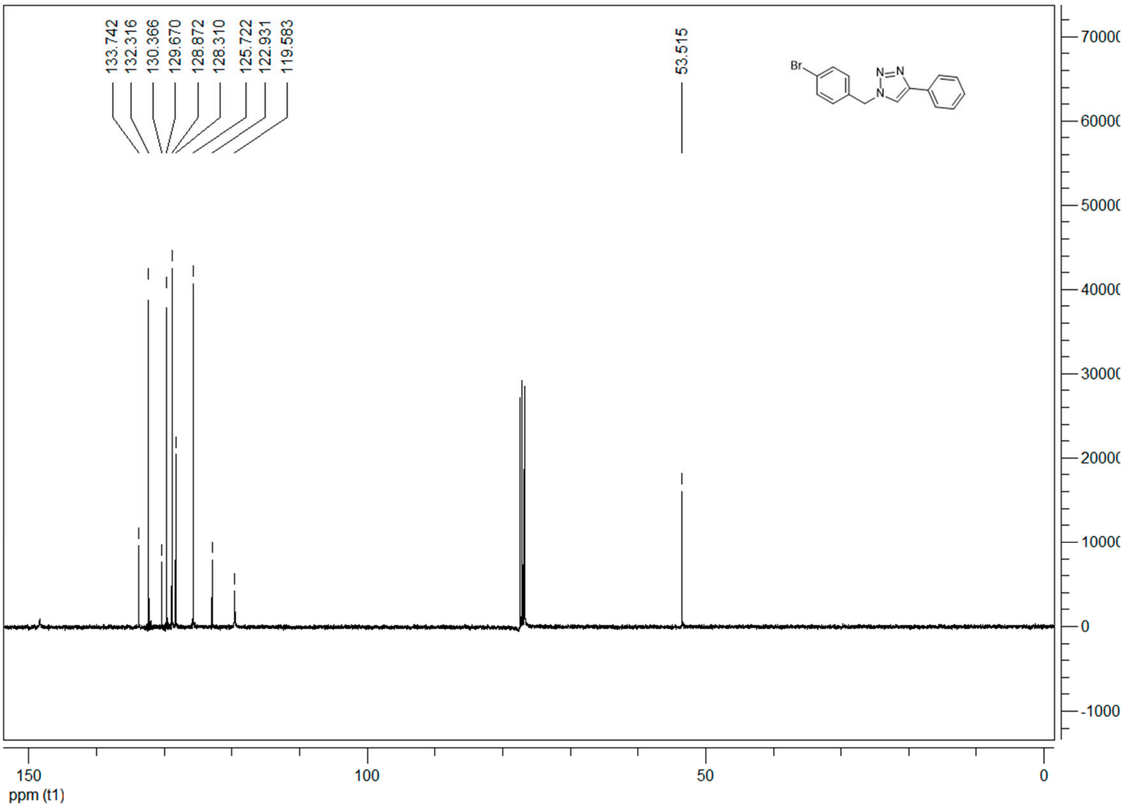

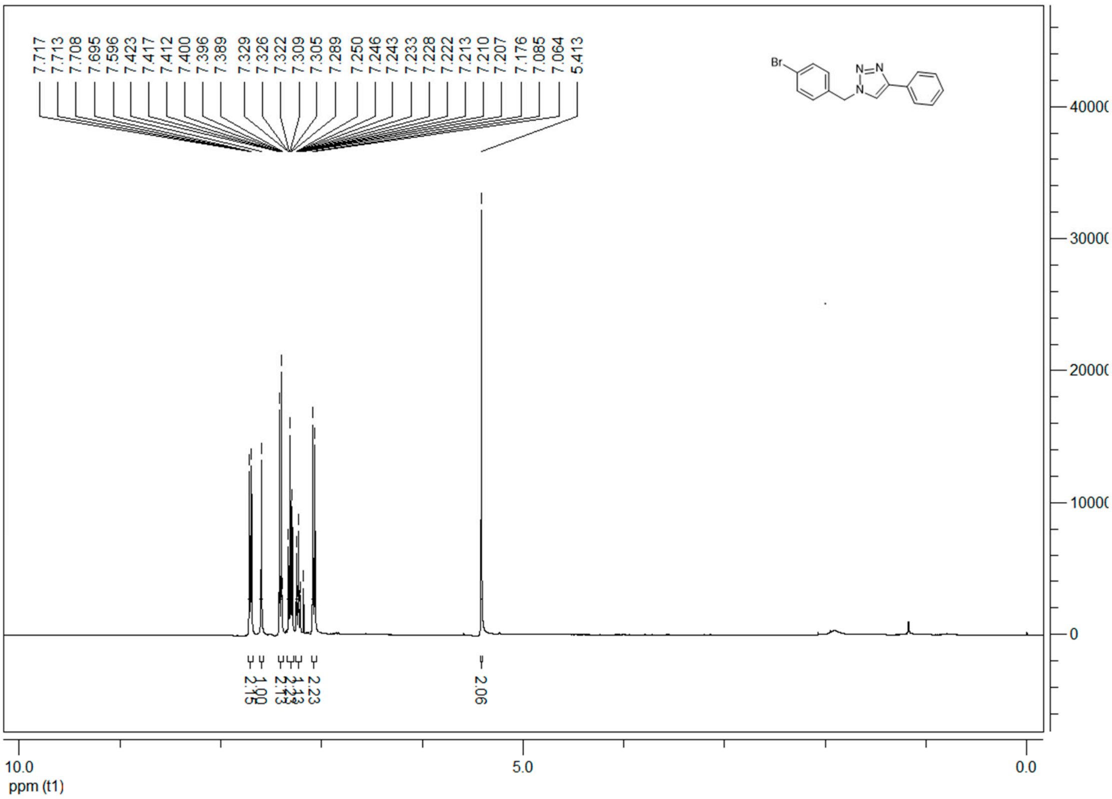

1-(4-(^{t}-Butyl)benzyl)-4-phenyl-1*H*-1,2,3-triazole (**4c**)

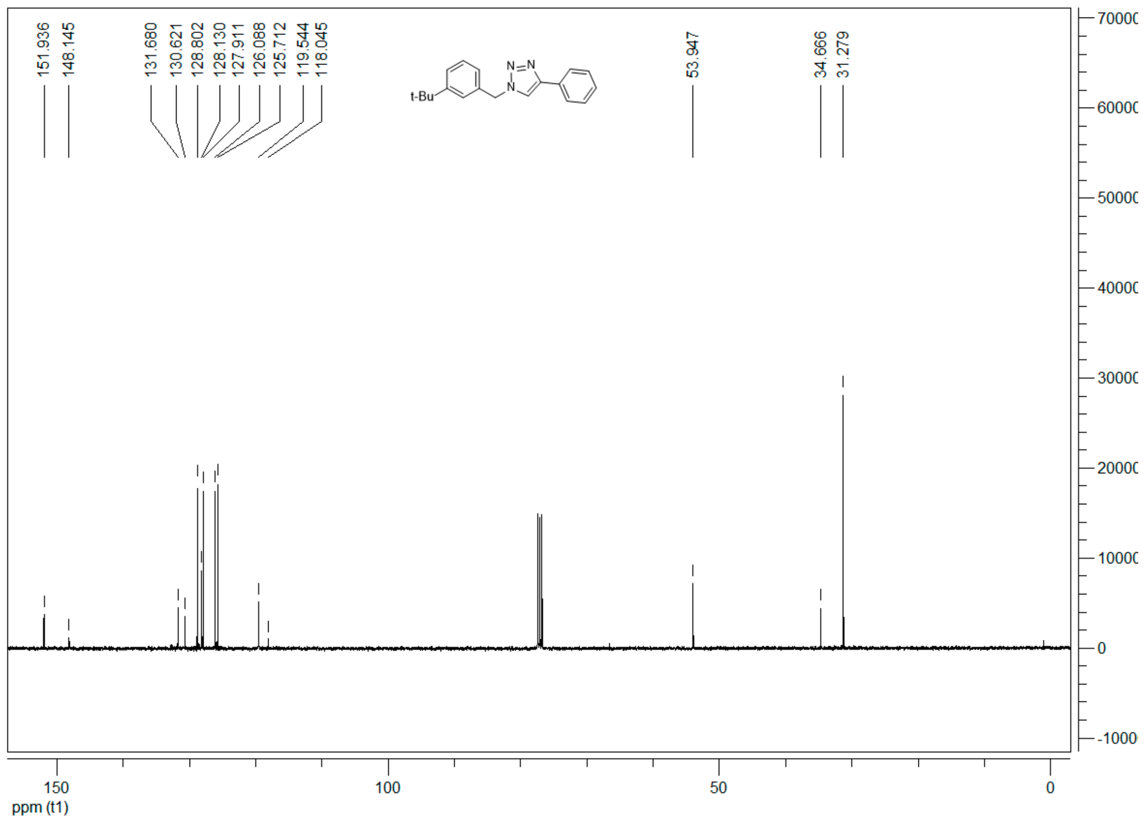

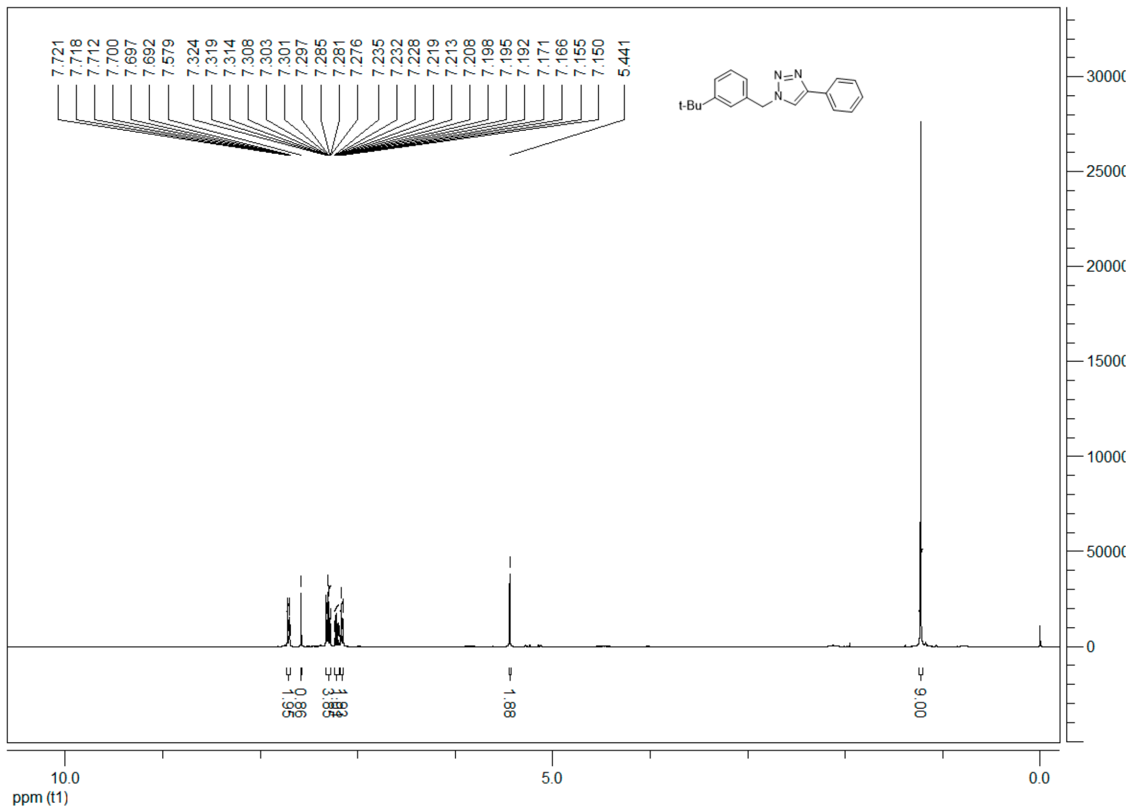

1-(4-Fluorobenzyl)-4-phenyl-1*H*-1,2,3-triazole (**4d**)

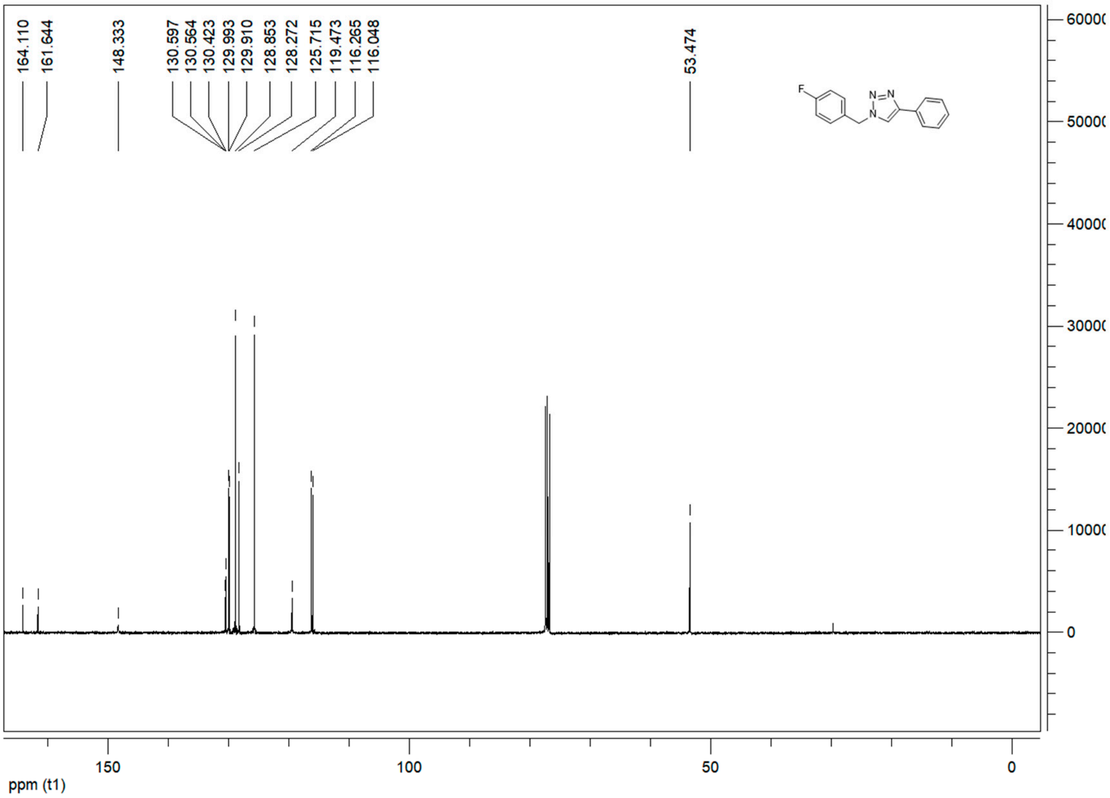

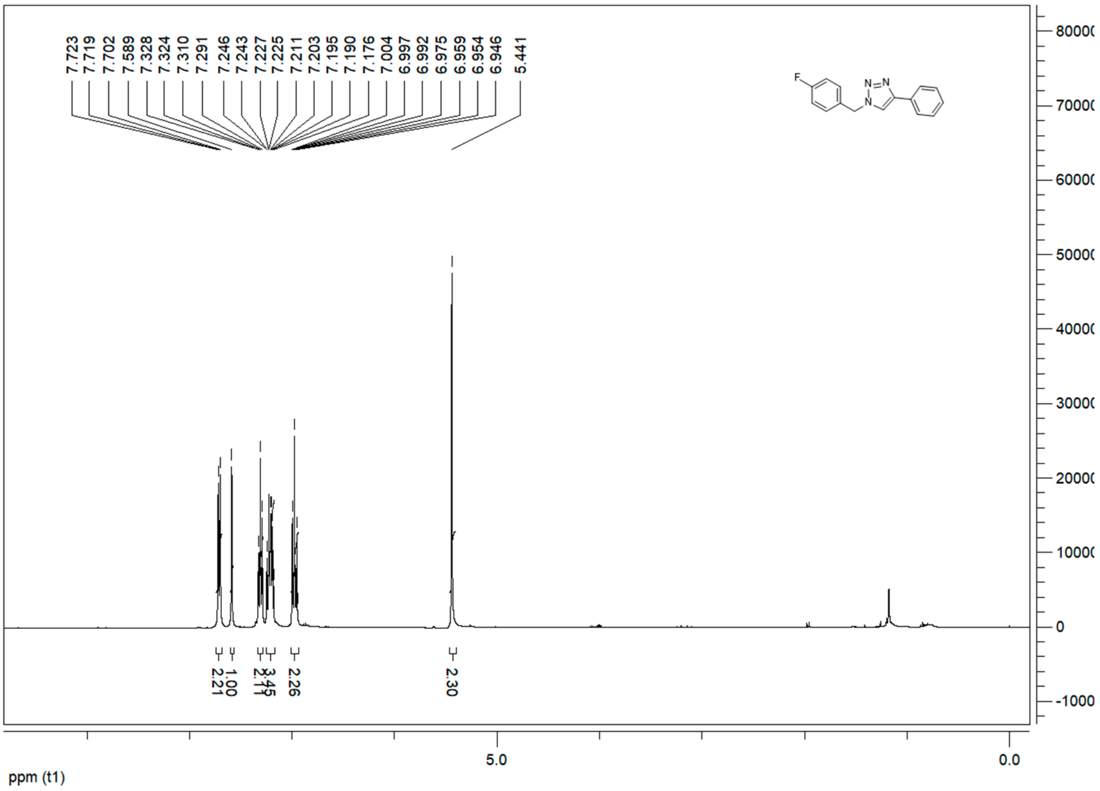

1-(4-(Cyanobenzyl)-4-phenyl-1*H*-1,2,3-triazole (**4e**)

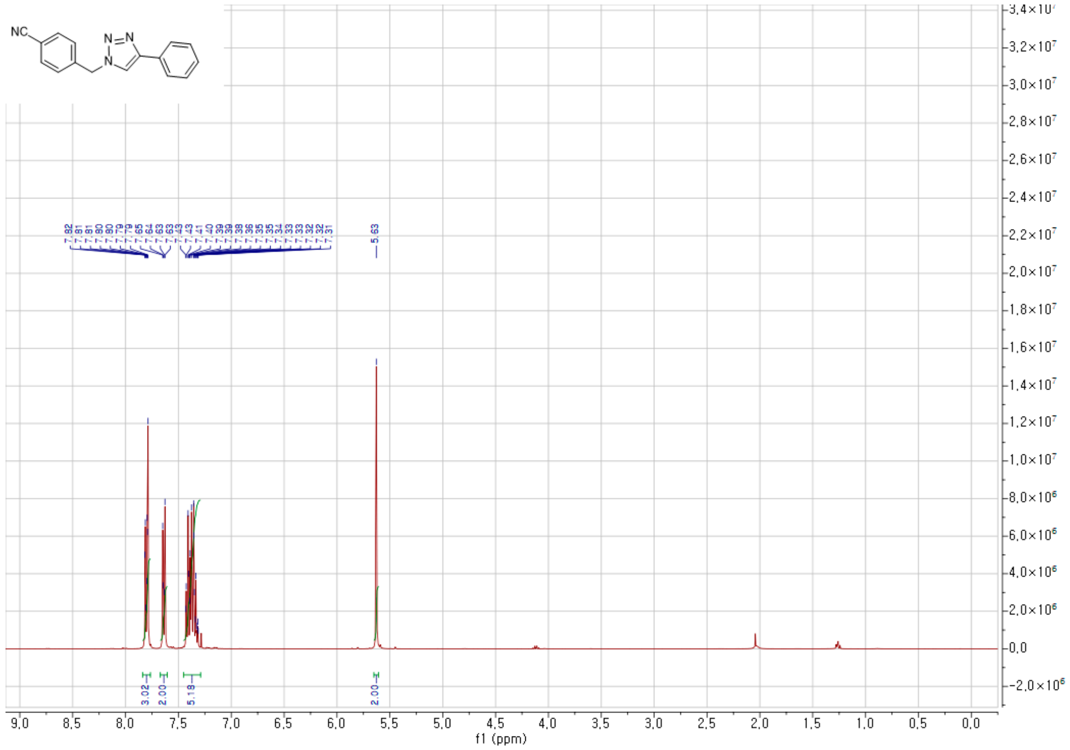

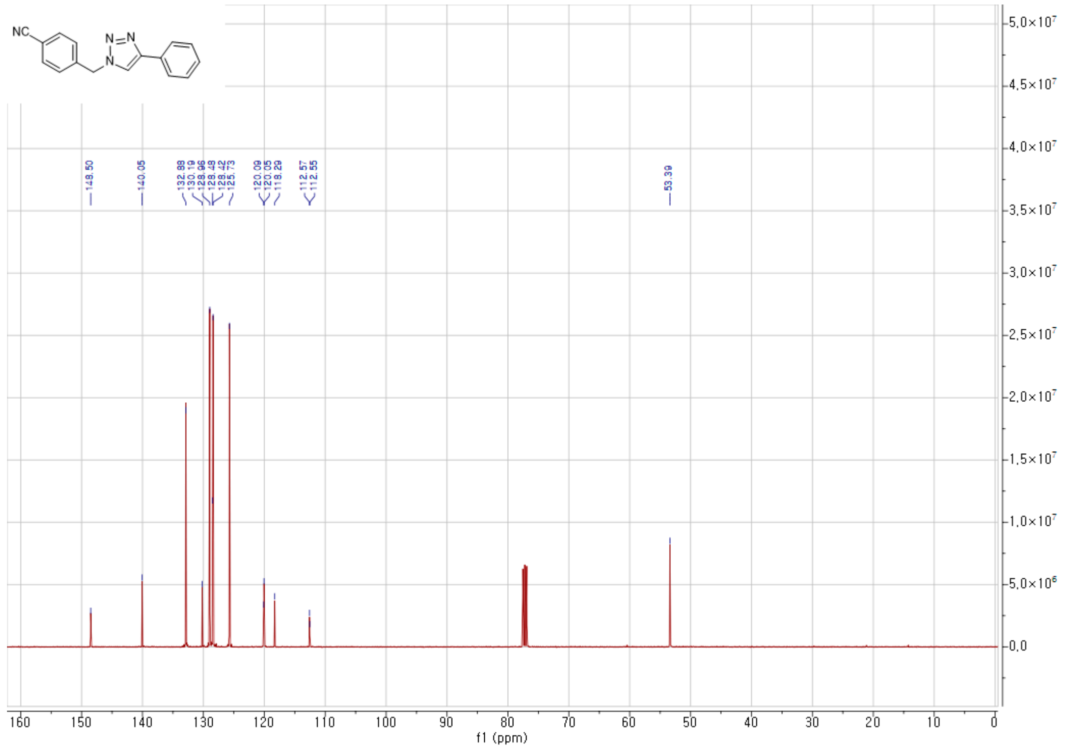

1-(2,4-Dichlorobenzyl)-4-phenyl-1*H*-1,2,3-triazole (**4f**)

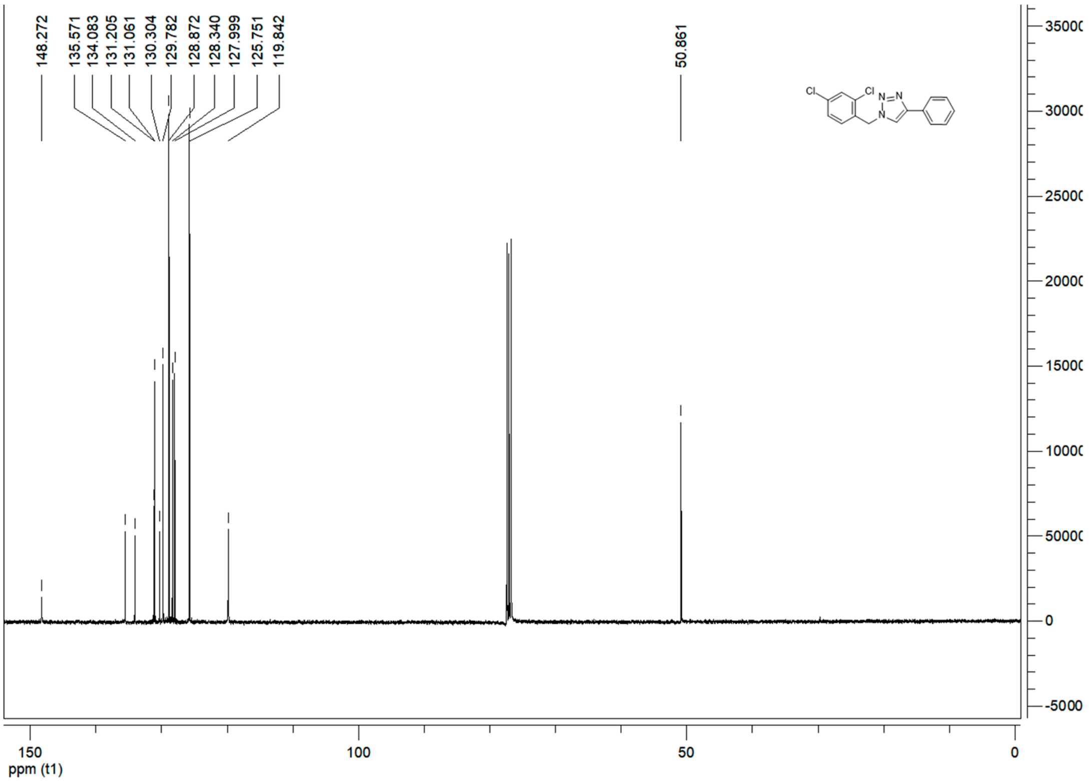

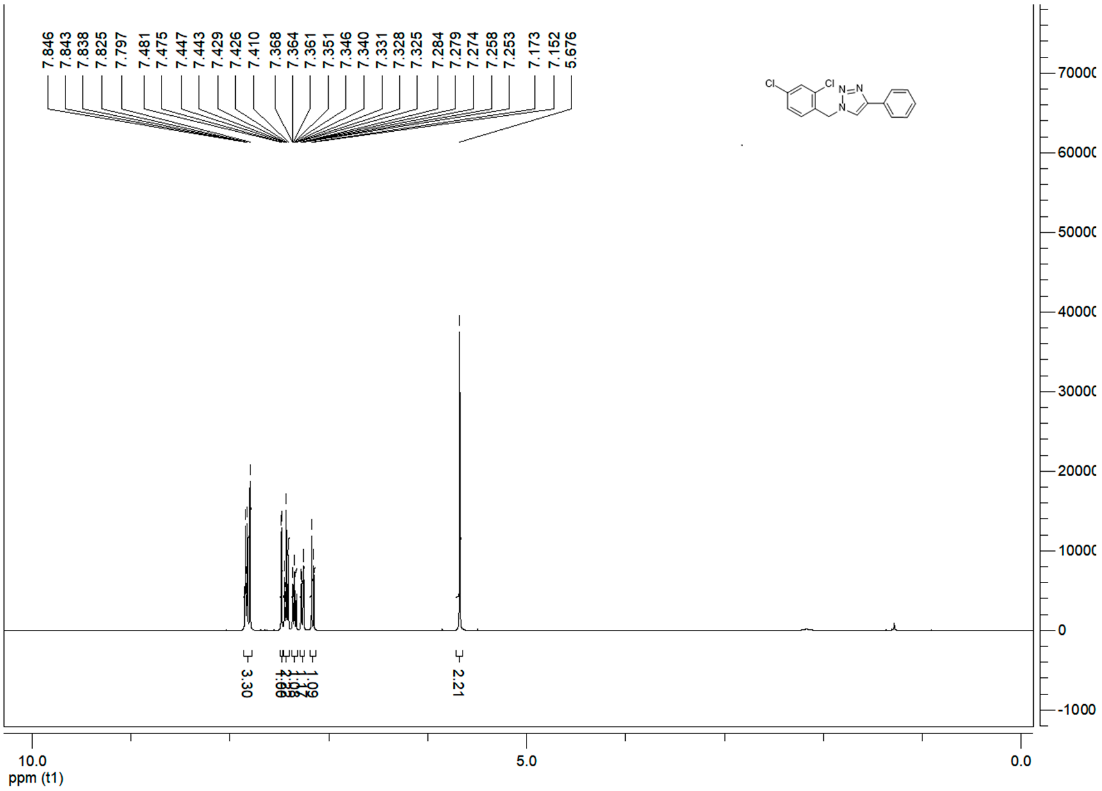

1-Benzyl-4-(4-fluorophenyl)-1*H*-1,2,3-triazole (**4g**)

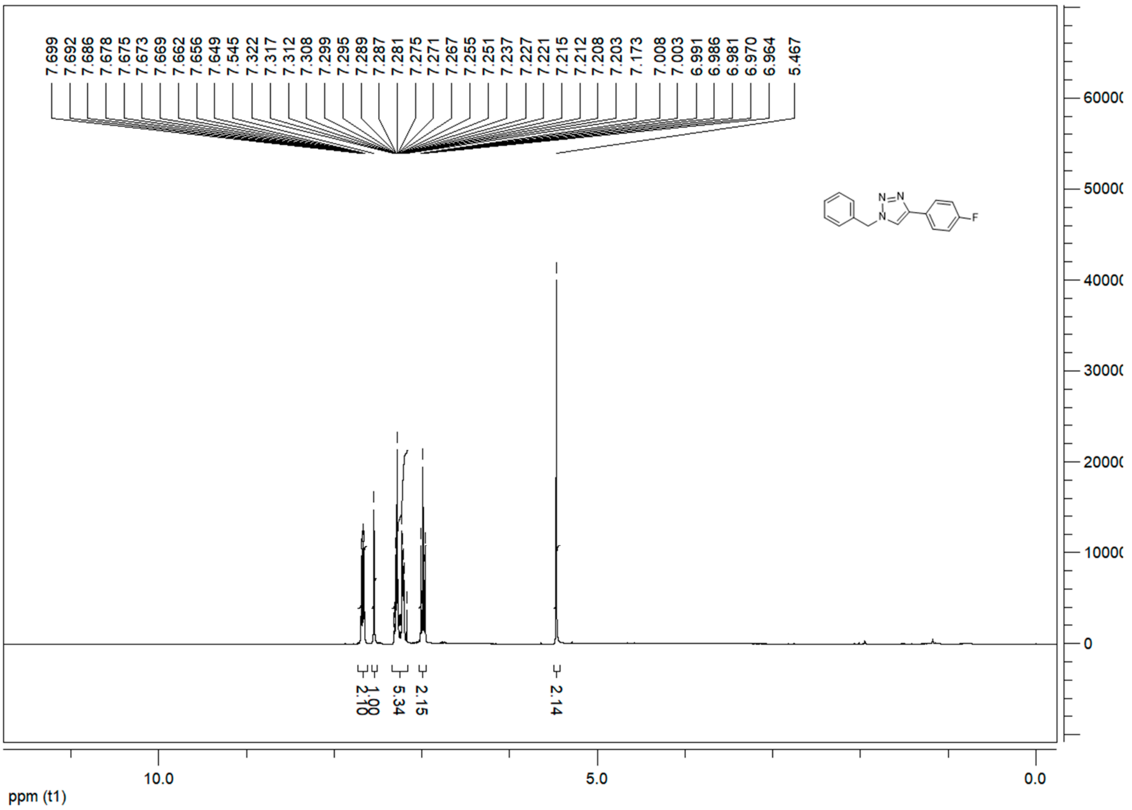

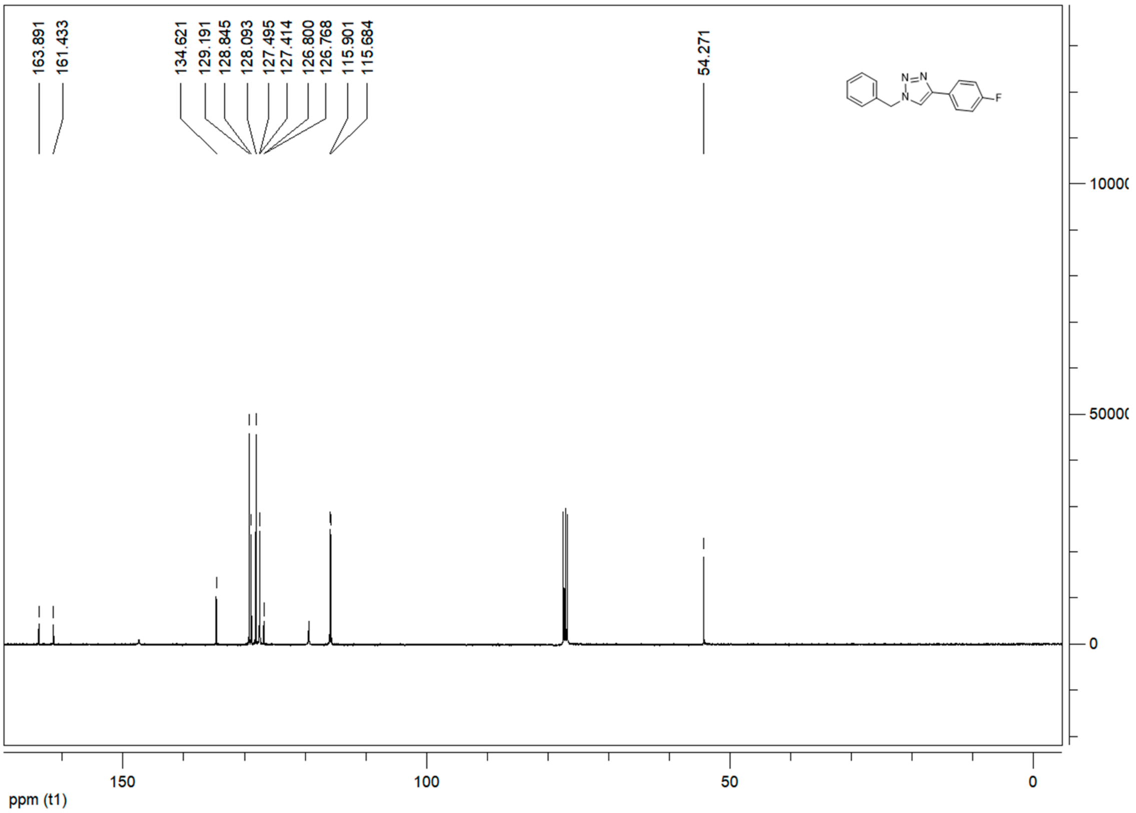

1-Benzyl-4-(4-butylphenyl)-1*H*-1,2,3-triazole (**4h**)

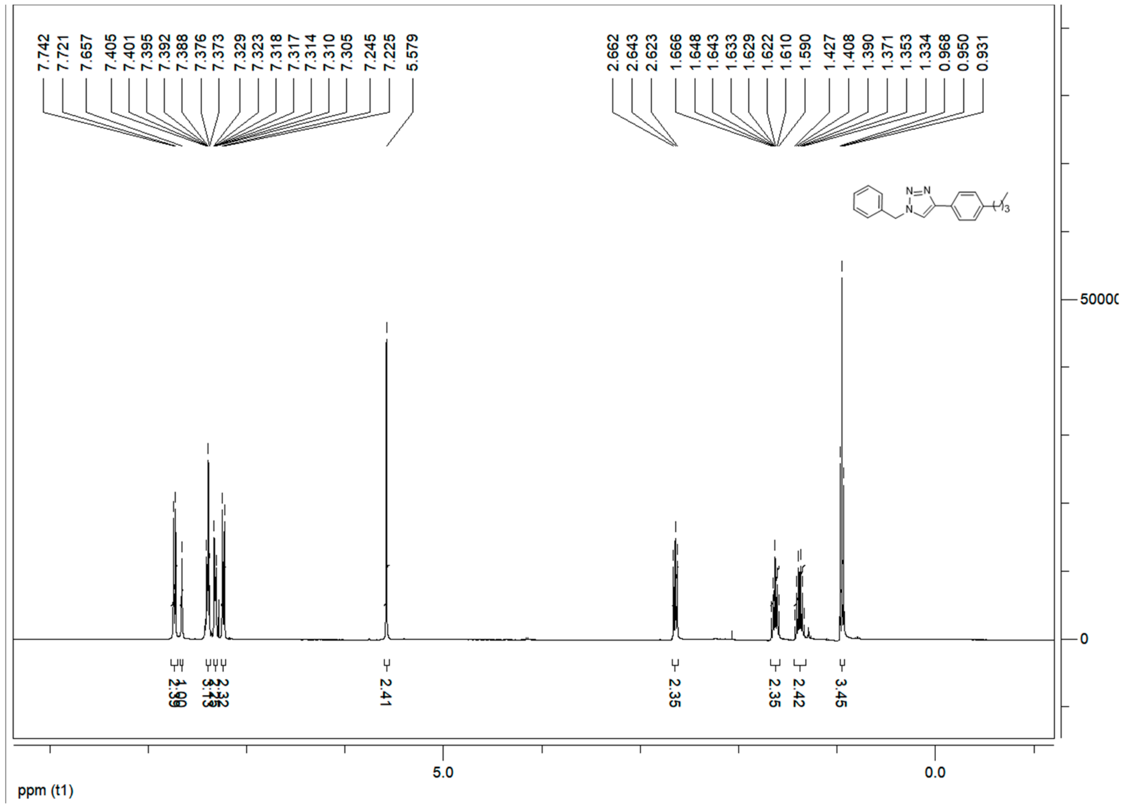

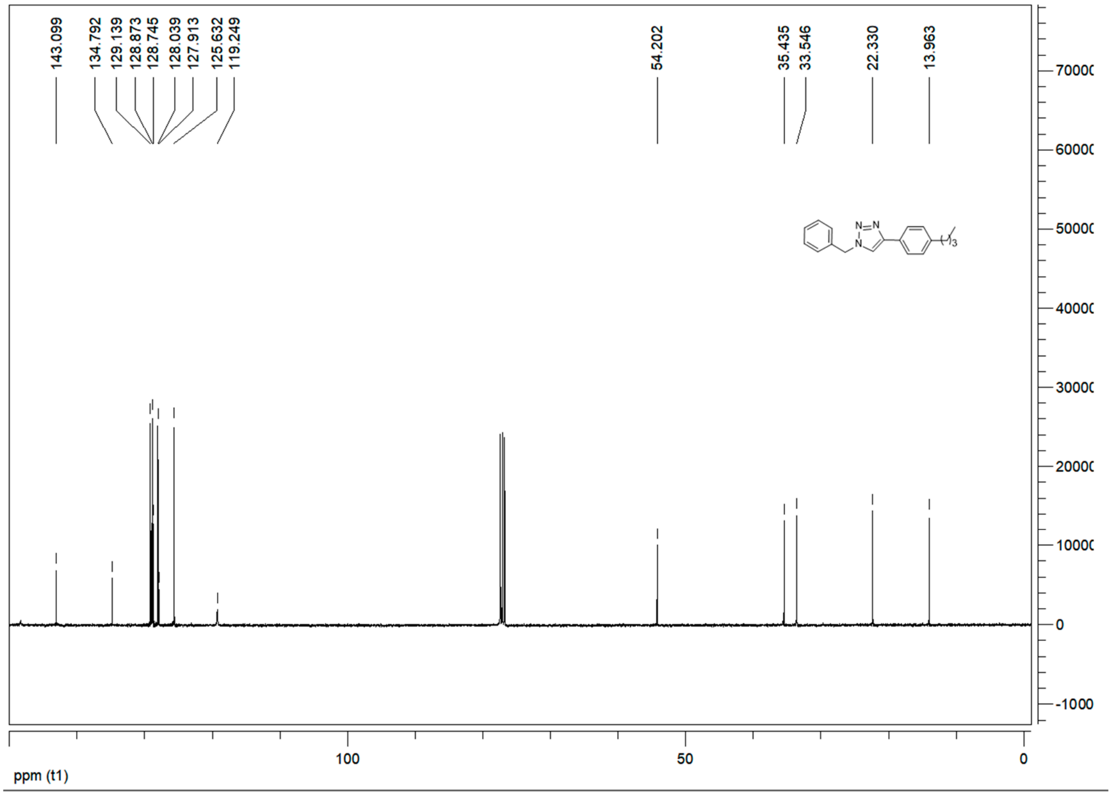

1-Benzyl-4-(4-methoxyphenyl)-1*H*-1,2,3-triazole (**4i**)

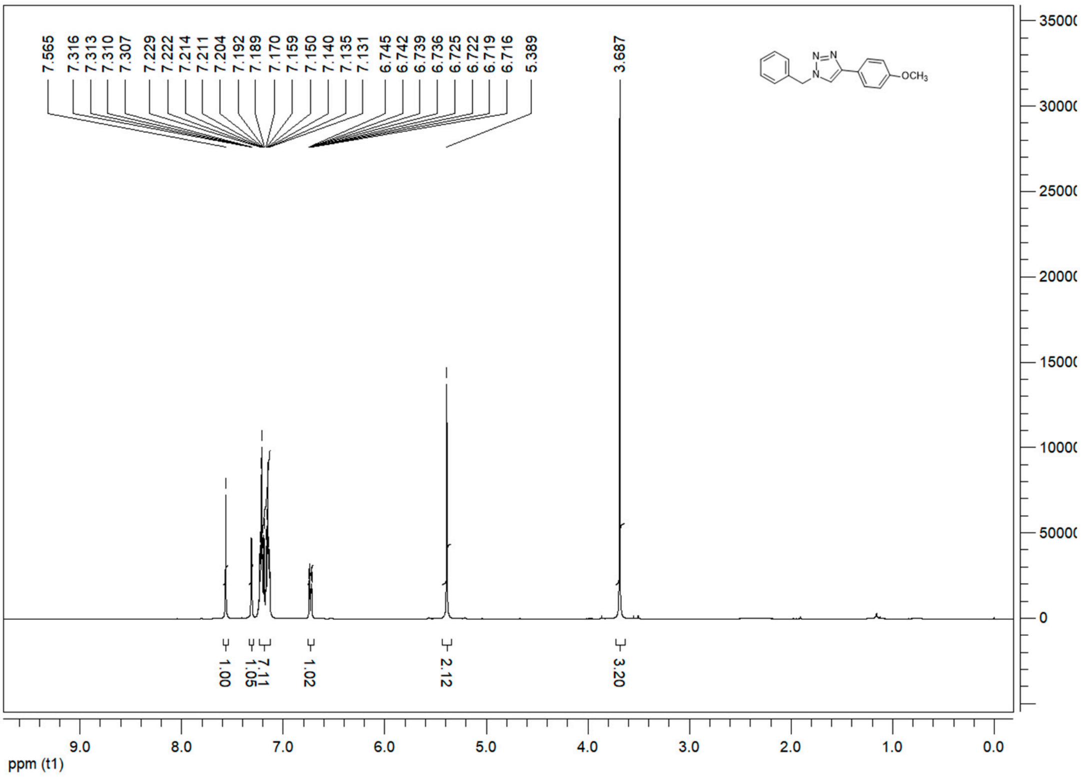

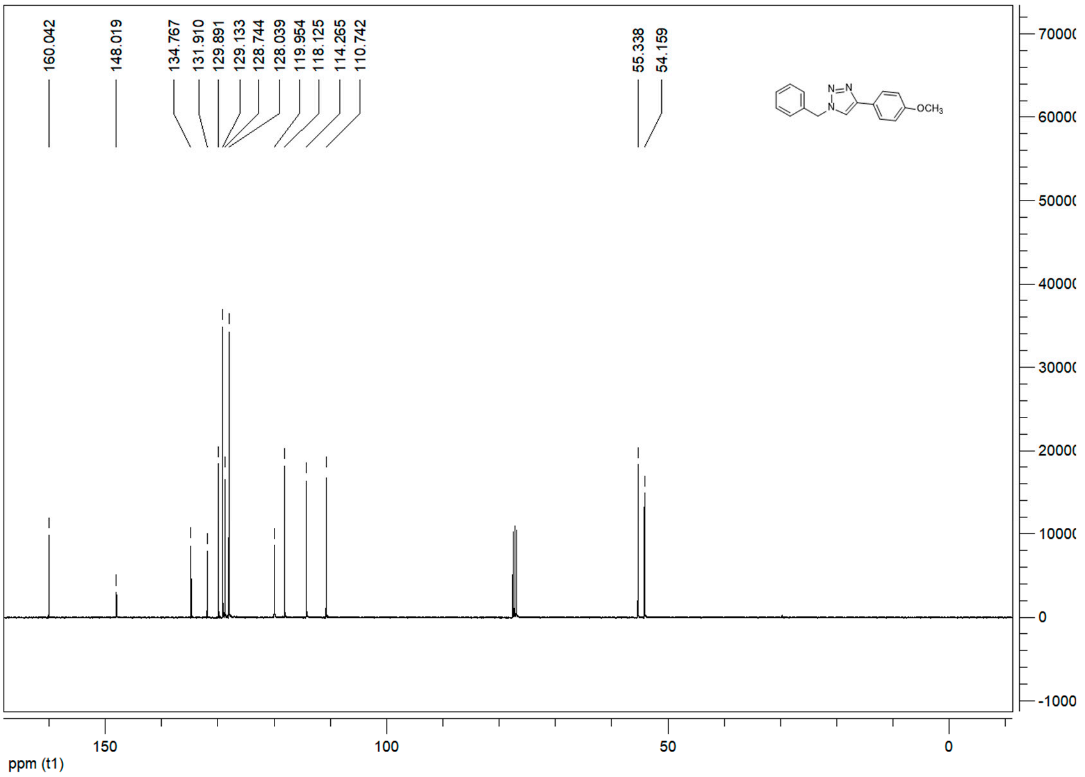

1-Benzyl-4-(4-nitrophenyl)-1*H*-1,2,3-triazole (**4j**)

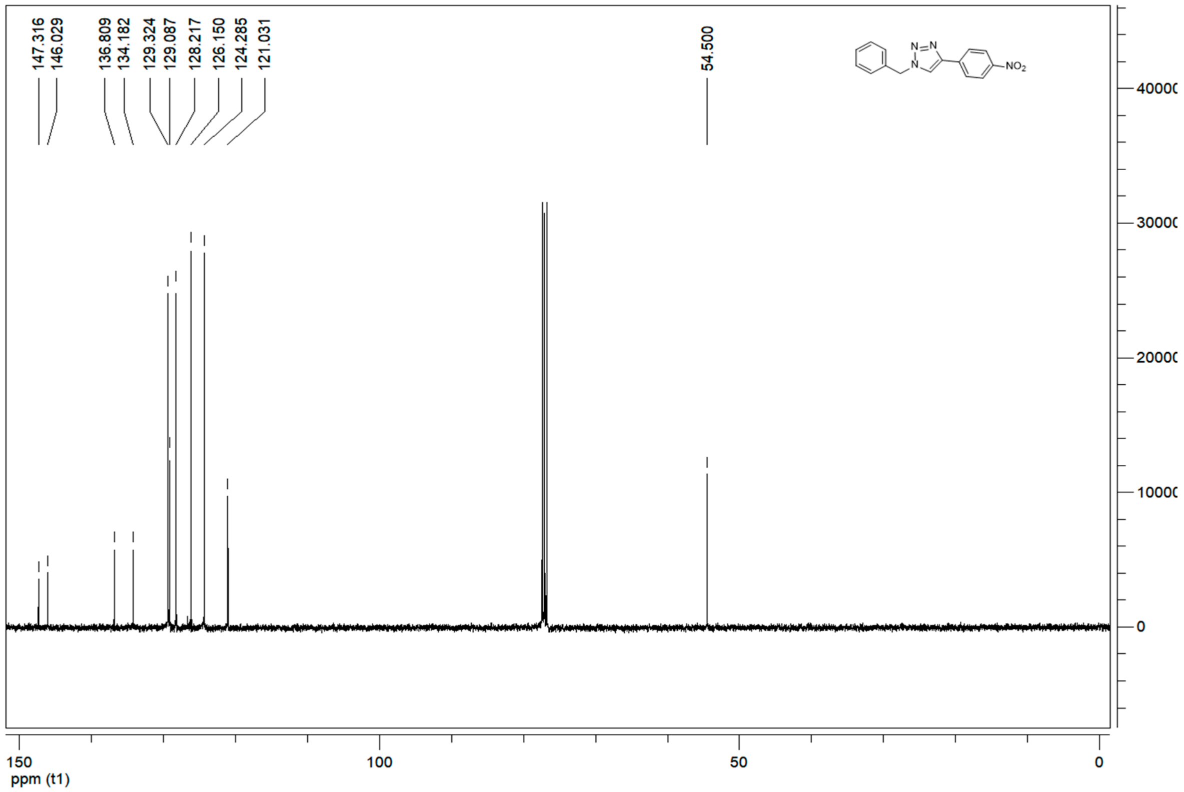

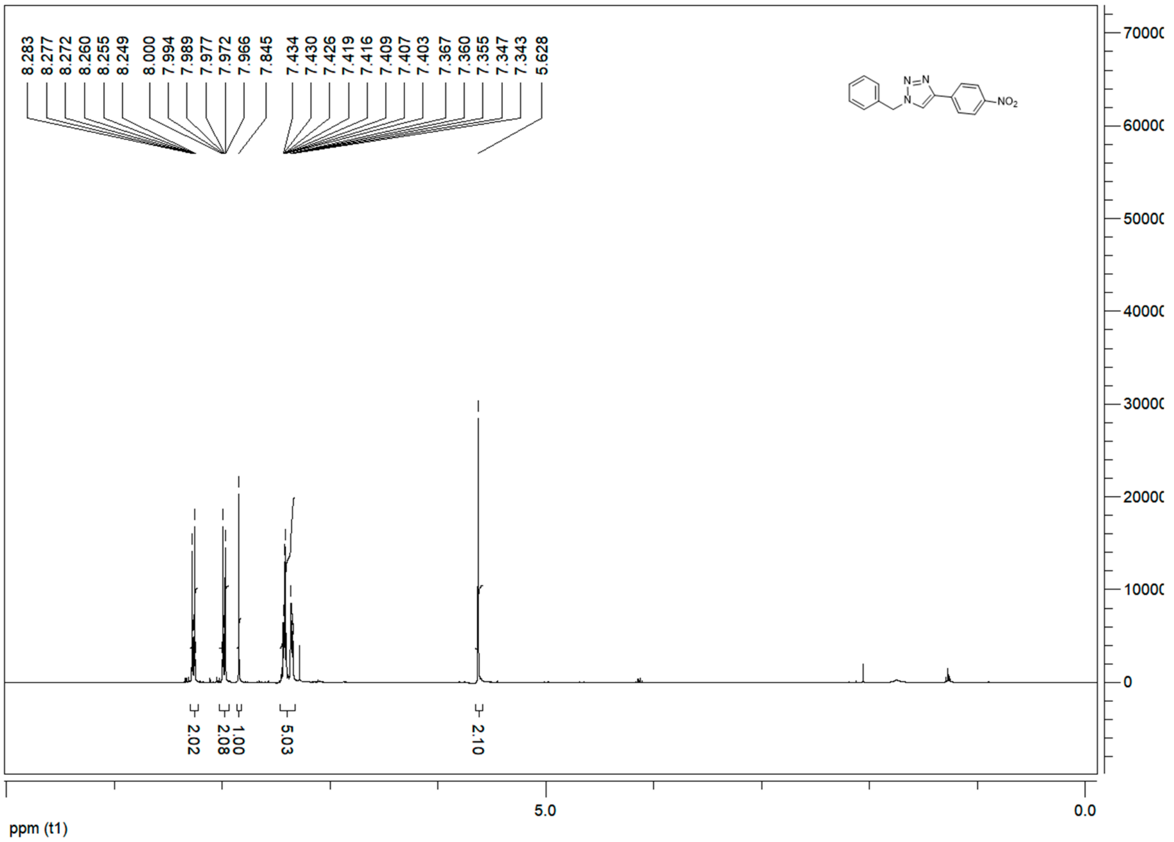

3-(1-Benzyl-1*H*-1,2,3-triazol-4-yl)phenol (**4k**)

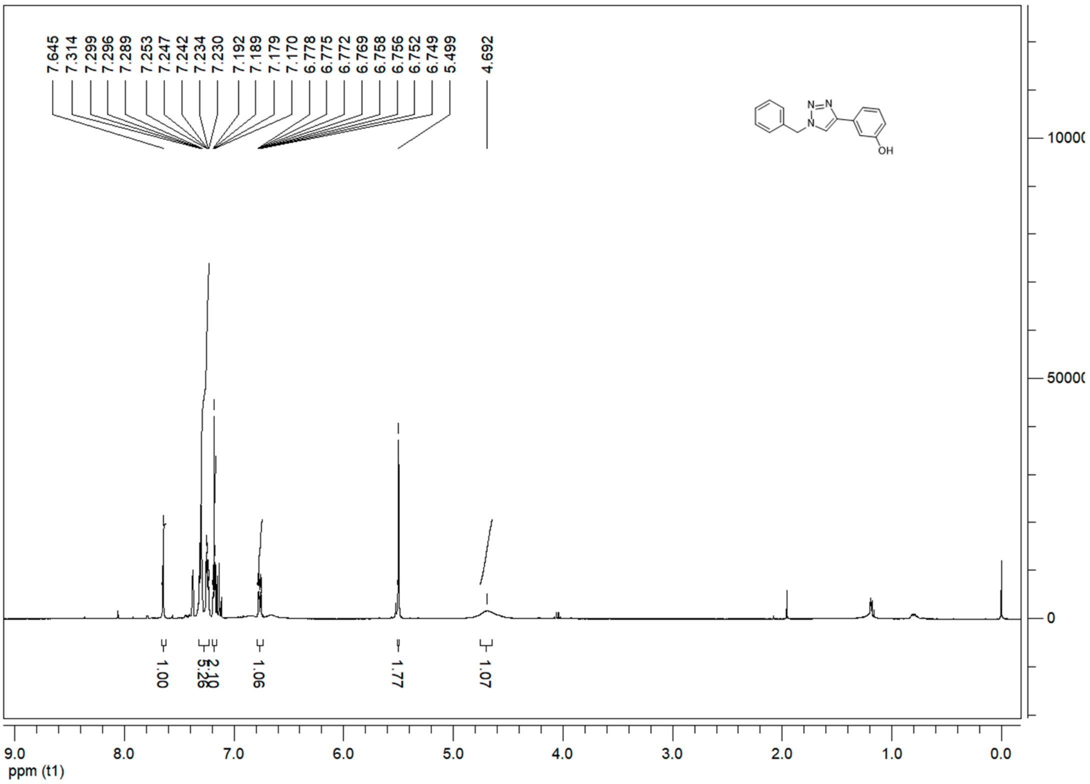

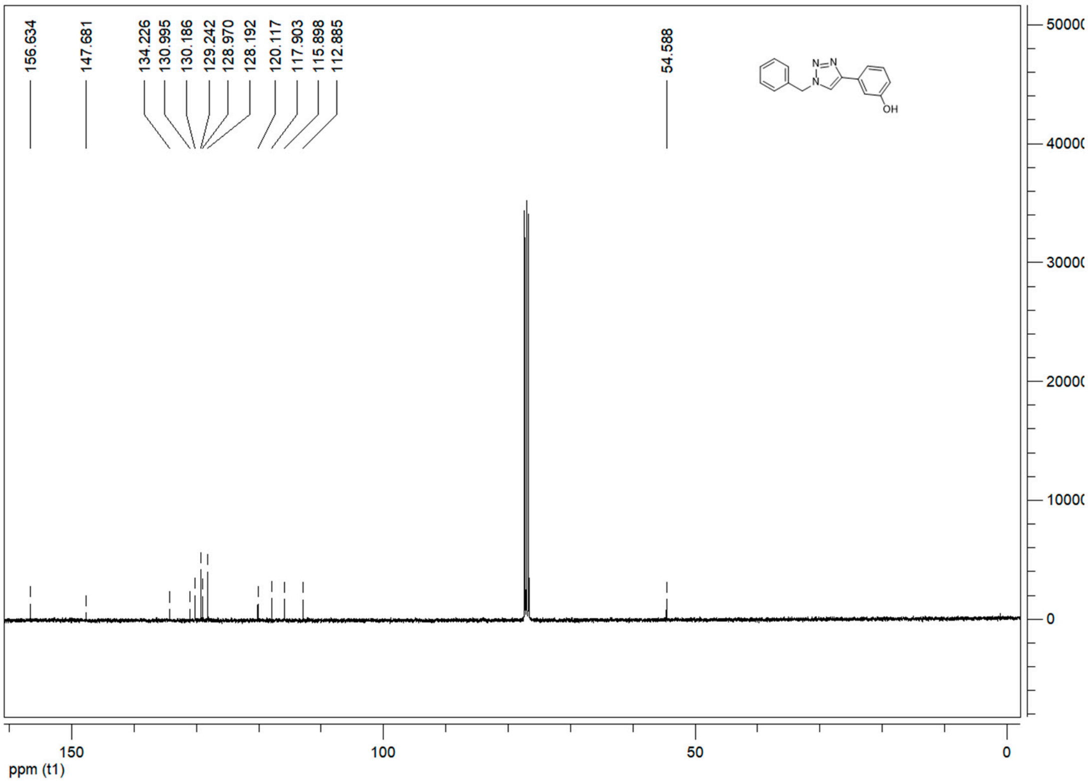

1-(4-Fluorobenzyl)-4-(4-nitrophenyl)-1*H*-1,2,3-triazole (**4l**)

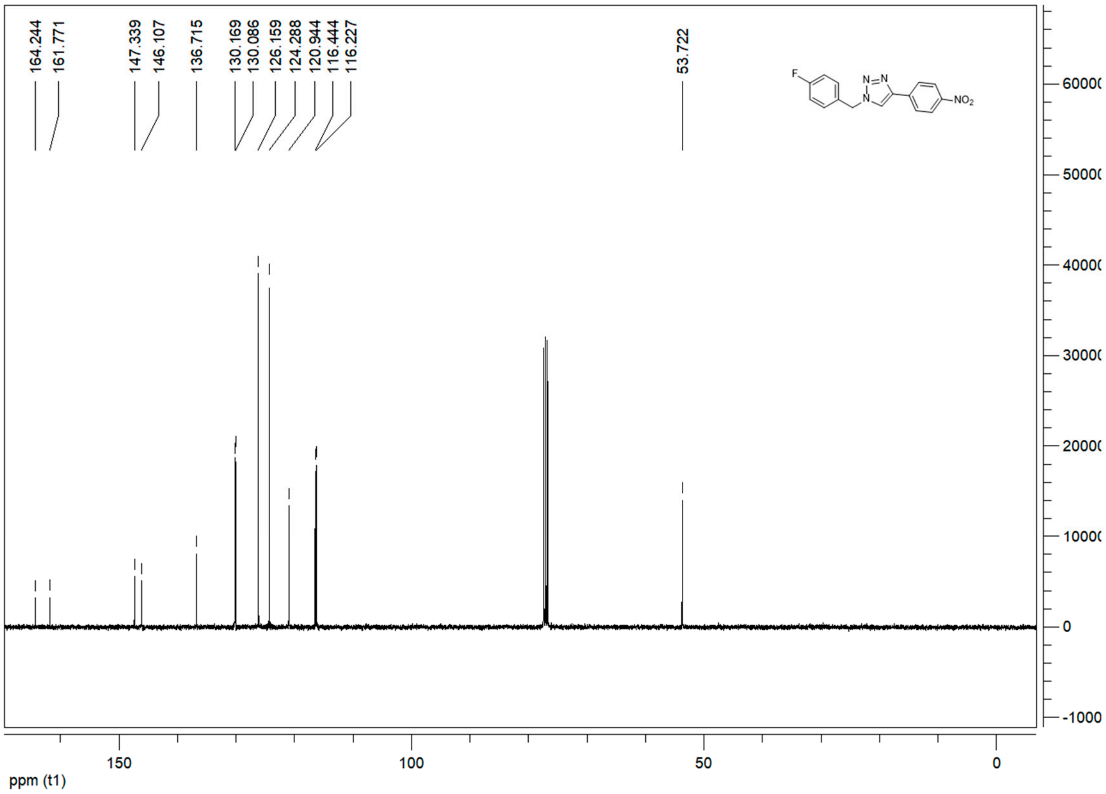

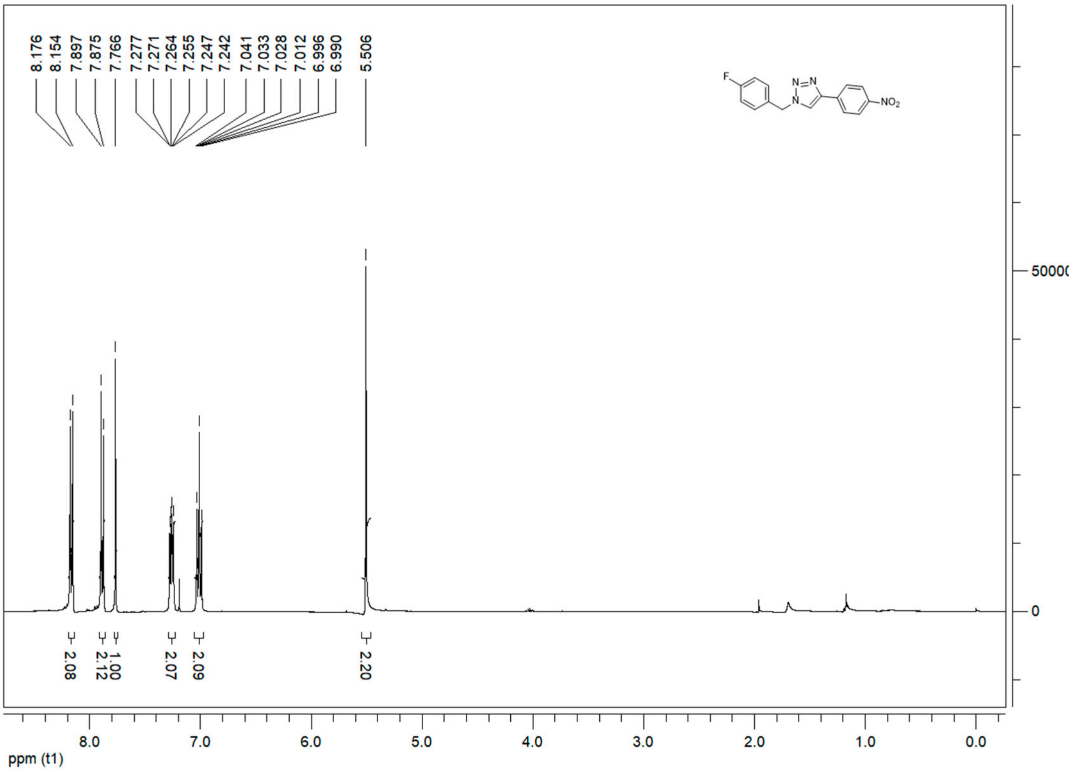

1-(2,4-Dichlorobenzyl)-4-(4-fluorophenyl)-1*H*-1,2,3-triazole (**4m**)

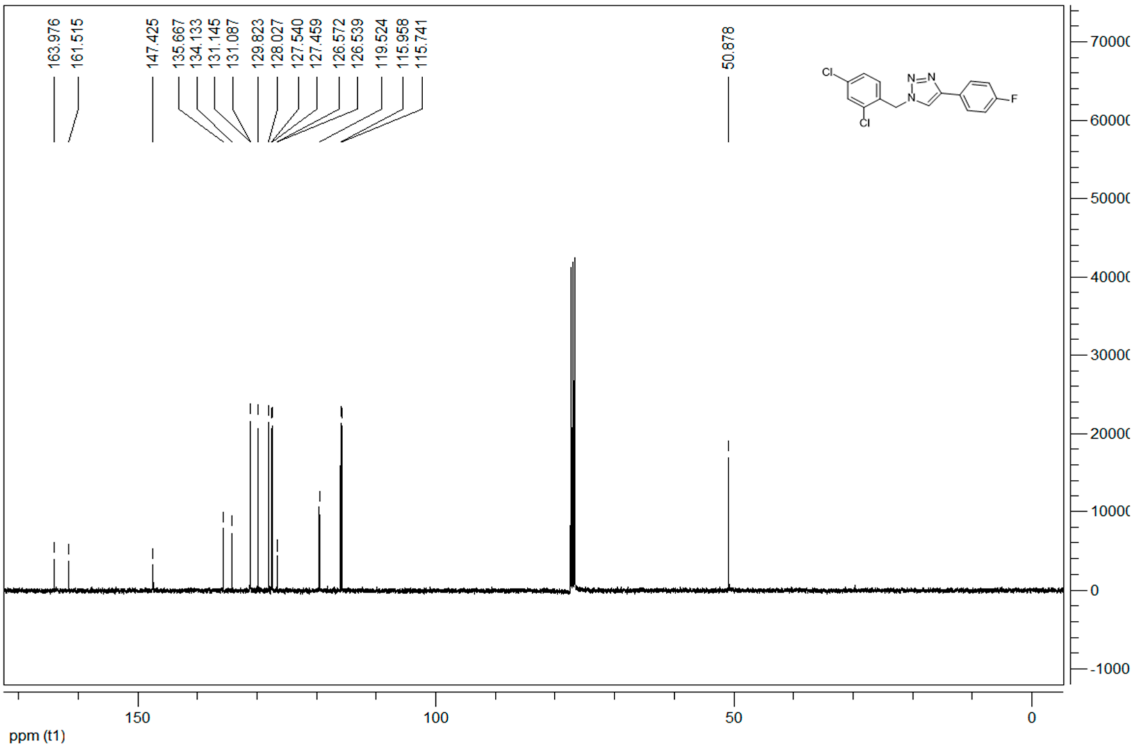

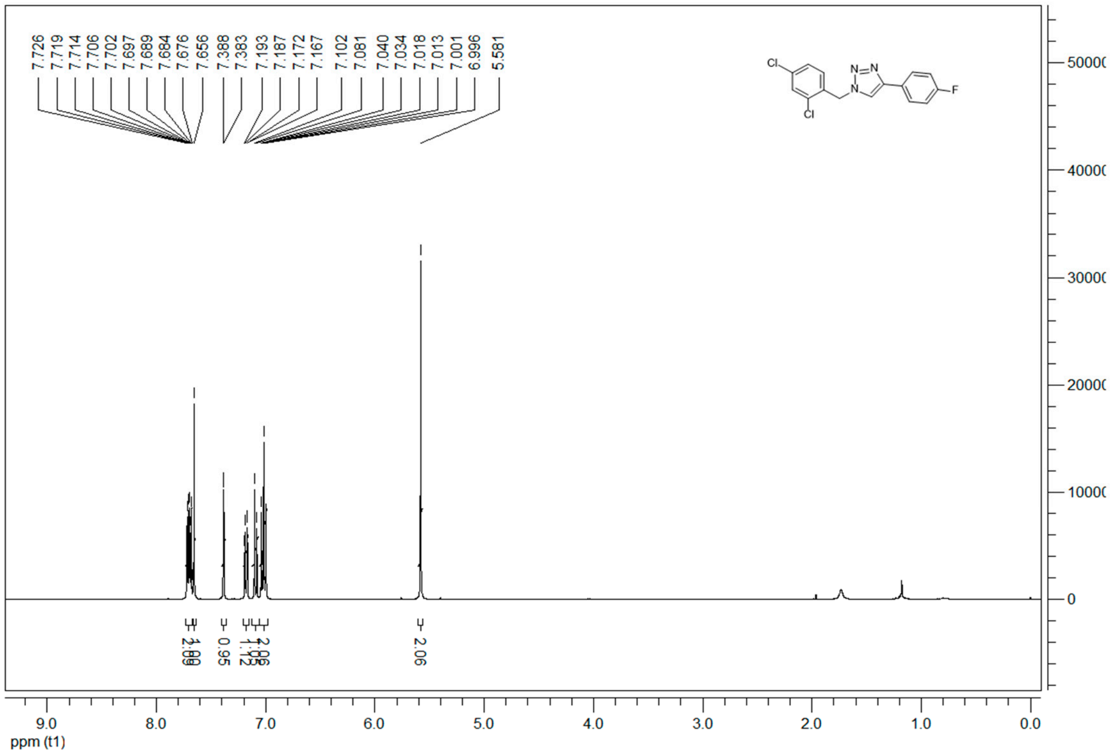

1-(4-(*tert*-Butyl)benzyl)-4-(m-tolyl)-1*H*-1,2,3-triazole (**4n)**

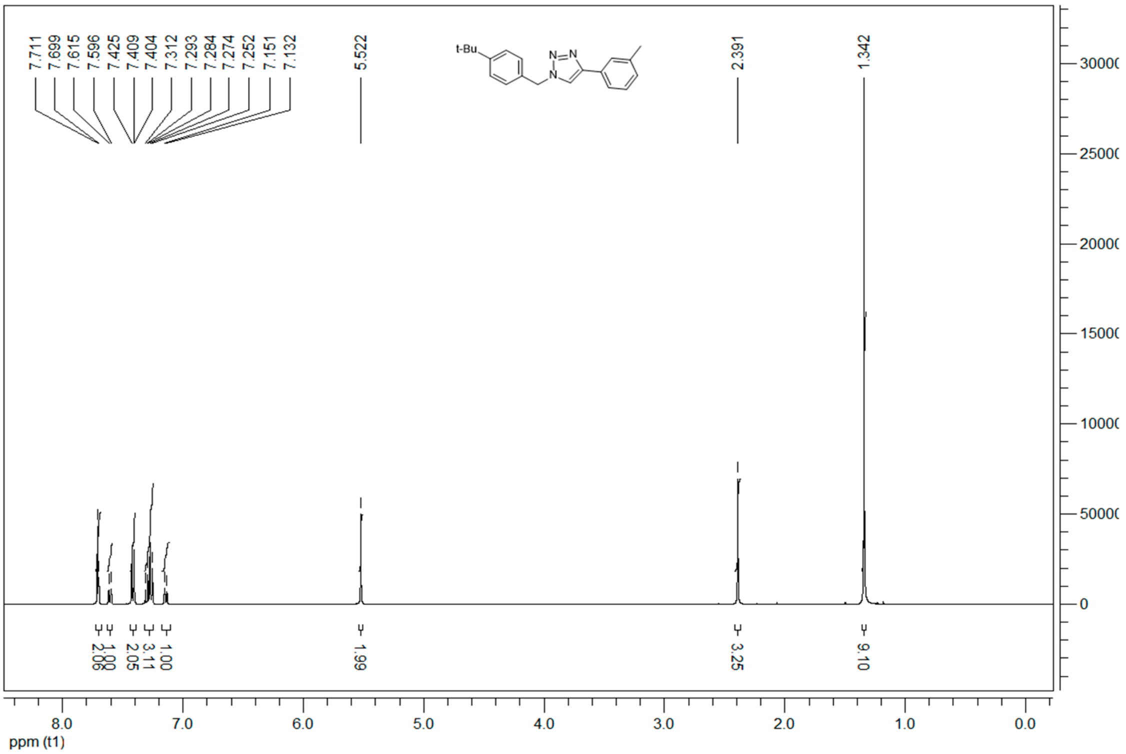

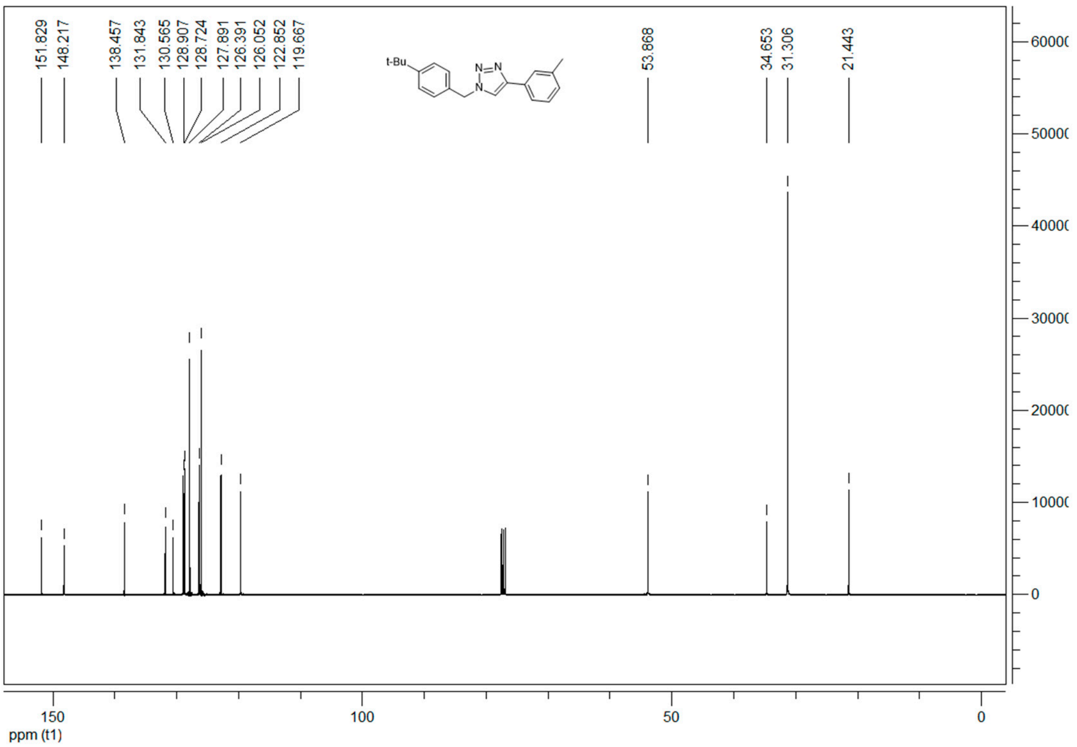

Reference

[1] Hemmati, S.; Zangeneh, M.M.; Zangeneh, A. $CuCl_2$ anchored on polydopamine coated-magnetic nanoparticles (Fe_3O_4@PDA/Cu(II)): Preparation, characterization and evaluation of its cytotoxicity, antioxidant, antibacterial, and antifungal properties *Polyhedron* **2020**, *177*, 114327.
